# Florigen and cytokinin signaling antagonistically regulate FLOWERING LOCUS T-LIKE1 to drive a florigen relay that facilitates inflorescence development in rice

**DOI:** 10.1126/sciadv.adv1424

**Published:** 2025-12-19

**Authors:** Moeko Sato, Yuki Sakamoto, Mari Tanaka, Jun Ito, Yuko Nomura, Yurika Morishita, Ken-ichiro Taoka, Masafumi Mikami, Masaki Endo, Hidemi Kitano, Sachihiro Matsunaga, Hiroyuki Tsuji

**Affiliations:** ^1^ Kihara Institute for Biological Research, Yokohama City University, Maioka 641-12, Totsuka, Yokohama 244-0813, Japan.; ^2^ Imaging Frontier Center, Organization for Research Advancement, Tokyo University of Science, 2641 Yamazaki, Noda, Chiba 278-8510, Japan.; ^3^ Department of Biological Sciences, Graduate School of Science, Osaka University, Machikaneyama-cho 1-1, Toyonaka, Osaka 560-0043, Japan.; ^4^ Department of Biology, Faculty of Science, Shinshu University, Asahi 3-1-1, Matsumoto, Nagano 390-8621, Japan.; ^5^ Graduate School of Bioagricultural Sciences, Nagoya University, Nagoya, Aichi 464-8601, Japan.; ^6^ Graduate School of Nanobioscience, Yokohama City University, 22-2 Seto, Yokohama, Kanagawa 236-0027, Japan.; ^7^ Institute of Agrobiological Sciences, National Agriculture and Food Research Organization, 1-2 Owashi, Tsukuba, Ibaraki 305-8634, Japan.; ^8^ Bioscience and Biotechnology Center, Nagoya University, Nagoya, Aichi 464-8601, Japan.; ^9^ Department of Applied Biological Science, Faculty of Science and Technology, Tokyo University of Science, 2641 Yamazaki, Noda, Chiba 278-8510, Japan.; ^10^ Department of Integrated Biosciences, Graduate School of Frontier Sciences, The University of Tokyo, 5-1-5 Kashiwanoha, Kashiwa, Chiba 277-8562, Japan.

## Abstract

Plant reproductive development involves a series of transitions, with the shoot apical meristem (SAM) transitioning into the inflorescence meristem (IM) and then the floral meristem (FM). In rice (*Oryza sativa*), the florigens Heading date 3a (Hd3a) and RICE FLOWERING LOCUS T1 play central roles in these transitions. The plant hormones auxin and cytokinin shape the morphologies associated with each transition, but little is known about the factors that integrate florigen function with phytohormone signaling during these transitions. Here, we show that in rice, Hd3a activates the transcription of *FT-LIKE1* (*OsFTL1*), which encodes a mobile FT-like protein that promotes the transition from the IM to the FM, whereas cytokinin signaling suppresses its transcription. We performed single-cell resolution three-dimensional imaging to elucidate the spatiotemporal distribution of Hd3a, cytokinin, and auxin signaling during the SAM-to-IM and IM-to-FM transitions, finding that Hd3a accumulation and cytokinin signaling occupy opposite domains in the IM. Hd3a accumulated at the center of the meristem and activated the *OsFTL1* promoter, while OsFTL1 was present throughout the IM, suggesting that a “florigen relay” consisting of Hd3a from the leaves moves to the SAM and induces *OsFTL1* expression within the SAM. We propose an antagonistic mechanism mediated by Hd3a and cytokinin that modulates the abundance of OsFTL1 and thus regulates reproductive development in rice.

## Teaser

A florigen relay in the shoot apical meristem promotes inflorescence development in rice.

## INTRODUCTION

Plant development involves a series of developmental transitions in the shoot apical meristem (SAM) (*1*–*6*). After germination, the SAM begins the leaf-forming vegetative phase; upon recognizing the appropriate environmental conditions, it transitions to the flower-forming reproductive phase (*1*, *2*). During the reproductive phase, the SAM is first converted to an inflorescence meristem (IM) that produces inflorescence branches, followed by the conversion of the IM into a floral meristem (FM) (*6*–*9*). These transitions are important for plant reproductive success and for increasing crop yield (*4*). These developmental transitions are orchestrated by multilayered regulatory networks that include transcription factors and other regulatory molecules (*1*, *10*). For example, the transcription factors LEAFY, MADS-box, and SQUAMOSA PROMOTER BINDING PROTEIN-LIKEs (SPLs) promote the transition from the IM to the FM (*11*–*15*). Regulatory systems centered on florigens [FLOWERING LOCUS T (FT) in *Arabidopsis* (*Arabidopsis thaliana*) and Heading date 3a (Hd3a) and RICE FLOWERING LOCUS T1 (RFT1) in rice (*Oryza sativa*)] and antiflorigens [TERMINAL FLOWER1 in *Arabidopsis* and RICE CENTRORADIALIS (RCN) in rice] control the SAM-to-IM and IM-to-FM transitions (*5*, *6*, *16*–*19*).

FT and Hd3a are mobile proteins belonging to the phosphatidylethanolamine-binding protein family that play systemic roles in controlling developmental phase transitions (*2*, *6*, *20*–*23*). Environmental and endogenous signals such as day length, temperature, and nutritional availability trigger *FT* and *Hd3a* expression in leaves (*24*–*28*). FT and Hd3a proteins then move from leaves to the SAM, where they interact with the basic leucine-zipper transcription factor FD and 14-3-3 proteins to form an FT/Hd3a–14-3-3–FD complex known as the florigen activation complex (FAC) (*5*, *6*, *29*–*33*). The FAC activates the expression of FM identity genes such as class A MADS-box genes, including *APETALA1* (*AP1*) in *Arabidopsis* and *OsMADS15* in rice, leading to the transition of the SAM to the IM (*29*, *30*, *32*–*34*). FT and its orthologs also control the IM-to-FM transition and thus inflorescence branching: The precocious transition of the IM to an FM decreases the number of inflorescence branches, whereas a delayed transition increases inflorescence branch number (*4*, *5*, *17*). A slight reduction in the activity of florigens such as SINGLE-FLOWER TRUSS in tomato (*Solanum lycopersicum*), FT in *Arabidopsis*, and Hd3a in rice can increase the number of inflorescence branches and optimize crop yield without significantly delaying flowering. (*16*, *18*, *19*, *34*–*37*).

Changing the timing of developmental phase transitions can substantially alter plant morphogenesis (*6*, *38*–*40*). During the reproductive phase, the morphology of lateral organs changes, as does phyllotaxis (the arrangement of leaves along the stem) and the plastochron (the time between the emergence of two leaves). Plant hormones are responsible for these changes (*41*). Local auxin accumulation predicts the region of organ differentiation in the SAM through the formation of local signaling maxima via directional transport by PIN-FORMED–type efflux carriers (*42*–*44*). Mutants impaired in auxin biosynthesis, transport, or action show severe morphological abnormalities in many plant species, e.g., *Arabidopsis*, maize (*Zea mays*), and rice (*41*, *45*–*48*).

Cytokinins regulate cell division, maintain stem cell number in the SAM, and influence the spatial regulation of organ differentiation (*49*–*52*). For example, phyllotaxis is regulated by cytokinins in *Arabidopsis*, maize, and rice (*51*, *53*–*55*). In *Arabidopsis*, phyllotaxis is regulated via diffusion of the cytokinin signaling inhibitor HISTIDINE PHOSPHOTRANSFER PROTEIN6 between organ primordia and their boundaries (*53*). In maize and rice, mutants with perturbed cytokinin signaling display altered phyllotaxis from distichous to decussate (*54*, *55*). In addition to their functions in morphogenesis, cytokinins are involved in transitions between developmental stages (*56*, *57*). In rice, loss-of-function mutants of the cytokinin-degrading enzyme CYTOKININ OXIDASE2 (CKX2) exhibit increased cytokinin accumulation, which delays the conversion of inflorescences to flowers and increases branching (*4*, *57*), suggesting that cytokinins participate in regulating the IM-to-FM transition, which is thought to be controlled by florigen. However, little is known about the spatiotemporal changes in auxin and cytokinin signaling during developmental phase transitions or how florigen functions are integrated with phytohormone signaling during these phase transitions.

In this study, we explored the spatiotemporal dynamics of auxin and cytokinin signaling during the reproductive transition of rice meristems and showed that florigen activates *OsFTL1* expression in the IM, whereas cytokinin signaling suppresses the expression of this gene (*58*–*60*). Through three-dimensional (3D) imaging of the SAM at single-cell resolution, we explored the changes in the spatiotemporal distribution of florigen, cytokinin signaling, and auxin signaling during phase transitions. Transcriptome and genetic analyses revealed that Hd3a activates *OsFTL1* expression, whereas cytokinin signaling represses its expression. The *OsFTL1* promoter is activated in specific cells in the IM, and OsFTL1 is present throughout the IM to control inflorescence development, suggesting that OsFTL1 is a short-range mobile integrator of cytokinin signaling and florigen function during the IM-to-FM transition.

## RESULTS

### Cells with active cytokinin signaling surround cells with active auxin signaling at the site of organ differentiation of the rice SAM during the vegetative phase

To study the spatial distribution of active cytokinin and auxin signaling, we generated *TCSv2:tdTomato* and *DR5rev:NLS-3xVenus* transgenic rice plants (*61*–*65*), which exhibited cytokinin- and auxin-responsive fluorescence patterns in roots consistent with those previously reported in *Arabidopsis* (*65*), and used these lines to produce double transgenic lines (fig. S1).

**Fig. 1.**
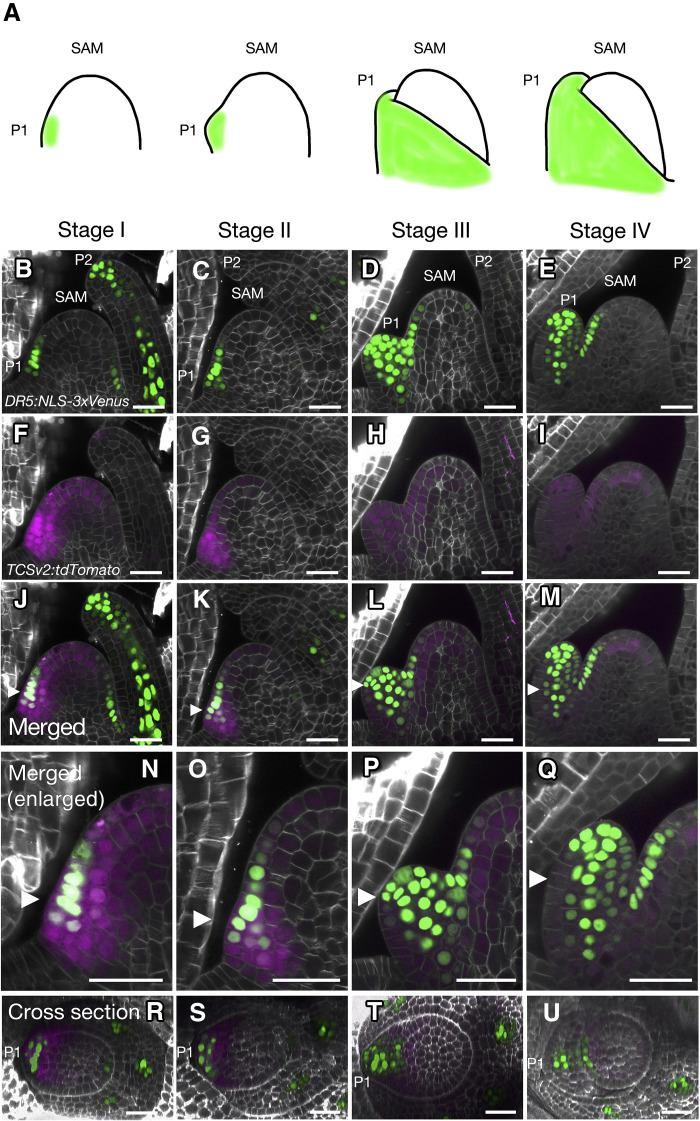
Cells with active cytokinin signaling surround cells with active auxin signaling at the site of organ differentiation during the vegetative phase in the rice SAM. (**A**) Diagram of the rice shoot apical meristem (SAM) at different stages of leaf primordium development. Leaf primordia are shown in green. P1, youngest leaf primordium (plastochron 1). (**B** to **U**) Imaging of the SAM from *DR5rev:NLS-3xVenus TCSv2:tdTomato* double transgenic rice lines. Venus signal from *DR5rev:NLS-3xVenus* (B to E), tdTomato signal from *TCSv2:tdTomato* (F to I), merged images (J to M), enlarged views of (J) to (M) (N to Q), and cross sections from 3D reconstructions of the merged images (R to U) for stage I (B, F, J, N, and R), stage II (C, G, K, O, and S), stage III (D, H, L, P, and T), and stage IV (E, I, M, Q, and U). SAM, shoot apical meristem; P2, second youngest leaf primordium (plastochron 2). Arrowheads in (J) to (Q) indicate the positions of cross sections in (R) to (U). All observations were replicated using more than 10 SAMs from independent plants, and representative images are shown. Scale bars, 25 μm.

**Fig. 2.**
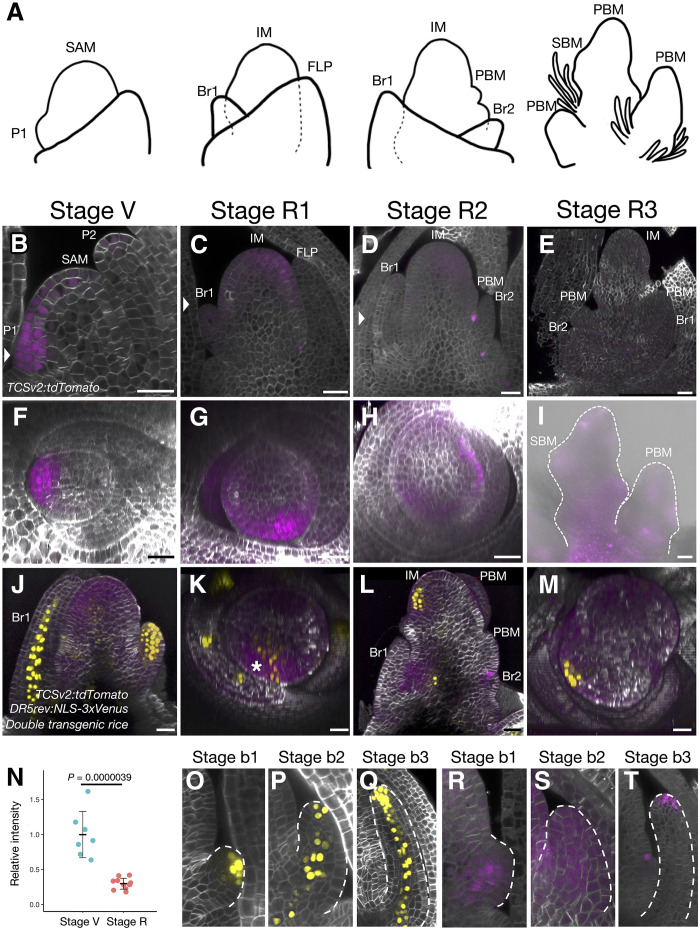
Active cytokinin signaling extends to more cells but is weakened in the IM during the reproductive phase. (**A**) Diagrams of the rice IM at different stages of development. SAM, shoot apical meristem; P1, youngest leaf primordium (plastochron 1); IM, inflorescence meristem; FLP, flag leaf primordium; Br1, first bract; Br2, second bract; PBM, primary branch meristem; SBM, secondary branch meristem. (**B** to **I**) Imaging of the SAM and the IM of *TCSv2:tdTomato* transgenic rice. tdTomato signals (B to I) in longitudinal sections from z-stacks and cross sections from 3D reconstructions (F to H) for stage V (B and F), stage R1 (C and G), stage R2 (D and H), and stage R3 (E and I). Samples in (B) to (H) were cleared. (**J** to **M**) Imaging of the IM from the *DR5rev:NLS-3xVenus TCSv2:tdTomato* double transgenic rice line. For stage R2, a longitudinal section (J) and cross section (K) are shown. For stage R3, a longitudinal section (L) and cross section (M) are shown. Yellow and magenta signals in (M) to (P) indicate Venus from *DR5rev:NLS-3xVenus* and tdTomato from *TCSv2:tdTomato*, respectively. The asterisk indicates an undifferentiated region in the IM. (**N**) Quantification of fluorescence intensity of tdTomato from *TCSv2:tdTomato* in the SAM at the vegetative (V) and reproductive (R, including R2 and R3) stages. Values are means ± SD (*n* = 7 for stage V and *n* = 10 for stage R); *P* values were determined by a Student’s *t* test. (**O** to **T**) Imaging of bracts from *DR5rev:NLS-3xVenus* (O to Q) and *TCSv2:tdTomato* (R to T) transgenic rice lines at stage b1 (O and R), stage b2 (P and S), and stage b3 (Q and T). All observations were replicated using 3 to 20 independent plants, and representative images are shown. Scale bars, 25 μm.

We observed the SAMs in *TCSv2:tdTomato DR5rev:NLS-3xVenus* double transgenic rice plants (Fig. 1 and movies S1 to S4). We defined four stages of the SAM (stages I to IV) based on the development of leaf primordia (Fig. 1A). As per convention, the youngest leaf primordium was termed plastochron 1 (P1); the second and third youngest primordia were termed P2 and P3, respectively. At stage I, we observed anticlinal divisions in P1 (Fig. 1, B, F, J, N, and R). We detected the Venus signal from the *DR5rev:NLS-3xVenus* transgene in three to four cells longitudinally in the center of P1 (Fig. 1B). The tdTomato signal from the *TCSv2:tdTomato* transgene was detected in DR5-positive cells as well as adjacent cells, including the L1 and subepidermal layers in the apical region of the SAM (Fig. 1, J, N, and R, and movie S1). At stage II, P1 began to grow and formed a small bulge on one side of the SAM (Fig. 1A). The Venus and tdTomato signals extended inward from the L1 cell layer to four to five cells (Fig. 1, C, G, K, O, and S, and movie S2). At stage III, the height of P1 reached half the height of the SAM (Fig. 1A). At this stage, we detected Venus fluorescence on the adaxial side of P1, while we observed the tdTomato signal throughout P1 (Fig. 1, D, H, L, P, and T, and movie S3). At stage IV, the height of P1 was comparable to that of the SAM (Fig. 1A). We detected the Venus signal in the central part of P1, which we presume marks the expected region of vascular differentiation (Fig. 1, E, M, Q, and U) (*66*). We also observed the tdTomato signal throughout P1 and in the subepidermal layer of the SAM (Fig. 1I and movie S4) and at the tip of P2 (Figs. 1F and 2B).

To confirm the dynamic changes in auxin signaling output reported by the *DR5rev:NLS-3xVenus* transgene, we developed transgenic rice lines carrying *DII-Venus* (*67*), a reporter that depends on auxin and reflects auxin-induced degradation of the DII domain of Auxin/INDOLE-3-ACETIC ACID INDUCIBLE (Aux/IAA) transcriptional regulators of auxin signaling. This reporter produces a low Venus signal at sites of high auxin signaling and a high Venus signal at sites of low auxin signaling. We observed Venus fluorescence in SAMs of the *DII-Venus* transgenic rice lines in detail (fig. S2 and movie S5). During stages I and II, Venus fluorescence was weaker in P1 than in the SAM. At stage III, the Venus fluorescence intensity also decreased in the center of P1. Thus, the distribution of auxin signaling, as indicated by the *DII-Venus* reporter, was consistent with that indicated by the *DR5rev:NLS-3xVenus* reporter. These results suggest that auxin and cytokinin signaling are spatially and temporally regulated in the vegetative SAM. Spatially, auxin signaling is localized to sites of organ differentiation, whereas cytokinin signaling is distributed more broadly, including in the leaf primordia and at the tip of the SAM. Temporally, cytokinin signaling is consistently present at the SAM tip, while both cytokinin and auxin signaling appear simultaneously in the initial cells of emerging leaf primordia.

### Reproductive meristems have an increase in active cytokinin signaling area, but a decrease in signal intensity compared to vegetative meristems

To investigate the distribution of active cytokinin and auxin signaling in the IM during reproductive growth, we characterized the fluorescence of the above reporters in IMs of the transgenic rice lines (Fig. 2, fig. S3, and movies S6 to S11). During the vegetative phase, the SAM produces leaves, and an axillary meristem is generated at the axil of each leaf. By contrast, during the reproductive phase, the IM produces bracts, which are degenerate leaves that specifically form during the reproductive stage, while a primary branch meristem (PBM) forms at the axil of each bract (*6*, *40*). The PBM again produces bracts, and a secondary branch meristem or a spikelet meristem forms at each bract axil (Fig. 2A). The primary and secondary branch meristems are converted into terminal spikelet meristems.

We observed the active cytokinin signaling domain in detail, as shown by the *TCSv2:tdTomato* reporter (Fig. 2 and movies S6 and S7). At the vegetative stage (stage V in Fig. 2), we detected the tdTomato signal throughout P1 and in the L1 and subepidermal layers of the SAM (Figs. 1 and 2, A, B, and F). We determined the developmental stage of the reproductive SAM morphologically and defined three stages (stages R1 to R3; Fig. 2A). At stage R1, the IM elongates longitudinally during the transition to the reproductive phase (Fig. 2A). The tdTomato signal extended in the IM from the L1 to the third and fourth layers (Fig. 2, C and G), although such signals might be partially overestimated due to the stability of the fluorescent proteins. We also detected tdTomato fluorescence in bracts when they formed a small bulge on the side of the IM (Fig. 2, C and G, and movie S6). At stage R2, when PBMs differentiate from the IM (Fig. 2A), the tdTomato signal remained detectable at the outer edge of the IM as in stage R1, although with lower intensity. The tdTomato fluorescence signal formed an intermittent ring surrounding the IM and passed through the bract axil (Fig. 2, D and H, and movie S7). At stage R3, when the PBM develops to form multiple spikelet meristems and/or secondary branches (Fig. 2A), the intensity of tdTomato fluorescence decreased below our detection limit under clearing conditions (Fig. 2E). We therefore repeated our observations at stage R3 without clearing, which allowed us to visualize the tdTomato signal in the region of spikelet meristems and secondary branch meristems (Fig. 2I). Quantification of tdTomato fluorescence revealed that the signal intensity significantly decreased during the reproductive phase (Fig. 2N). These results suggest that the region of active cytokinin signaling extends to the IM during reproductive growth, while the signal intensity weakens.

To investigate the differences in the spatial distribution of cytokinin and auxin signaling in the IM during the reproductive phase, we examined the IMs of the *TCSv2:tdTomato DR5rev:NLS-3xVenus* double transgenic line (Fig. 2, J to M, and movie S8). At stage R2, we detected the Venus signal in the incipient primordia of the bract and three to four cells deep inside this region (Fig. 2, J and K). By contrast, we observed the tdTomato signal in the undifferentiated region of the IM, thus deviating from the distribution of the *DR5-*derived signal (Fig. 2, J and K, and movie S8). Similarly, at stage R3, we detected the Venus signal in the incipient primordia of the bract, while tdTomato fluorescence was concentrated in the region of the secondary branch meristem or spikelet meristem (Fig. 2, L and M). Notably, in the *DII-Venus* line, the fluorescence intensity was weaker at the IM and the tip of the PBM (fig. S3 and movies S9 to S11), suggesting that auxin signaling occurs at the tip of the meristem, which cannot be detected by *DR5rev:NLS-3xVenus*. This difference may reflect a distinction between auxin signaling output and auxin-induced degradation. At the tip of the growing bract, the tdTomato signal surrounded the Venus signal (Fig. 2, J to M). These results suggest that during the reproductive phase, auxin signaling is activated in the region of organ differentiation, while cytokinin signaling is activated in the meristematic region.

We further analyzed the distribution of active auxin and cytokinin signals during bract formation at the reproductive phase (Fig. 2, O to T). Because bracts that form early in the reproductive phase grow larger than those arising later, we focused on early bracts. In the early stage of bract formation (stage b1), the bract primordium formed a small bulge (Fig. 2, O and R); we detected the Venus signal in one to two cells at the tip of the bract (Fig. 2O), while tdTomato fluorescence occupied the entire bract primordium (Fig. 2R). During the second stage of bract formation (stage b2), the bract primordium elongated slightly toward the apical end of the IM. We observed the Venus signal in a stretch of cells at the center of the developing bract, the region presumed to later differentiate into vascular tissues (Fig. 2P). We detected tdTomato fluorescence throughout the bract, as in stage b1 (Fig. 2S). In the late stage of bract formation (stage b3), the bract primordium elongated toward the tip of the IM. We observed the Venus signal in the center of the bract (Fig. 2Q), as in stage b2 (Fig. 2P), while tdTomato fluorescence was restricted to the tip of the bract (Fig. 2T).

### Cytokinins slightly increase Hd3a expression but do not affect FAC-mediated transcriptional activation

**Fig. 3.**
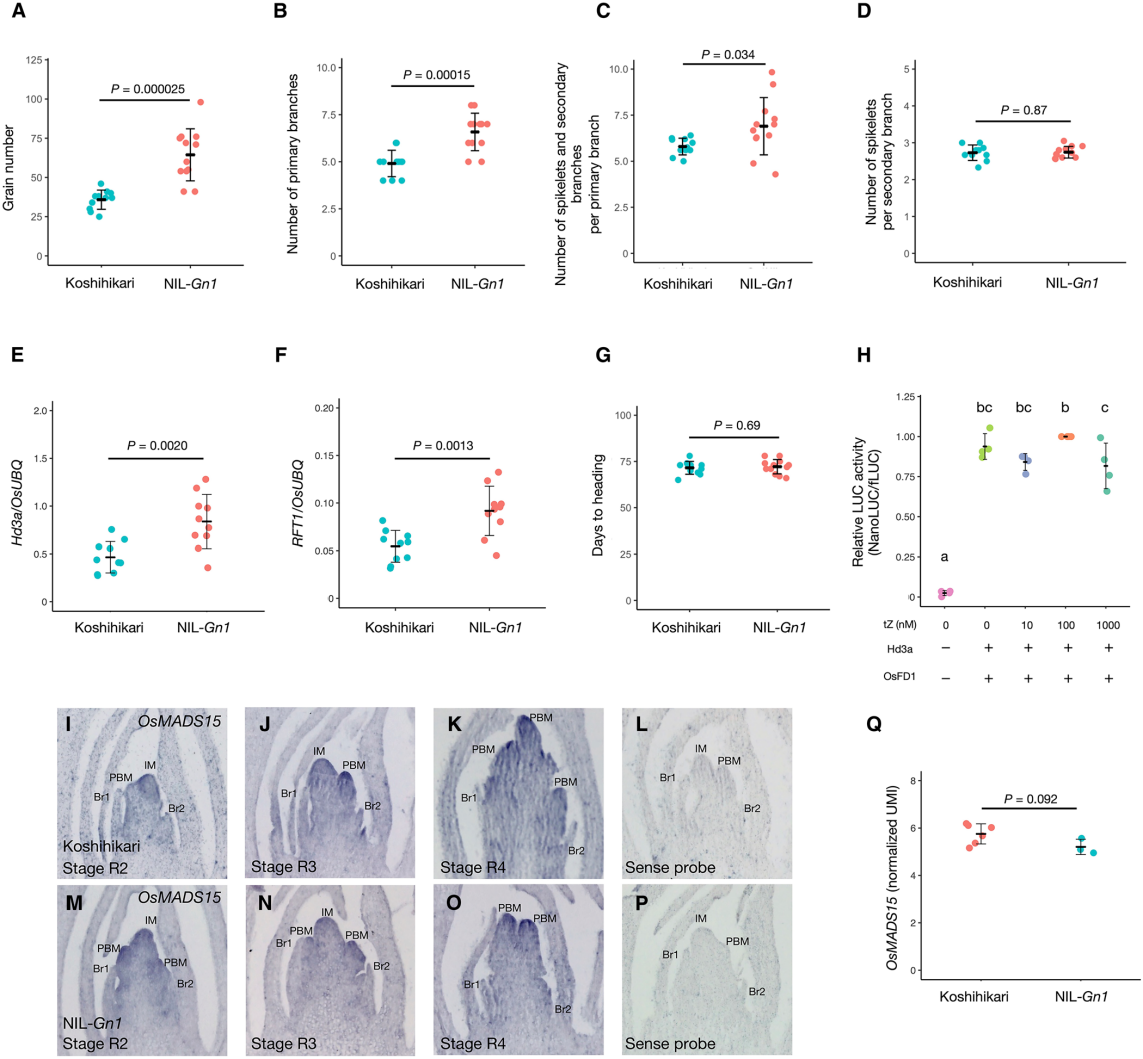
NIL-Gn1 increases the number of inflorescence branches and *Hd3a* and RFT1 expressions but does not affect the expression of OsMADS15 in the SAM. (**A** to **G**) Grain number (A), number of primary branches (B), number of spikelets and secondary branches per primary branch (C), number of spikelets per secondary branch (D), relative *Hd3a* expression levels (E), relative *RFT1* expression levels (F), and days to heading (G) in Koshihikari and NIL-*Gn1*. (**H**) Effect of *trans*-zeatin treatment on the activation of *OsMADS15-NanoLUC* transcription by the FAC. Protoplasts prepared from a cell culture from callus of the *OsMADS15-NanoLUC* knock-in line were transfected with *Hd3a*, *OsFD1*, and the firefly luciferase (*Ubipro*:*fLuc*) reporter and treated with 0, 10, 100, or 1000 nM *trans*-zeatin (tZ) before measuring LUC activity. Protoplast preparation and transfection were performed independently four times (Ex. 1 to 4). Relative LUC activity (NanoLUC/fLuc) at 100 nM tZ was set to 1. (**I** to **P**) In situ hybridization of *OsMADS15* in Koshihikari (I to L) and NIL-*Gn1* (M to P). (**Q**) Normalized unique molecular identifier (UMI) counts of RNA-seq data from Koshihikari and NIL-*Gn1* SAMs. Values are means ± SD (*n* = 11 in A to D and G, *n* = 9 for E and F); the *P* value in [(A) to (G)] was determined by a Student’s *t* test, and different lowercase letters in (H) indicate significant differences (Tukey’s test with α = 0.05).

To explore how cytokinins affect inflorescence development, we characterized the phenotype of a near isogenic line (NIL) for a quantitative trait locus (QTL) associated with increased grain number (NIL-*Gn1*), in which the high-yielding QTL *Grain number 1* (*Gn1*) from rice cultivar Habataki was introgressed into the Koshihikari background (*57*). *Gn1* consists of two sub-QTLs: *Gn1a* and *Gn1b*. Only *Gn1a* has been cloned and was shown to encode a cytokinin oxidase (OsCKX2) that degrades active cytokinins. *Gn1a* in Habataki is a loss-of-function allele, leading to higher cytokinin contents and grain numbers. To assess cytokinin signaling, we introduced the *TCSv2:tdTomato* reporter into NIL-Gn1 and Koshihikari plants and observed their SAMs, confirming stronger cytokinin signaling in NIL-Gn1 (fig. S4). As *Gn1* was reported to affect yield in the field (*57*), we examined the phenotype of NIL-*Gn1* grown under short-day conditions (Fig. 3, A to D). NIL-*Gn1* exhibited higher grain number (Fig. 3A) and more primary branches (Fig. 3B), spikelets, and secondary branches per primary branch (Fig. 3C) compared to Koshihikari. The number of spikelets per secondary branch was comparable between NIL-*Gn1* and Koshihikari (Fig. 3D). These results indicate that cytokinins increase inflorescence branching under short-day conditions, making NIL-*Gn1* an ideal material to explore the connection between cytokinins and inflorescence formation.

As cytokinins and florigen have opposite effects on inflorescence branching (*34*, *57*), we investigated four florigen-related characteristics in NIL-*Gn1* and the control cultivar Koshihikari: *Hd3a* and *RFT1* expression, FAC-mediated transcriptional activation, the distribution of Hd3a in the IM, and the expression of Hd3a target genes. In leaves, the expression of *Hd3a* and *RFT1* was significantly higher in NIL-*Gn1* than in Koshihikari (Fig. 3, E and F), although this up-regulation did not affect days to heading (Fig. 3G). Hd3a activates *OsMADS15* expression by forming a FAC (*29*), prompting us to examine the effects of exogenous cytokinin treatment on the transcriptional activation of *OsMADS15*. Accordingly, we prepared protoplasts from transgenic rice plants harboring *NanoLUC* introduced in-frame into the *OsMADS15* locus by gene targeting (*68*, *69*). In this system, transient expression of *Hd3a* and *OsFD1* induces the formation of a FAC with endogenous 14-3-3 proteins, leading to the activation of *OsMADS15-NanoLUC* transcription. Relative luciferase activity was low in the absence of Hd3a and OsFD1 but high when these two FAC components were introduced. Relative luciferase activity did not change upon treatment with the cytokinin *trans*-zeatin at concentrations of 10 nM to 1 μM (Fig. 3H), indicating that cytokinins do not affect the FAC-mediated transcriptional activation of *OsMADS15*.

To examine whether cytokinin affects *OsMADS15* expression in vivo, we performed in situ hybridization using the shoot apices of Koshihikari and NIL-*Gn1*, including IMs at stages R2 to R4. *OsMADS15* transcripts were detected in the meristematic regions of developing inflorescences, with no discernible difference in staining intensity between the two genotypes (Fig. 3, I to P). Consistently, RNA sequencing (RNA-seq) analysis revealed no significant difference in *OsMADS15* transcript levels between Koshihikari and NIL-*Gn1* (Fig. 3Q; details described below). These results indicate that the elevated cytokinin levels in NIL-*Gn1* do not alter *OsMADS15* expression in the IM, possibly due to redundancy in its regulatory network or compensation due to increased *Hd3a* and *RFT1* expression (Fig. 3, E and F).

### Hd3a and active cytokinin signaling occupy an inverse expression domain at the IM

**Fig. 4.**
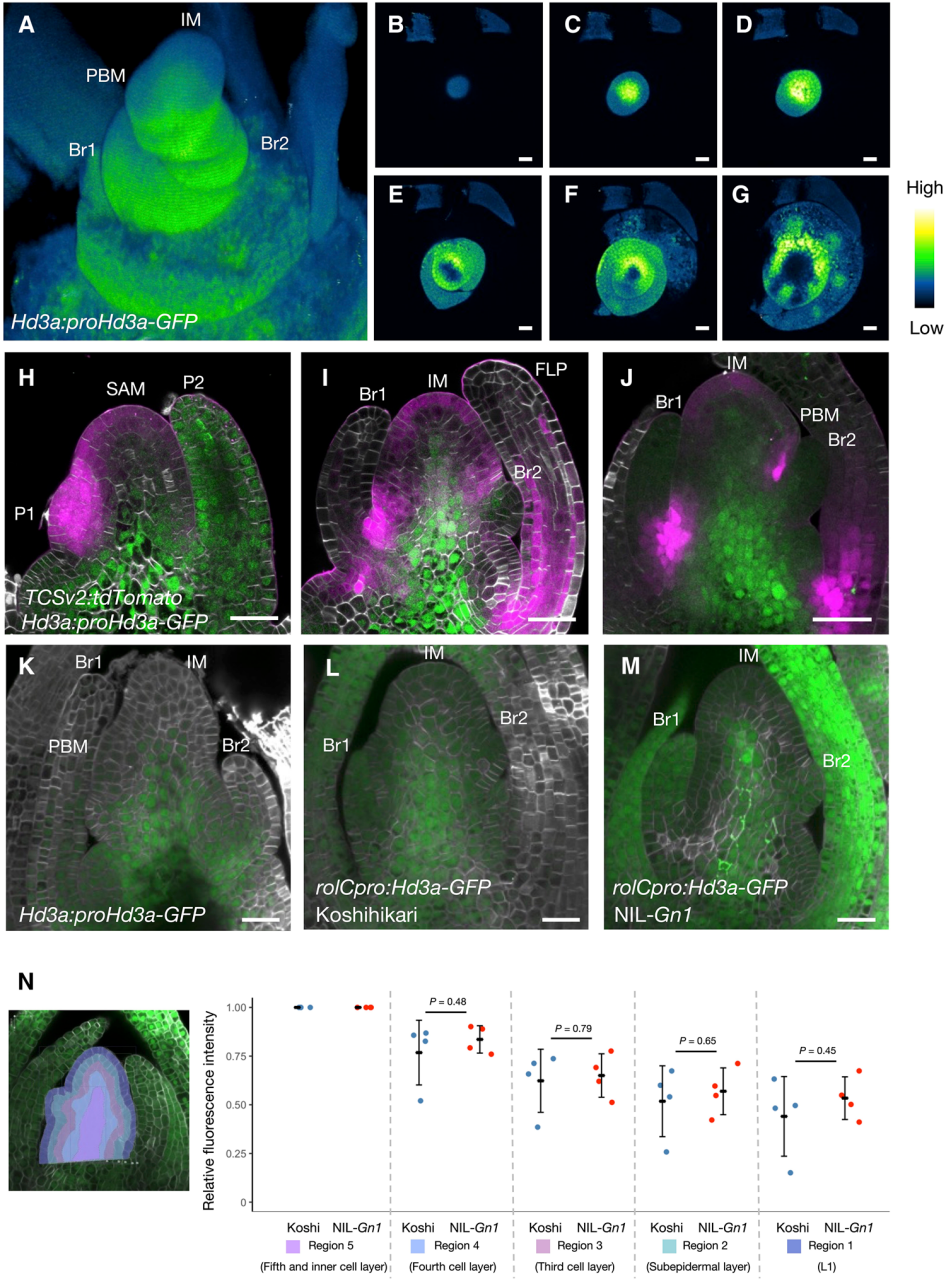
The distribution of Hd3a and active cytokinin signaling forms an inverse expression domain in the IM. (**A** to **G**) Hd3a accumulation pattern in the IM of the *Hd3apro:Hd3a-GFP* transgenic line, obtained by two-photon excitation microscopy. 3D reconstruction (A) and serial cross sections from the top of the IM (B) to the stem just beneath the IM (G). The color bar to the right of (G) indicates GFP fluorescent intensity in (A) to (G). (**H** to **J**) Hd3a accumulation pattern and TCSv2 signals in the SAM and IM of the *Hd3apro:Hd3a-GFP TCSv2:tdTomato* double transgenic line at stage V (H) and stage R2 (I and J). Green and magenta signals indicate GFP from *Hd3apro:Hd3a-GFP* and tdTomato from *TCSv2:tdTomato*, respectively. (**K** to **M**) Hd3a accumulation pattern in the IM of the *Hd3apro:Hd3a-GFP* transgenic line in the Nipponbare background (K) and in the *rolCpro:Hd3a-GFP* transgenic line in the Koshihikari (L) and NIL-*Gn1* (M) backgrounds. (**N**) Quantification of Hd3a distribution in the IMs of Koshihikari and NIL-Gn1. Diagram of the region under analysis showing the different cell layers (left). Relative fluorescence intensity of Hd3a-GFP in each region of the IM in Koshihikari (Koshi) and NIL-Gn1. The fluorescence intensity of region 5 in each IM was set to 1. Values are means ± SD (*n* = 4); *P* values were determined by a Student’s *t* test. IM, inflorescence meristem; Br1, first bract; Br2, second bract; PBM, primary branch meristem. All observations were replicated using more than four independent plants, and representative images are shown. Scale bars, 25 μm.

The function of Hd3a during the IM-to-FM transition is dictated by its spatial distribution in the IM (*34*). We thus observed *Hd3apro:Hd3a-GFP* (*22*), a transgenic line expressing *Hd3a* under the control of the *Hd3a* promoter cloned in-frame with *GFP* to resolve the 3D expression pattern of Hd3a at single-cell resolution by imaging all layers of the IM and uppermost regions of the stem using a two-photon laser microscope (Fig. 4, A to G, and movie S12). On the basis of 3D reconstruction (Fig. 4A) and cross-sectional images of the IM (Fig. 4, B to D), we determined that Hd3a-GFP exhibits a characteristic accumulation domain, with high protein abundance at the center of the IM and lower accumulation at the periphery. In addition, imaging of stem cross sections detected the Hd3a-GFP signal in and around the stem vascular bundles (Fig. 4G and movie S12).

Notably, the distribution of Hd3a (Fig. 4, A to G) and the domain with active cytokinin signaling (Fig. 2) appeared to form an inverse pattern: Hd3a was more abundant at the center of the IM and less abundant at the periphery, while active cytokinin signaling showed the opposite pattern. To directly confirm this pattern, we generated *Hd3apro:Hd3a-GFP TCSv2:tdTomato* double transgenic rice lines and examined their SAMs (Fig. 4, H to J). In these plants, Hd3a-GFP accumulated in the central region of the IM (Fig. 4, I and J), while TCSv2:tdTomato was detected in the peripheral region of the SAM/IM (Fig. 4, H to J), confirming that Hd3a and cytokinin signaling occupy the inner and outer regions of the meristem, respectively.

We hypothesized that active cytokinin signaling may limit the distribution of Hd3a. To test this possibility, we examined the distribution of Hd3a-GFP in the SAM of NIL-*Gn1*. To facilitate observation, we used the *rolC* promoter, a stronger promoter than that of *Hd3a* but with the same vascular bundle expression domain (*22*, *70*–*72*). We introduced the resulting *rolCpro:Hd3a-GFP* construct into Koshihikari and NIL-*Gn1* to assess Hd3a-GFP abundance and localization at the IM. We observed a similar Hd3a-GFP distribution pattern in Koshihikari and NIL-*Gn1* (Fig. 4, K to M). Quantitative analysis across each cell layer failed to detect a difference in Hd3a-GFP abundance between Koshihikari and NIL-*Gn1* (Fig. 4N). In support of this result, there was no significant difference in the expression of *FT-INTERACTING PROTEIN1* (*OsFTIP1*), which regulates the distribution of Hd3a (*73*, *74*), or *OsFTIP10*, which is homologous to *OsFTIP1* and highly expressed in the IM [identified in the public RiceXPro database of microarray data (*75*), https://ricexpro.dna.affrc.go.jp/category-select.php], or *GF14f*, which encodes a 14-3-3 protein that interacts with Hd3a and has the highest expression level among all eight *GF14* genes in the IM (fig. S5) (*29*, *76*). These results indicate that Hd3a and active cytokinin signaling are distributed inside and outside of the IM, respectively, but cytokinins do not modulate the distribution of Hd3a.

### Active cytokinin signaling maintains the IM during the early stage of development

**Fig. 5.**
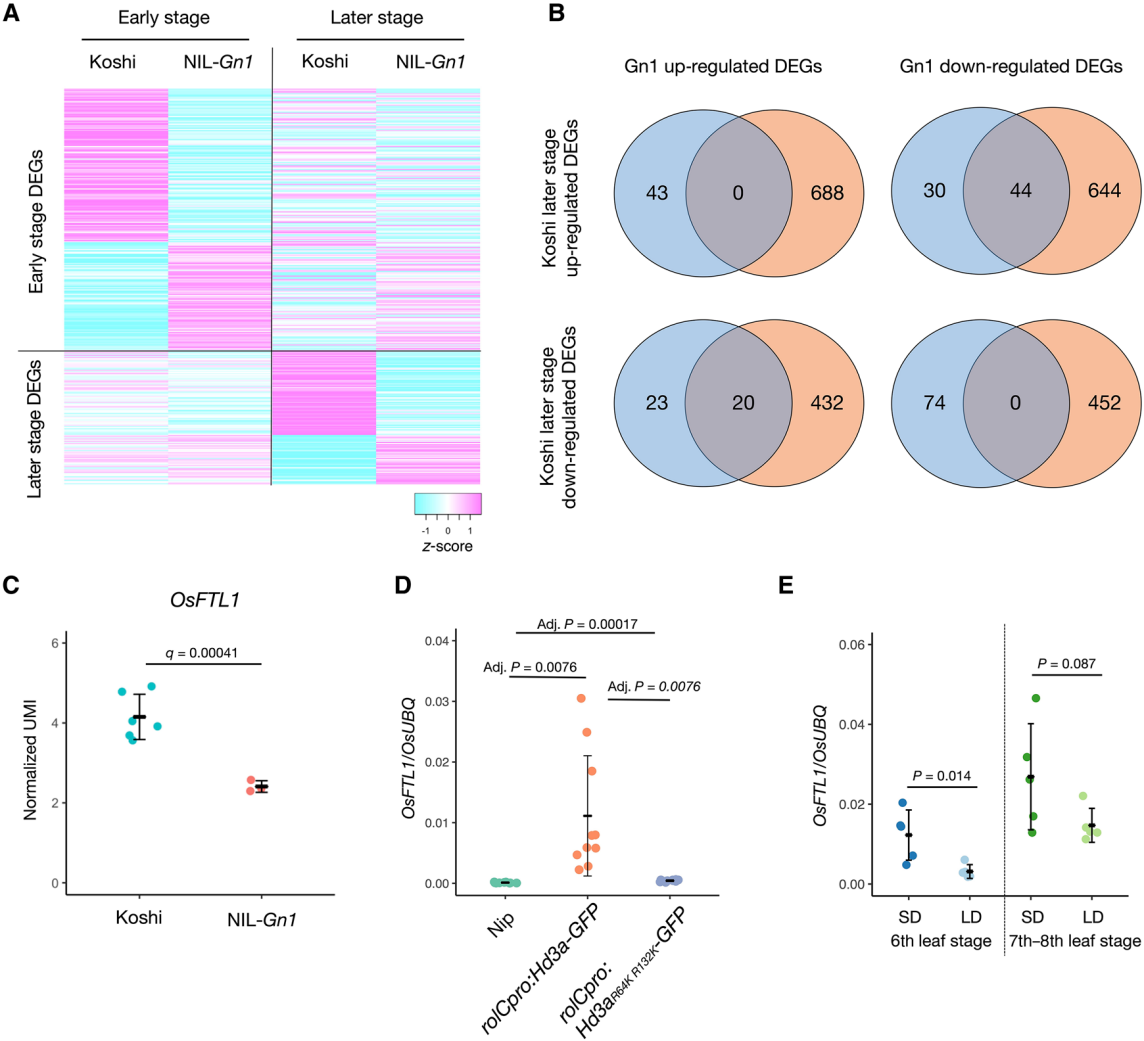
Transcriptome analysis of NIL-*Gn1* identifies *OsFTL1* as a common target gene of cytokinins and Hd3a. (**A**) Heatmap showing the expression levels of differentially expressed genes (DEGs) between Koshihikari (Koshi) and NIL-*Gn1* during early and later stages of inflorescence development. (**B**) Venn diagram showing the extent of overlap between up-regulated or down-regulated DEGs in NIL-*Gn1* and up-regulated or down-regulated DEGs in Koshihikari at the later stage of inflorescence development. (**C**) Normalized UMI counts of *OsFTL1* in Koshihikari and NIL-*Gn1* at the later stage of inflorescence development. (**D**) Relative expression levels of *OsFTL1* in Nipponbare (Nip), *rolCpro:Hd3a-GFP*, as determined by RT-qPCR. (**E**) Relative *OsFTL1* expression levels at the sixth and seventh to eighth leaf stages of Nipponbare grown for 4 days after a shift from short days (SD) to long days (LD). *OsUBQ* was used as an internal reference. Values are means ± SD (*n* = 5 for Koshihikari and *n* = 3 for NIL-Gn1 in C; *n* = 10 for Nipponbare, *n* = 10 for prolC:Hd3a-GFP, and *n* = 10 prolC;Hd3a^R64K, R132K^-GFP in D; *n* = 5 in E); *P* values were determined by a Student’s *t* test.

To identify the genes regulated by Gn1 in the IM, we performed RNA-seq of IMs collected from Koshihikari and NIL-*Gn1* to compare Gn1- and Hd3a-regulated genes and to examine the interaction between Hd3a and active cytokinin signaling in the IM. Accordingly, we collected IMs at the early stage [vegetative-to-reproductive transition (R1) to differentiation of the first one to two PBMs (R2), as shown in Fig. 2] and at a later stage [development of primary branches (R3) to spikelet formation (R5)] of development. We identified 228 differentially expressed genes (DEGs) in the early stage and 117 DEGs in the late stage between Koshihikari and NIL-*Gn1* (Fig. 5A and tables S1 and S2). Gene Ontology (GO) enrichment analysis showed that early-stage DEGs were enriched in the term “Heterocycle biosynthetic process” along with “Cellular developmental processes,” while late-stage DEGs were associated with “mRNA transcription” (fig. S6). We also analyzed the RNA-seq dataset for cytokinin-related genes, auxin-related genes, cell cycle–related genes, and genes regulating meristem function (figs. S7 to S10). We failed to detect significant differences in the expression levels of any of these gene sets between Koshihikari and NIL-*Gn1*, although we did identify several genes that were strongly expressed in the IMs of both genotypes.

As an alternate approach, we attempted to infer the IM states in Koshihikari and NIL-*Gn1* from transcriptome data. We hypothesized that NIL-*Gn1* produces many branches by maintaining the IM in an early state; the underlying genes should thus be highly expressed in NIL-*Gn1* but highly expressed in Koshihikari only at the early stage before being down-regulated at the later stage of IM development. Of the 43 DEGs more highly expressed in NIL-*Gn1* than in Koshihikari at the later stage of development, 20 were more highly expressed at the early stage than at the later stage in Koshihikari (Fig. 5B). By contrast, we identified no DEGs that were more highly expressed in the later stage than in the early stage of IM development in Koshihikari. These results suggest that these 20 genes define a group of highly expressed genes associated with IM branching.

Conversely, we suspected that genes involved in the termination of IM branching would be expressed at low levels in NIL-*Gn1* during both the early and late stages of IM development but would be expressed at low levels at the early stage and be induced at the late stage of IM development in Koshihikari. Of the 74 down-regulated DEGs in NIL-*Gn1* at the later stage of IM development, 44 were up-regulated at the later stage in Koshihikari compared to the early stage (Fig. 5B). None of the 74 down-regulated DEGs in NIL-*Gn1* were down-regulated at the later stage of IM development in Koshihikari relative to the early stage.

### OsFTL1 expression responds to cytokinin and Hd3a activity

We hypothesized that cytokinin and florigen regulate inflorescence development by controlling the expression of common downstream genes. To test this possibility, we looked for overlap between florigen- and cytokinin-regulated genes (fig. S11). Because florigen promotes the vegetative-to-reproductive transition, we selected DEGs in the vegetative-to-reproductive transition at the shoot apex as florigen-regulated genes (*77*), while we selected the DEGs in the later stages of Koshihikari and NIL-*Gn1* as cytokinin-regulated genes. Fifty-eight of these genes overlapped between the two gene lists (fig. S11A). These 58 genes included only 2 genes from a list of 49 genes reported to regulate inflorescence development in the literature (*78*): *OsFTL1* and *TAWAWA1* (*TAW1*) (*58*–*60*, *79*, *80*) (fig. S11B). The other 47 genes regulating inflorescence development did not appear to be differentially expressed during the later stages of IM development in NIL-*Gn1* (fig. S12).

*OsFTL1* encodes an FT-like protein and a paralog of Hd3a that forms FACs and promotes the IM-to-FM transition (*58*, *60*, *81*). Repressing *OsFTL1* expression was reported to increase grain number (*58*, *59*). Notably, *OsFTL1* expression was lower in NIL-*Gn1* compared to Koshihikari (Fig. 5C). *TAW1* encodes a member of the *Arabidopsis* LSH1 and Oryza G1 (ALOG) protein family that harbors a DNA-binding domain and has weak transcriptional activity (*79*). *TAW1* affects the IM-to-FM transition, and lines with increased *TAW1* expression produced more IM branches than the wild type (WT) (*79*, *80*). *TAW1* expression was higher in NIL-*Gn1* than in Koshihikari (fig. S11C). These results suggest that the repression of *OsFTL1* expression and the up-regulation of *TAW1* expression may contribute to the increased grain number observed in NIL-*Gn1*.

To examine whether *OsFTL1* is regulated by Hd3a in a FAC-dependent manner, we examined *OsFTL1* expression in *rolCpro:Hd3a-GFP* transgenic plants*.* The expression of *OsFTL1* was higher in *rolCpro:Hd3a-GFP* transgenic plants compared to the nontransgenic control Nipponbare (Fig. 5D and fig. S13). To assess the requirement for FAC formation for this up-regulation, we examined *OsFTL1* expression in plants accumulating Hd3a carrying a mutation that prevents its interaction with 14-3-3 proteins. Arginine residues 64 and 132 (R64 and R132) of Hd3a are essential for its interaction with 14-3-3 proteins, forming salt bridges with the acidic lobe on the surface of 14-3-3 (*29*). Mutating these residues to lysine prevents Hd3a from interacting with 14-3-3, leading to a failure to form FACs. Transgenic plants carrying the *rolCpro:Hd3a*^*R64K*, *R132K*^*-GFP* transgene failed to show induced *OsFTL1* expression (Fig. 5D and fig. S13), indicating that FAC formation is necessary for *OsFTL1* expression. In addition, we examined *OsFTL1* expression in plants grown under long-day conditions, when *Hd3a* expression is extremely low (*60*). *OsFTL1* expression in the SAM was lower under long-day conditions compared to short-day conditions (Fig. 5E). These results indicate that Hd3a regulates *OsFTL1* expression in the SAM. By contrast, *TAW1* was weakly repressed in *rolCpro:Hd3a-GFP* plants (fig. S11D). These results indicate that *OsFTL1* is a common target gene of cytokinins and Hd3a, making *OsFTL1* a prime candidate that integrates both signaling pathways and regulates inflorescence development.

### OsFTL1 controls flowering and inflorescence architecture by mediating the effects of cytokinin and Hd3a

**Fig. 6.**
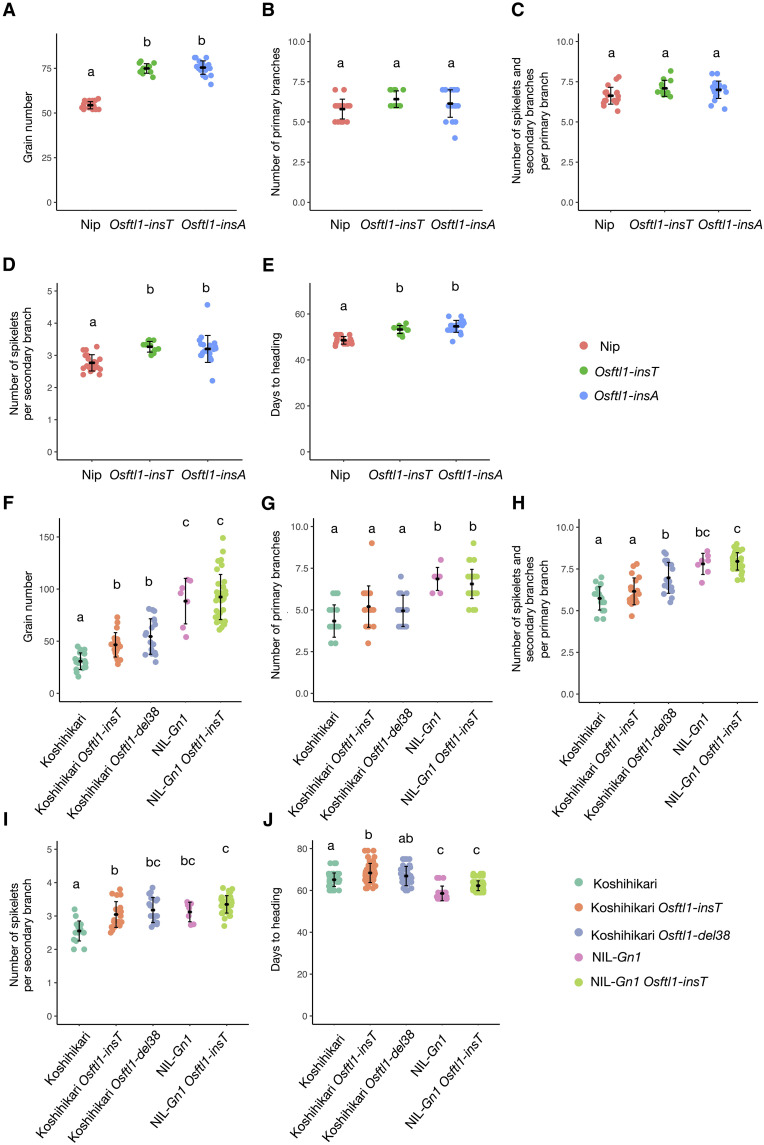
*Gn1* is epistatic to *OsFTL1*, and cytokinins act upstream of OsFTL1. (**A** to **E**) Grain number (A), number of primary branches (B), number of spikelets and secondary branches per primary branch (C), number of spikelets per secondary branch (D), and days to heading (E) in Nipponbare (Nip) and the *Osftl1* mutants. (**F** to **J**) Grain number (F), number of primary branches (G), number of spikelets and secondary branches per primary branch (H), number of spikelets per secondary branch (I), and days to heading (J) in Koshihikari and NIL-*Gn1* with functional OsFTL1 or in the *Osftl1* mutant background. Values are means ± SD [*n* = 16 for Nip, *n* = 12 for *Osftl1-insT*, and *n* = 10 for *Osftl1-insA* in (A) to (E); *n* = 18 for Koshihikari, *n* = 20 for Koshihikari *Osftl1-insT*, *n* = 18 for Koshihikari *Osftl1-del38*, *n* = 7 for NIL-Gn1, and *n* = 35 for NIL-Gn1 *Osftl1-insT* in (F) to (J)]; different lowercase letters indicate significant differences (Tukey’s test, α = 0.05).

To investigate the function of OsFTL1, we generated *Osftl1* loss-of-function mutants by clustered regularly interspaced short palindromic repeats–CRISPR-associated nuclease 9 (CRISPR-Cas9)–mediated genome editing (*82*) targeting exon 1 (fig. S14). We obtained two mutant lines: *Osftl1-insT* and *Osftl1-insA*, carrying a 1-bp frameshift insertion of a T or A in exon 1, respectively. *Osftl1* plants had a higher grain number and spikelets per secondary branch than the Nipponbare parent (Fig. 6, A to D). In addition, the mutants flowered later than Nipponbare (Fig. 6E). These results suggest that *OsFTL1* promotes the floral transition in the SAM and the transition of secondary branch meristems to spikelet meristems. In *Osftl1* mutants, the delay in the latter transitions likely prolongs the period of lateral spikelet initiation, resulting in a significant increase in spikelet number per panicle.

Because *OsFTL1* expression was repressed in *NIL-Gn1* plants, in which cytokinin levels are elevated (Fig. 5C), we investigated the genetic relationship between *OsFTL1* and *Gn1* (Fig. 6, F to J). We generated lines carrying one of two loss-of-function *OsFTL1* mutations in the NIL-*Gn1* background (fig. S14) and examined their inflorescence-related phenotypes. *Osftl1* mutants in the Koshihikari background produced more grains and spikelets per secondary branch and showed delayed flowering compared to the WT (Fig. 6, F to J). NIL-*Gn1* produced more grains compared to Koshihikari, while *Osftl1* NIL-*Gn1* plants did not show increased grain or secondary branch number and did not show delayed flowering compared to NIL-*Gn1* (Fig. 6, F to J). These results suggest that *Gn1* is epistatic to *OsFTL1* for grain number and spikelets per secondary branch. The enhancement of cytokinin levels by *Gn1* appears to mask the effect of *Osftl1*. This interpretation is supported by the reduced expression of *OsFTL1* in NIL-*Gn1* (Fig. 5C), indicating that cytokinin signaling acts upstream to repress *OsFTL1*, thereby regulating these traits. By contrast, the numbers of primary branches and secondary branches per primary branch were unaffected in *Osftl1*, indicating that these aspects of inflorescence architecture are independent of *OsFTL1*.

Because Hd3a induces *OsFTL1* expression, OsFTL1 function would be expected to decline when *Hd3a* expression is repressed under long-day conditions (*60*). *OsFTL1* expression in the SAM was lower under long-day compared to short-day conditions (Fig. 5E). The number of grains, spikelets, secondary branches per primary branch, and spikelets per secondary branch was higher in the *Osftl1* mutant compared to Nipponbare under long-day conditions, but these differences were smaller than under short-day conditions (fig. S15, A to E). These results are consistent with the previous finding that *OsFTL1* expression is reduced in *hd3a* mutants compared to WT plants (*58*). These results indicate that the reduced *Hd3a* expression under long-day conditions also decreased *OsFTL1* expression, resulting in the smaller effects of OsFTL1 on inflorescence phenotypes compared to those observed under short-day conditions.

### OsFTL1 is a mobile protein that regulates inflorescence development

**Fig. 7.**
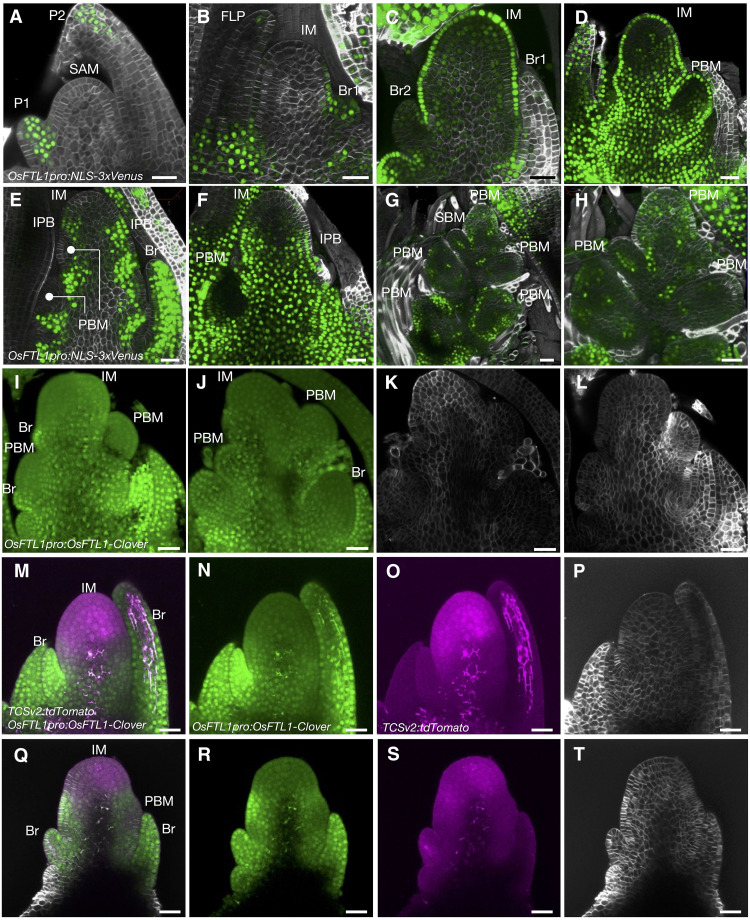
OsFTL1 is a mobile protein that regulates inflorescence development. (**A** to **L**) Distribution of Clover fluorescence in the SAMs and IMs of *OsFTL1pro:NLS-3xVenus* (A to H) and *OsFTL1pro:OsFTL1-Clover* transgenic lines (I to L) at the vegetative stage (A), stage R1 (B), stage R2 (C), stage R3 (D to F and I to L), and stage R4 (G and H). Cell wall staining of the material in (I) and (J) is shown in (K) and (L), respectively. (**M** to **T**) Distribution of Clover (N and R) and tdTomato (O and S) fluorescence in the IMs of *OsFTL1pro:OsFTL1-Clover TCSv2:tdTomato* double transgenic lines at stage R1 (M to P) and stage R2 (Q to T). (M) and (Q) show merged fluorescence images; cell wall staining of the material in (M) and (Q) is shown in (P) and (T), respectively. SAM, shoot apical meristem; P1, youngest leaf primordium (plastochron 1); P2, second youngest leaf primordium (plastochron 2); IM, inflorescence meristem; Br1, first bract; Br2, second bract; Br, bract; PBM, primary branch meristem; IPB, incipient bract. All observations were replicated using more than three independent plants, and representative images are shown. Scale bars, 25 μm.

*OsFTL1* expression was promoted in *rolCpro:Hd3a-GFP* and repressed in NIL-*Gn1*. To investigate the spatial activation of the *OsFTL1* promoter in the IM in more detail, we generated *OsFTL1pro:NLS-3xVenus* transgenic rice plants and visualized the SAM and IM (Fig. 7, A to H, and movies S13 and S14). The promoter used was sufficient to express *OsFTL1* in its proper functional domains, as expressing the *OsFTL1-Clover* fusion construct from this promoter partially rescued the *Osftl1* mutant phenotype (fig. S16). At stage V, we detected *OsFTL1* promoter activity on the adaxial side of P1 and at the tip of P2, but not in the SAM (Fig. 7A). At stage R1, we observed *OsFTL1* promoter activity in the basal region of the flag leaf primordia and at the bract, as well as at the bract-IM boundary and in a few cells of the L1 connected to it (Fig. 7B). At stage R2, *OsFTL1* promoter activity appeared throughout the IM but was more intense in the L1 layer. We also detected Venus fluorescence in bracts (Fig. 7C, fig. S17, and movie S13). At stage R3, *OsFTL1* promoter activity was still detectable throughout the IM, although the signal in the L1 layer of the IM was weaker than at earlier stages [compare stage R2 (Fig. 7C) to R3 (Fig. 7, E and F)]. In the PBM, we observed the Venus signal in L1 (Fig. 7D).

In more highly developed PBMs, such as in stage R3, the Venus signal in the L1 layer of the IM was weak but detectable on the inner side of the IM (Fig. 7, E and F, and movie S13). We detected no signal in the PBM, but we did detect *OsFTL1* promoter activity in developed bracts and in incipient primordia of the bracts (Fig. 7, E and F, and movie S13). The IM aborted at stage R4, preventing any further observation. In the PBM, we observed cells with an activated *OsFTL1* promoter and various Venus fluorescence intensities inside the meristem and in the spikelet meristem and secondary branch meristem (Fig. 7, G and H, and movie S14). These results suggest that *OsFTL1* promoter activity occurs in the bract, the L1 layer, and the inner side of the meristems during stages R2 and R3 and is irregularly distributed along the inner side of the meristem at stage R4. These results are consistent with previous results of RNA in situ hybridization of *OsFTL1* (*58*), especially the weaker signals in the internal region of the IM and tip of the PBM, although there were differences in detailed localizations depending on the method used for observation. This pattern partially overlapped with that of Hd3a distribution and active cytokinin signaling.

Because FT and FT-like proteins are known to undergo cell-to-cell transport (*2*, *22*, *23*), we examined the spatial distribution of OsFTL1 protein in the IM (Fig. 7, I to L). We produced transgenic rice plants expressing *OsFTL1-Clover* under the control of the *OsFTL1* promoter (*OsFTL1pro:OsFTL1-Clover*) and observed the IM. Despite the localized activity of the *OsFTL1* promoter, we detected OsFTL1-Clover throughout the inflorescence, including the IM and PBMs (Fig. 7, I to L), which is consistent with previous observations for the OsFTL1-GFP fusion protein (*58*).

To examine whether OsFTL1 protein diffuses into the cytokinin signaling domain, we generated *OsFTL1pro:OsFTL1-Clover TCSv2:tdTomato* double transgenic rice lines and observed SAMs at different stages (Fig. 7, M to T). At stages R1 and R2, OsFTL1-Clover fluorescence was detected not only in the bract but also in the IM and PBM (Fig. 7, N and R). TCSv2:tdTomato signal was broadly observed at the apical region of the IM (Fig. 7, O and S), and the two signals overlapped within the meristem domain (Fig. 7, M and Q). To test whether cytokinin signaling influences OsFTL1 mobility, we examined whether cytokinin might alter OsFTL1 movement by modulating *FTIP* gene expression, as FTIP can modulate the distribution of FT-like proteins. However, none of the 13 rice *FTIP* genes were differentially expressed between Koshihikari and NIL-*Gn1* SAMs (fig. S12). There is currently no direct evidence that cytokinin signaling regulates the spatial distribution of OsFTL1 in the SAM. However, further studies will be needed to address this question, as cytokinin signaling colocalizes with OsFTL1 in the IM. These observations indicate that OsFTL1 diffuses throughout the inflorescence and is transported from its site of transcription to its site of function, extending into the cytokinin signaling domain at the periphery of the IM.

### OsFTL1 regulates OsMADS15 and development-related gene expression in the SAM

To further investigate the role of *OsFTL1* in the SAM, we performed RNA-seq of WT and *Osftl1* IMs at stages R3 and R4 using 20 biological replicates per genotype (figs. S18 to S25). Across both stages, we identified 1800 DEGs (fig. S18). These DEGs were further analyzed for their overlap with cytokinin-related genes (fig. S19), auxin-related genes (fig. S20), cell division–related genes (fig. S21), flowering-related genes (fig. S22), and meristem activity–related genes (fig. S23). We found that promotive floral regulators, including *OsMADS15* and other class A MADS-box genes, were down-regulated in *Osftl1*, while repressive genes such as *TAW1* and *OsSPL14* showed increased expression in the mutants (figs. S22 and S24). These changes are consistent with a delay in the floral transition and an increase in yield potential, supporting a promotive role for *OsFTL1* in the SAM. Notably, the *Osftl1* mutants exhibited reduced expression of the indeterminacy-maintaining gene *OSH1* and increased expression of the CLV1 homologs *FON1* and *FOL2*, suggesting a shift toward differentiation (figs. S23 and S24). GO enrichment analysis revealed stage-specific patterns: Gene silencing–related terms were enriched at R3, whereas translation- and ribosome-related terms were enriched at R4 (fig. S18, B to E). A comparison of *Osftl1* DEGs with Hd3a-regulated DEGs from vegetative and reproductive shoot apices (*77*) identified overlapping genes enriched for developmental and gene silencing–related GO terms (fig. S25). These results indicate that both Hd3a and OsFTL1 contribute to gene silencing in the SAM, which is consistent with previous reports of increased genome-wide DNA methylation during phase transitions in rice (*76*).

**Fig. 8.**
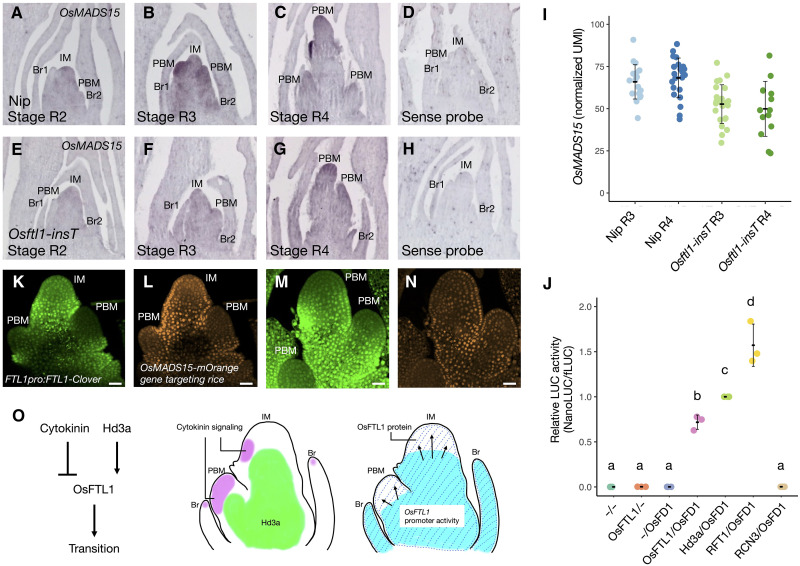
OsFTL1 activates *OsMADS15* expression in vivo. (**A** to **H**) In situ hybridization of *OsMADS15* of the IM in Nip (A to D) and Osftl1-insT (E to H) at stage R2 (A and E), stage R3 (B and F), and stage R4 (C and G), and in situ hybridization of the sense probe (D and H). (**I**) Normalized UMI counts of *OsMADS15* in Nip and *Osftl1-insT* at stages R3 and R4. (**J**) Activation of *OsMADS15pro:OsMADS15-NanoLUC* transcription by OsFTL1. Protoplasts prepared from *OsMADS15-NanoLUC* knock-in cells were transfected with constructs harboring *Hd3a*, *RFT1*, *OsFTL1*, *RCN3*, *OsFD1*, and *Ubipro*:*fLuc*. Protoplast preparation and transfection were performed independently three times. The relative LUC activity (NanoLUC/fLuc) of samples transfected with *Hd3a* and *OsFD1* was set to 1. Values are means ± SD (*n* = 3). (**K** to **N**) Distribution of Clover (K and M) and mOrange (L and N) fluorescence in the IM and the PBM of the *OsFTL1pro:OsFTL1-Clover OsMADS15-mOrange gene targeting* double transgenic line at stage R3. (**O**) Proposed model of the spatial integration of the florigen Hd3a and cytokinin signaling by OsFTL1 in the rice IM. Cytokinin signaling represses and florigen induces *OsFTL1* expression (left). Hd3a (green) accumulates in the central region of the IM, whereas cytokinin signaling (magenta) is active in the primary branch meristems formed at the periphery of the IM (center). OsFTL1 is present throughout the inflorescence (shaded area), where the protein is transported from the site of *OsFTL1* promoter activity (blue) to its site of function (right). IM, inflorescence meristem; PBM, primary branch meristem. All observations were replicated using more than three independent plants, and representative images are shown. Scale bars, 25 μm.

To examine whether OsFTL1 regulates *OsMADS15* expression in vivo, we performed in situ hybridization using shoot apices from WT and *Osftl1* plants. The spatial expression domain of *OsMADS15* was largely unchanged between the two genotypes; however, staining intensity was consistently reduced in *Osftl1* across stages R3 and R4 (Fig. 8, A to H). Although in situ hybridization is not fully quantitative, the consistent reduction in signal suggests that OsFTL1 positively regulates *OsMADS15* expression in vivo. Consistent with this observation, RNA-seq of WT and *Osftl1* SAM samples (Fig. 8I) also showed a reduction in *OsMADS15* transcript levels in the *Osftl1* mutant.

Our observations suggest that OsFTL1 regulates the expression of the FAC target gene *OsMADS15* in the IM and PBM (*29*). To test this hypothesis, we performed transient assays to determine whether OsFTL1 would form FACs and activate *OsMADS15* expression using protoplasts from the suspension cultured cells derived from OsMADS15:nanoLuc gene targeting rice plants (*68*). As shown in Fig. 7J, coexpressing *Hd3a* or *RICE FLOWERING LOCUS T* (*RFT1*) with *OsFD1* induced the transcription of *OsMADS15*, whereas coexpressing *RICE CENTRORADIALIS 3* (*RCN3*; an antiflorigen and an ortholog of *Arabidopsis TERMINAL FLOWER1*) with *OsFD1* did not, validating the assay conditions (*29*, *35*). When we tested OsFTL1 function in this assay, coexpressing *OsFTL1* and *OsFD1* induced *OsMADS15* transcription (Fig. 8J), suggesting that OsFTL1 forms a FAC that activates transcription from the *OsMADS15* promoter.

To assess whether the localization of OsFTL1 and the expression domain of *OsMADS15* spatially overlap in the inflorescence, we examined the IMs of double transgenic rice plants with *OsFTL1pro:FTL1-Clover* and *OsMADS15-mOrange gene targeting*, the latter line harboring an in-frame knock-in insertion of *mOrange*, encoding a fluorescent protein that can be used to visualize the expression domain of OsMADS15 (*34*) (Fig. 8, K to N). At stage R2, OsFTL1 and OsMADS15 colocalized in the IM based on the detected Clover and mOrange fluorescence patterns, respectively (Fig. 8, K and L). At stage R3, OsFTL1 and OsMADS15 colocalized in the PBM, although the signal was weak (Fig. 8, M and N). By contrast, Hd3a-GFP accumulated at the center of the IM, which did not sufficiently cover the entire accumulation domain of OsMADS15, as shown by observing the IM in the *Hd3apro:Hd3a-GFP OsMADS15-mOrange gene targeting* double transgenic line (fig. S26). These data suggest that the localization of OsFTL1 and the expression of *OsMADS15* spatially overlap in the inflorescence. On the basis of these observations, we conclude that OsFTL1 spreads throughout the IM and forms a FAC to activate *OsMADS15* transcription.

## DISCUSSION

In this study, we showed that OsFTL1 integrates developmental signals from cytokinins and florigen in the rice IM (Fig. 7O). We showed that Hd3a and cytokinin signaling form opposite expression domains in the IM. We identified *OsFTL1* as a common regulatory target of florigen and cytokinins: Florigen and cytokinins are antagonistic regulators of *OsFTL1* expression, with florigen inducing and cytokinin repressing *OsFTL1* expression in the IM. The *OsFTL1* promoter is active in the region where Hd3a accumulates but cytokinin signaling is low, whereas OsFTL1 protein spreads beyond the region where Hd3a is distributed, throughout the IM, overlapping with the *OsMADS15* expression domain (Fig. 8O and fig. S27).

### Spatial distribution of active auxin and cytokinin signaling during lateral organ differentiation

In this study, we revealed the changes in the spatial distribution of active cytokinin and auxin signaling in the rice IM in detail. Active auxin signaling consistently localized to regions of organ differentiation, such as leaf primordia and bracts (Figs. 1 and 2 and figs. S1 to S3), supporting the previous finding that localized auxin signaling determines the site of organ differentiation (*42*–*44*). Active zones of cytokinin signaling surrounded those of auxin signaling in growing lateral organs (Figs. 1 and 2). Considering that cytokinins promote cell division (*50*), cytokinin signaling may promote cell division around regions of organ differentiation induced by active auxin signaling during organogenesis. Active cytokinin signaling covered the regions with active auxin signaling for growing lateral organs, such as leaf primordia during the vegetative phase and elongating bracts formed at the early stage of the reproductive phase (Figs. 1 and 2, O to T). By contrast, when the suppression of bract development was established after stage R3, we observed active auxin signaling at cryptic bracts that did not grow (Fig. 2). We also failed to detect active cytokinin signaling in these regions, suggesting a role for cytokinins in organ growth (*50*, *51*).

### Cytokinins regulate developmental phase transitions

The relationship between cytokinins and developmental phase transitions is not well understood. In this study, we established that cytokinins delay developmental phase transitions in rice inflorescences. In NIL-*Gn1*, cytokinin levels are higher, as is grain number, owing to greater inflorescence branching caused by a delay in the transition from the IM to FM, although the underlying mechanism has been unclear. In this study, we found how cytokinins regulate this transition. Cytokinin signaling is activated at the periphery of the meristem, where the transition from the IM to FM occurs; the intensity of active cytokinin signaling weakens in the IM as the reproductive phase progresses (Fig. 2). The early transcriptional state observed in Koshihikari was maintained in NIL-*Gn1* at the later stage, in agreement with the rise in branch number in this NIL (Fig. 5B). In NIL-*Gn1*, the expression of *OsFTL1*, which promotes the transition of the IM to FM, was suppressed (Figs. 5C and 6). These results suggest that cytokinins spatially regulate the progression of the IM-to-FM transition during the reproductive phase. Cytokinins were previously shown to promote the vegetative-to-reproductive transition in *Arabidopsis* by inducing the expression of the *FT* homolog *TWIN SISTER OF FT* in leaves (*83*). Our study showed that cytokinin-induced developmental phase transitions also take place during the IM-to-FM transition in rice inflorescences. Notably, while cytokinins have opposite effects on the vegetative-to-reproductive transition in *Arabidopsis* and the IM-to-FM transition in rice, *FT*-like genes are regulated by cytokinins in both cases.

### Hd3a and cytokinin signaling regulate OsFTL1 expression

Our results indicate that Hd3a promotes *OsFTL1* expression in the rice IM, whereas cytokinin signaling represses its expression (Fig. 8O), although the precise molecular mechanisms and direct regulators remain to be fully elucidated. Several lines of evidence suggest that Hd3a directly regulates *OsFTL1* expression through the formation of a FAC. A previous study demonstrated direct binding of Hd3a to the *OsFTL1* promoter via chromatin immunoprecipitation assays using a commercially available anti-FT antibody (*58*). Consistent with this model, we observed that LD conditions that suppress *Hd3a* expression reduced *OsFTL1* expression, whereas its activation enhanced *OsFTL1* expression (Figs. 5, D and E, and 8J). This induction was abolished when Hd3a was unable to interact with 14-3-3 proteins, pointing to the requirement for FAC formation for *OsFTL1* regulation (Fig. 5D).

Conversely, cytokinin is thought to repress *OsFTL1* expression via a type B RR to OsSPL14 regulatory cascade. In rice inflorescences, cytokinin signaling through the OHK4 receptor activates type B response regulators such as OsRR21, which, in turn, induce *OsSPL14* transcription (*84*). OsSPL14 directly binds to the *OsFTL1* promoter (*85*) and represses its expression (*58*), thereby linking cytokinin signaling to *OsFTL1* suppression. However, in our RNA-seq analysis of NIL-*Gn1* SAMs, neither *type B RRs* nor *OsSPL14* were identified as DEGs (tables S1 and S2), likely reflecting transient or spatially restricted activation not captured under our conditions. Furthermore, another reported activator of *OsFTL1*, *OsDREB1C* (*86*), was also not detected as a DEG in the SAM. These findings indicate that the proposed OsRR21–OsSPL14 pathway has yet to be experimentally validated in the SAM context. While our data clearly demonstrate that cytokinin suppresses *OsFTL1* expression, the precise transcriptional regulators involved remain unresolved, highlighting the need for further mechanistic studies.

Because of the use of cleared tissue and high-resolution imaging, our study achieved higher spatial and temporal resolution of OsFTL1 activity compared to previous work (*58*). Using a transcriptional reporter, we detected *OsFTL1* promoter activity in the IM immediately after the vegetative-to-reproductive transition (Fig. 7, A to H, and movie S13), where activity first appeared in the differentiated bract 1, transiently occurred in the L1 layer, and disappeared as development progressed; in the PBM, promoter activity was extremely low. Moreover, our cleared-tissue imaging revealed OsFTL1 protein in both the IM and PBM (Figs. 7 and 8). Furthermore, dual-reporter assays using *OsFTL1pro:OsFTL1*-*Clover* with *TCSv2:tdTomato* (Fig. 7, M to T) and with *OsMADS15:mOrange* (Fig. 8, K to N) demonstrated that OsFTL1 protein overlapped with the cytokinin signaling and *OsMADS15* expression domains, respectively.

### OsFTL1 expression integrates cytokinin and florigen signaling in the IM

We showed that the activation of *OsFTL1* expression integrates cytokinin signaling with florigen function in the IM. Increased cytokinin signaling in the SAM of NIL-*Gn1* repressed the expression of *OsFTL1*, while Hd3a increased *OsFTL1* transcription by forming a FAC (Figs. 5, 7, and 8). These opposite regulatory signaling branches are spatially coordinated within the IM, as we detected *OsFTL1* promoter activity both in the region where active cytokinin signaling occurs and in the region where Hd3a accumulates (Figs. 2, 4, and 7). OsFTL1 diffused over a wider region than that defined by *OsFTL1* promoter activity (Fig. 7), suggesting that OsFTL1 protein may transmit information about the balance between cytokinin and Hd3a signals throughout the meristem. The FT-like proteins Hd3a and OsFTL1 may thus achieve such regulation through a “florigen relay” within rice IMs: Hd3a forms a distribution gradient with a concentration that decreases from the center to the outer layers of the IM (Fig. 4), activating *OsFTL1* expression as a function of its distance from the IM center. OsFTL1 protein is then transported to the periphery of the IM, thereby activating FAC target genes such as *OsMADS15* throughout the inflorescence (Fig. 8).

A previous study indicated that OsFTL1 is not transported from the leaf to the SAM over long distances and suggested that OsFTL1 is not transported over short distances within the SAM (*58*). These findings differ from the current observations. The difference in short-distance transport may be due to the sensitivity of the reporters used: We used a nucleus-localized promoter activity reporter with single-cell resolution 3D imaging (Fig. 7). In our observations, promoter activity localized to the central region of the IM and bracts (Fig. 7, E and F), which is consistent with previous RNA in situ hybridization results (*58*), whereas the OsFTL1 protein signal was detected throughout the IM (Figs. 7, I to T, and 8, K and L). In addition, the OsFTL1 accumulation domain spatially overlapped with the distribution of the fluorescence from the OsMADS15-mOrange reporter (Fig. 8, K to N), whose associated transcription is activated by OsFTL1 in a FAC-dependent manner (Figs. 5D and 8J). Future research may clarify the mobility of OsFTL1. We propose that *OsFTL1* integrates multiple signals. For example, in addition to the results presented here, the *OsFTL1* promoter was shown to be activated by OsSPL14 in bracts (*58*, *77*, *85*). *OsSPL14* is expressed in bracts, and the transcription factor OsSPL14 binds directly to the *OsFTL1* promoter, which is consistent with the lower *OsFTL1* expression observed in the *spl14 spl17* mutant (*58*). In light of the regulation of *OsFTL1* expression by Gn1 and Hd3a revealed in this study, we suggest that OsFTL1 integrates signals from multiple developmental regulators with different spatial distributions to modulate developmental transitions of the entire inflorescence.

## MATERIALS AND METHODS

### Plant materials and growth conditions

*Japonica* rice (*O. sativa*) cultivar Nipponbare was used to generate the *TCSv2:tdTomato* (*61*, *65*), *Hd3apro:Hd3a-GFP* (*22*), *rolCpro:Hd3a-GFP* (*22*), *rolCpro:Hd3a*^*R64K*,*R132K*^*-GFP* (*29*), *OsFTL1pro:NLS-3xVenus*, *OsFTL1pro:OsFTL1-Clover*, *TCSv2:tdTomato Hd3apro:Hd3a-GFP*, *TCSv2:tdTomato OsFTL1pro:OsFTL1-Clover*, *OsFTL1pro:OsFTL1-Clover OsMADS15-mOrange gene targeting*, and *Hd3apro:Hd3a-GFP OsMADS15-mOrange gene targeting* transgenic lines. The *OsMADS15-mOrange gene targeting* line was described previously (*34*). *Japonica* rice cultivar Norin 8 was used to generate the *DR5rev:NLS-3xVenus* (*64*) and *DII-Venus* (*67*) transgenic lines. *Japonica* rice cultivar Koshihikari and NIL-*Gn1* (*57*) were used to generate *rolCpro:Hd3a-GFP*. All plants were grown in climate chambers with 70% relative humidity under short-day conditions consisting of daily cycles of 10 hours of light at 28°C and 14 hours of darkness at 25°C. Light was provided by fluorescent white light tubes (400 to 700 nm, 100 μmol m^−2^ s^−1^). *DR5rev:NLS-3xVenus* and *TCSv2:tdTomato* double transgenic plants were generated by crossing the parental lines. Plants were transformed as previously described (*87*, *88*).

### Plasmid construction

The *TCSv2:tdTomato* construct was obtained using a pENTR plasmid containing the *TCSv2:tdTomato* sequence amplified from *TCSv2:tdTomato* in pTF101.1gw as a template (*61*, *65*). To produce the *OsFTL1pro:NLS-3xVenus* and *OsFTL1pro:OsFTL1-Clover* transformation vectors, the 3.0-kb promoter fragment upstream of the *OsFTL1* translation initiation codon was amplified by polymerase chain reaction (PCR) using genomic DNA from Nipponbare as a template. The amplified fragment was cloned into pENTR-D-TOPO to generate pENTR-OsFTL1pro. pENTR-OsFTL1pro was digested with Xba I to release the Os*FTL1pro* fragment with Xba I ends. This fragment was introduced into the Xba I site upstream of the ccdB cassette in pGWB525 (*89*) to produce pHT110. The full-length coding sequence of *OsFTL1* was amplified by reverse transcription–PCR using RNA extracted from the rice SAM as a template and cloned into pENTR-D-TOPO to generate pENTR-OsFTL1. The *OsFTL1pro:NLS-3xVenus* and *OsFTL1pro:OsFTL1-Clover* transformation vectors were constructed via Gateway LR reaction between pENTR:NLS-3xVenus or pENTR:OsFTL1-Clover and pHT110. The *DII-Venus* vector was generated using the pENTR plasmid containing an amplified *DII-Venus* sequence using pH7m34GW as a template (*67*). The *Ubipro:DII-Venus* transformation vector was constructed by Gateway LR reaction between pENTR:DII-Venus and p2K-GW [a binary vector for transgenic plants expressing genes under the control of the maize *Ubiquitin* (*Ubi*) promoter] (*29*). To produce the *Osftl1* mutant using CRISPR-Cas9, a single guide RNA targeting the sequence 5′-GGAGCGCGGGAGGTGGCCAA-3′ was designed manually, cloned into pU6gRNA, and introduced into pZDgRNA_Cas9ver.2_HTP (*82*, *90*, *91*). All primers used for plasmid construction are listed in table S3.

### Tissue clearing

To collect shoot apices, several leaves were carefully removed from the basal part of the plant by hand sectioning to expose the P2 leaf primordium under a stereomicroscope. The shoot apex was excised and fixed in a microtube. The samples were fixed in 4% (w/v) formaldehyde in phosphate-buffered saline (PBS) with 0.1% (v/v) SCRI Renaissance Stain 2200 (SR2200, cell wall stain; the concentration of the solution from the supplier was considered to be 100%; Renaissance Chemicals, UK). Samples were infiltrated in the fixative under a vacuum for 10 min on ice, followed by incubation for 50 min at room temperature under normal pressure. The samples were transferred to destaining solution [100 mM sodium phosphate buffer, pH 8.0, with 20% (w/v) caprylyl sulfobetaine (TCI, Japan)] and incubated for 24 hours at room temperature. The samples were then cleared in iTOMEI (*92*–*94*), transferred to clearing solution [56.2% (w/w) Histodenz (Merck, USA) in PBS], and incubated for 1 hour at room temperature. The samples were mounted in clearing solution on glass slides.

### Imaging by confocal laser scanning microscopy

The samples were visualized using a confocal laser scanning microscope (TCS SP8; Leica Microsystems, Tokyo, Japan) equipped with a 405-nm pulsed white light laser (WLL) source and a 63× glycerol-immersion objective lens (HC PL APO 63×/1.30 GLYC CORR CS2; Leica Microsystems). For SR2200 fluorescence, images were captured at 410 to 480 nm after excitation at 405 nm with a solid-state laser. For GFP and Clover fluorescence, images were captured at 495 to 600 nm after excitation at 490 nm with a WLL. For Venus fluorescence, images were captured at 520 to 600 nm after excitation at 515 nm with a WLL. For tdTomato fluorescence, images were captured at 548 to 700 nm after excitation at 543 nm with a WLL. The Z-step was set to 500 nm for SAMs. The images were processed using LASX software (Leica Microsystems, Tokyo, Japan). WT root tips and IMs from *Hd3apro:Hd3a-GFP OsMADS15-mOrange gene targeting* double transgenic plants were visualized under a confocal laser scanning microscope (LSM880; Carl Zeiss, Oberkochen, Germany).

To quantify *DR5rev:NLS-3xVenus* and *TCSv2:tdTomato* signals in root tips treated with cytokinin or auxin, fluorescence was measured in the root tip and epidermis across four to six replicate images. Background fluorescence from the endodermis was subtracted, and the resulting values were used as normalized intensities. To quantify *TCSv2:tdTomato* in the SAM, fluorescence intensity was calculated as the difference between the signal region (the two apical cell layers where reporter expression is localized) and the background region (the central basal area lacking signal).

### Imaging using two-photon excitation microscopy

Fluorescence images of IMs expressing *Hd3apro:Hd3a-GFP* were obtained with an upright two-photon excitation microscope (FVMPE-RS; Olympus) equipped with dual femtosecond pulse lasers (InSight DeepSee Dual-OL; Spectra-Physics, CA, USA), a 25× 1.00 numerical aperture (NA) objective (XLSLPLN25XGMP; Olympus), and a 10× 0.6 NA objective (XLPLN10XSVMP; Olympus). The excitation wavelength for GFP was 920 nm. Image processing was performed with ImageJ (*91*).

### RNA extraction and RT-qPCR

Total RNA was extracted from the indicated samples using a Plant Total RNA Mini Kit (Favorgen, Pingtung, Taiwan) and treated with DNase I (Thermo Fisher Scientific, Waltham, MA, USA). First-strand cDNA was synthesized using SuperScript IV reverse transcriptase (Thermo Fisher Scientific) and used for quantitative analysis of gene expression, which was performed using SYBR Green PCR Master Mix (Thermo Fisher Scientific). The data were collected using a StepOne Plus sequence detection system (Thermo Fisher Scientific) and a Thermal Cycler Dice Real Time System III (TaKaRa) in accordance with the manufacturers’ instructions. All primers used for quantitative PCR (qPCR) are listed in table S3. All quantification is based on the standard curves for all genes, with *OsUBQ* used as the reference transcript (*22*).

### RNA-seq and data analysis

IMs at stages R2 to R5 were collected from Koshihikari and NIL-*Gn1* plants. IMs at stages R3 and R4 were collected from Nipponbare and *Osftl1* plants. Total RNA was extracted from individual IMs using a Plant Total RNA Mini Kit (Favorgen). First-strand cDNA was synthesized using SuperScript IV Reverse Transcriptase (Thermo Fisher Scientific). RNA-seq data were obtained according to the CEL-Seq2 (*95*) method with some modifications. Briefly, 20 pg of RNA was used for amplification by in vitro transcription. Libraries were amplified with 11 to 15 cycles of PCR, which was empirically determined. The libraries were sequenced on Illumina platforms. GO enrichment analysis was conducted with Shiny GO 0.82 (*96*). To identify DEGs, genes that were highly expressed in only one sample were excluded. For this purpose, genes with a ratio < 0.7 (Koshihikari and NIL-*Gn1*) and 0.15 (Nipponbare and *Osftl1*) between the unique molecular identifier (UMI) count of the sample with the highest UMI count and the sum of UMI counts from all other samples were removed from analysis. DEGs were detected by the Tag Count Comparison method with *q* value < 0.05 (*97*). To generate expression plots and heatmaps, normalized UMI values were calculated from raw UMI counts, assuming that the total UMI for each sample was 100,000.

### Transient assay in protoplasts

Cell culture, protoplast preparation, and transfection were performed essentially as previously described (*68*). Briefly, suspension cells from callus of the *OsMADS15-NanoLUC* knock-in line, which expresses a fusion protein of OsMADS15 and NanoLuc, were cultured in R2S medium with shaking at 100 rpm at 30°C in the dark (*68*). The cells were treated with cellulase solution [Cellulase Onozuka RS (40 mg ml^−1^; Yakult Pharmaceutical Industry), Macerozyme R-10 (10 mg ml^−1^; Yakult Pharmaceutical Industry), 0.5 M sucrose, 10 mM CaCl_2_, MES-KOH (1 mg ml^−1^), and bovine serum albumin (1 mg ml^−1^), pH 5.6] to prepare protoplasts. Approximately 0.1 × 10^6^ protoplasts were transfected with 0.5 μg of *Ubipro:Hd3a*, *Ubipro:RFT1*, *Ubipro:OsFTL1*, or *Ubipro:RCN3* plasmid; 0.5 μg of *Ubipro:FD1* plasmid; and 0.5 μg of *Ubipro:Fluc* plasmid. The transfected protoplasts were resuspended and incubated in various concentrations of *trans*-zeatin at 30°C in the dark for 16 hours. Nluc and firefly luciferase (Fluc) activities in the lysate were measured separately before calculating their ratio (*68*). The Nano-Glo Luciferase Assay System and Dual-Luciferase Reporter Assay System were used according to the manufacturer’s instructions.

### In situ hybridization

In situ hybridization was performed using antisense RNA probes. To synthesize the probes, cDNA fragments from *OsMADS15* were amplified and cloned using a Zero-Blunt TOPO PCR Cloning Kit (Thermo Fisher Scientific, Waltham, MA, USA). The cDNA plasmids were used as templates, and antisense RNA probes were synthesized using a DIG RNA Labeling Kit (Roche Diagnostics GmbH, Mannheim, Germany). The samples were fixed in 4% (w/v) paraformaldehyde and 1% Triton X-100 in PBS overnight at 4°C. The samples were dehydrated using a graded ethanol series, immersed in Histo-Clear (National Diagnostics, Atlanta, GA, USA), and embedded in Paraplast Plus (McCormick Scientific, Berkeley, MO, USA). The samples were cut into 8-μm-thick sections using a rotary microtome, and longitudinal and cross sections containing SAM tissue were prepared. Hybridization and chromogenic reactions were performed as previously described (*98*).
