# Supplementary material for: Florigen and cytokinin signaling antagonistically regulate FLOWERING LOCUS T-LIKE1 to drive a florigen relay that facilitates inflorescence development in rice
Source: Sci Adv. 2025 Dec 19;11(51):eadv1424. doi: 10.1126/sciadv.adv1424 (PMC12716427; doi:10.1126/sciadv.adv1424)
Supplement: Supplementary file 1 — Figs. S1 to S27 Legends for tables S1 to S5 Legends for movies S1 to S14 [file sciadv.adv1424_sm.pdf]

Science Advances
AAAS

Supplementary Materials for

Florigen and cytokinin signaling antagonistically regulate FLOWERING LOCUS T-LIKE1 to drive a florigen relay that facilitates inflorescence development in rice

Moeko Sato *et al.*

Corresponding author: Hiroyuki Tsuji, tsujih@agr.nagoya-u.ac.jp, tsujih@yokohama-cu.ac.jp

Sci. Adv. **11**, eadv1424 (2025)
DOI: 10.1126/sciadv.adv1424

The PDF file includes:

Figs. S1 to S27
Legends for tables S1 to S5
Legends for movies S1 to S14

Other Supplementary Material for this manuscript includes the following:

Tables S1 to S5
Movies S1 to S14

Supplementary Figure Legends

Fig. S1. Responses of the reporter lines to trans-zeatin (tZ) and 1-naphthaleneacetic acid (NAA) treatment.

(A to C) tdTomato signals in the roots of *TCSv2:tdTomato* transgenic rice plants treated with 10 µM *trans*-zeatin (tZ) for 6 or 24 h or mock-treated with water (A). Quantification of tdTomato signals in the root tip (B) and epidermis (C). Enlarged images of root cap cells (indicated by solid squares in (A)) and the outer region above the root cap (indicated by dotted squares in (A)) are shown in D and E, respectively. **(F to J)** Venus signals in the roots of *DR5rev:NLS-3xVenus* transgenic rice lines treated with 10 µM 1-naphthaleneacetic acid (NAA) for 6 or 24 h. Quantification of Venus signals in the root tip (I) and epidermis (J). The fluorescent signal intensity of each sample was normalized to fluorescence in the endodermis. Enlarged images of columella cells (indicated by solid squares in F) and the outer region above the root cap (indicated by dotted squares in F) are shown in I and J, respectively. Values are means ± SD (n = 6 for B and C, n = 4 for G and H); the P-values in B, C, G, and H were determined by a Student's t-test. Scale bars, 100 µm

Fig. S2. Imaging of the SAMs of DII-Venus transgenic rice lines at the vegetative phase.

Venus signals (A to C) and cross sections from 3D reconstructions of the SAMs from *DII-Venus* transgenic rice lines (D to F) at Stage I (A, D), Stage II (B, E), and Stage III (C, F). SAM, shoot apical meristem; P1, youngest leaf primordium (plastochron 1); P2, second youngest leaf primordium (plastochron 2). Scale bars, 25 µm.

Fig. S3. Imaging of the reproductive IMs of DII-Venus transgenic rice lines at the reproductive phase.

Venus signals (A to C) in longitudinal sections and cross sections of IMs from *DII-Venus* transgenic rice lines (D to F) at Stage R2 (A, D), Stage R3 (B, E), and Stage R4 (C, F). IM, inflorescence meristem; Br1, first bract; Br2, second bract; PBM, primary branch meristem; SBM, secondary branch meristem. Asterisks indicate bract hairs. Scale bars, 25 μm.

Fig. S4. Quantification of TCSv2 signal intensity in the SAMs of Koshihikari and NIL-Gn1.

(A and B) tdTomato signals in SAMs from *TCSv2:tdTomato* transgenic rice lines in the Koshihikari (A) and NIL-*Gn1* (B) backgrounds. **(C)** Relative fluorescence intensity of tdTomato in signal regions of the SAMs in Koshihikari and NIL-*Gn1*. The mean tdTomato signal intensity in Koshihikari after background subtraction was set to 1. SAM, shoot apical meristem; P1, youngest leaf primordium (plastochron 1). The *P*-value in C was determined by a Student's *t*-test. Scale bars, 25 μm.

Fig. S5. *OsFTIP1*, *OsFTIP10*, and *GF14f* expression in the IM.

(A to C) Relative expression levels measured by RT-qPCR of *OsFTIP1* (A), *OsFTIP10* (B), and *GF14f* (C) in the IMs of Koshihikari and NIL-*Gn1*. Values are means ± SD (n = 3); *P*-values were determined by a Student's *t*-test.

Fig. S6. Gene Ontology enrichment analysis of DEGs between Koshihikari and NIL-Gn1.

(A and B) Gene Ontology (GO) enrichment analysis of DEGs during the early stages of inflorescence development in the biological process (A) and cellular component (B) categories.

(**C to E**) GO enrichment analysis of DEGs during later stages of inflorescence development in the biological process (C), molecular function (D), and cellular component (E) categories.

Fig. S7. Expression of cytokinin-related genes in the IM.

(A) Simplified cytokinin biosynthesis, transport, and signal transduction pathways in plants. (**B)** Heatmap representation of the expression levels of cytokinin-related genes in Koshihikari (Koshi) and NIL-*Gn1* during early and later stages of inflorescence development. The average Log_2-transformed normalized UMI values from three to six replicates are shown.

Fig. S8. Expression of auxin-related genes in the IM.

(A to C) Simplified auxin biosynthesis (A), transport (B), and signal transduction (C) pathways in plants. (**D)** Heatmap representation of the expression levels of auxin-related genes in Koshihikari (Koshi) and NIL-*Gn1* during early and later stages of inflorescence development. The average Log_2-transformed normalized UMI values from three to six replicates are shown.

Fig. S9. Expression of cell cycle–related genes in the IM.

Heatmap representation of the expression levels of cell cycle–related genes in Koshihikari (Koshi) and NIL-*Gn1* in early and later stages of inflorescence development. The average Log_2-transformed normalized UMI values from three to six replicates are shown.

Fig. S10. Expression of genes regulating meristem function in the IM.

Heatmap representation of the expression levels of genes regulating meristem function in Koshihikari (Koshi) and NIL-*Gn1* in early and later stages of inflorescence development. The average Log_2-transformed normalized UMI values from three to six replicates are shown.

Fig. S11. Identification of genes regulated by the vegetative reproductive transition, NIL-*Gn1*, and inflorescence development.

(A) Venn diagram showing the extent of overlap between DEGs from the vegetative apex vs. reproductive apex comparison and DEGs from the Koshihikari SAM vs. NIL-*Gn1* SAM comparison. (**B)** Venn diagram showing the extent of overlap between genes identified in the overlap in **(A)** and genes that regulate inflorescence development (shown in Fig. S13). **(C)** Normalized unique molecular identifier (UMI) counts of *TAW1* in Koshihikari (Koshi) and NIL-*Gn1* at the later stage of IM development. (**D)** Relative expression levels of *TAW1* in Nipponbare (Nip) and *rolCpro:Hd3a-GFP*, as determined by RT-qPCR.

Fig. S12. Expression of genes regulating inflorescence development in the IM.

Heatmap representation of the expression levels of genes regulating inflorescence development in Koshihikari (Koshi) and NIL-*Gn1* during early and later stages of inflorescence development. The average Log_2-transformed normalized UMI values from three to six replicates are shown. *OsFTL1* and *TAW1* are indicated by blue filled circles.

Fig. S13. Expression of *Hd3a* in Nipponbare and *rolCpro:Hd3a-GFP*.

Relative expression levels of *TAW1* in Nipponbare (Nip) and *rolCpro:Hd3a-GFP*, as determined by RT-qPCR. Values represent means ± SD (n = 10 per genotype). Adjusted *P*-values were calculated using Student's *t*-test with Benjamini–Hochberg correction for false discovery rate.

Fig. S14. Genome editing of *OsFTL1*.

Diagram of the *OsFTL1* locus, nucleotide sequences corresponding to the sgRNA region and protospacer adjacent motif (PAM) for genome editing by CRISPR/Cas9, and deduced amino

acid sequences of OsFTL1 in wild type and the *Osftl1* mutants. Black boxes indicate exons; horizontal lines indicate untranslated regions and introns. Numbers indicate nucleotide positions from the first nucleotide (A) of the *OsFTL1* translation initiation codon. Insertions of T or A are shown in red, a deletion is indicated by a dashed line, and amino acids distinct from the wild-type protein due to the frameshift insertions are shown in red.

Fig. S15. Expression of *OsFTL1* and phenotypes of the *Osftl1* mutant under long-day conditions.

(A to F) Grain number (A), number of primary branches (B), number of spikelets and secondary branches per primary branch (C), number of spikelets per secondary branch (D), and days to heading (E) of Nipponbare and *Osftl1-insT*. Values are means ± SD (n = 5 for A, n = 16 for Koshihikari, n = 15 for *Osftl1-insT* in B-E); P-values were determined by a Student's t-test.

Fig. S16. Phenotypes of *OsFTL1pro:OsFTL1-Clover* plants in the *Osftl1* mutant background.

(A to E) Grain number (A), number of primary branches (B), number of spikelets and secondary branches per primary branch (C), number of spikelets per secondary branch (D), and days to heading (E) in Nipponbare, *Osftl1-insT*, and *Osftl1-insT OsFTL1pro:OsFTL1-Clover*. Values are means ± SD (n = 16 for Nip, n = 20 for *Osftl1-insT*, n = 12 for *Osftl1-insT OsFTL1pro:OsFTL1-Clover*); different lowercase letters indicate significant differences (Tukey's test, $\alpha = 0.05$).

Fig. S17. ***OsFTL1*** **promoter activity in the IM.**

(A to D) Distribution of Clover fluorescence in the IMs of *OsFTL1pro:NLS-3xVenus* transgenic rice lines (same image as Fig. 7C–F; shown to facilitate comparison). (**E to H)** Cross section from the 3D reconstruction of (A–D). Images at Stage R2 (A, E) and Stage R3 (B to D, F to H) are shown. IM, inflorescence meristem; Br1, first bract; Br2, second bract; PBM, primary branch meristem; IPB, incipient bract. Arrowheads in A–D indicate the positions of the cross sections in E to H. Scale bars, 25 μm.

Fig. S18. GO enrichment analysis of DEGs of Nipponbare and ***Osftl1-insT*** **at Stages R3 and R4.**

(**A)** Venn diagram showing the extent of overlap between upregulated and downregulated DEGs in Nipponbare and *Osftl1-insT* at Stages R3 and R4. (B to E) GO enrichment analysis of upregulated or downregulated DEGs for biological processes at Stage R3 (B and C) and Stage R4 (D and E).

Fig. S19. Expression of cytokinin-related genes in the IM.

Heatmap representation of the expression levels of cytokinin-related genes in Nipponbare (Nip) and *Osftl1-insT* at Stages R3 and R4 of inflorescence development. The average Log_2-transformed normalized UMI values from 14–24 replicates are shown. DEGs at Stage R3 are indicated by blue filled circles.

Fig. S20. Expression of auxin-related genes in the IM.

Heatmap representation of the expression levels of auxin-related genes in Nipponbare (Nip) and *Osftl1-insT* at Stages R3 and R4 of inflorescence development. The average Log_2-

transformed normalized UMI values from 14–24 replicates are shown. DEGs at Stages R3 and R4 are marked with blue and magenta filled circles, respectively.

Fig. S21. Expression of cell cycle–related genes in the IM.

Heatmap representation of the expression levels of cell cycle-related genes in Nipponbare (Nip) and *Osftl1-insT* at Stages R3 and R4 of inflorescence development. The average Log_2-transformed normalized UMI values from 14–24 replicates are shown. DEGs at Stage R3 are marked with blue filled circles.

Fig. S22. Expression of genes regulating inflorescence development in the IM.

Heatmap representation of the expression levels of inflorescence development-related genes in Nipponbare (Nip) and *Osftl1-insT* at Stages R3 and R4 of inflorescence development. The average Log_2-transformed normalized UMI values from 14–24 replicates are shown. DEGs at Stage R3 and R4 are marked with blue and magenta filled circles, respectively.

Fig. S23. Expression of genes regulating meristem function in the IM.

Heatmap representation of the expression levels of meristem function-related genes in Nipponbare (Nip) and *Osftl1-insT* at Stages R3 and R4 of inflorescence development. The average Log_2-transformed normalized UMI values from 14–24 replicates are shown. DEGs at Stage R3 are marked with blue filled circles.

Fig. S24. Normalized unique molecular identifier counts of DEGs in Nipponbare and *Osftl1-insT* at Stages R3 and R4.

(**A to H)** Normalized unique molecular identifier (UMI**)** counts of *OsMADS14* (A), *OsMADS15* (B), *OsMADS34* (C), *OSH1* (D), *FON1* (E), *FOL2* (F), *OsSPL14* (G), and *TAW1*

(H) in Nipponbare (Nip) and *Osftl1-insT* at Stages R3 and R4. Values are means ± SD (n = 20 for Nip and n = 24 for *Osftl1-insT* for R3 and n = 22 for Nip and n = 14 for *Osftl1-insT* for R4).

Fig. S25. Identification of genes regulated by the vegetative-to-reproductive transition and in *Osftl1-insT*.

(A to D) Venn diagrams showing the extent of overlap between DEGs from the vegetative apex vs. the reproductive apex and DEGs from the Nipponbare IM vs. *Osftl1-insT* IM. The panels depict genes upregulated in both the reproductive apex and Osftl1-insT (A), genes upregulated in the reproductive apex and in Nipponbare (B), genes downregulated in the reproductive apex but upregulated in Osftl1-insT (C), and genes downregulated in both the reproductive apex and Osftl1-insT (D). GO enrichment analysis of biological processes is shown for each category.

Fig. S26. Distribution of GFP and mOrange fluorescence in the IM of the *Hd3apro:Hd3a-GFP OsMADS15-mOrange gene targeting* double transgenic line.

(A to D) Distribution of GFP (A, C) and mOrange (B, D) fluorescence signals in the IM of the *Hd3apro:Hd3a-GFP OsMADS15-mOrange gene targeting* double transgenic line at Stage R2.

Fig. S27. Diagram illustrating the proposed spatial distribution of the fluorescent signals from the indicated reporters in the meristem at Stages V, R2, and R3.

Schematic representations summarize the observed fluorescence patterns in the SAM and IM at the vegetative stage (Stage V), early reproductive stage (Stage R2), and late reproductive stage (Stage R3). Each row corresponds to a different reporter line.

Supplementary tables

Table S1

DEGs between Koshihikari and NIL-*Gn1* during the early stages of inflorescence development.

Table S2

DEGs between Koshihikari and NIL-*Gn1* during the later stages of inflorescence development.

Table S3

DEGs between Nipponbare and *Osftl1* at stage R3 of inflorescence development.

Table S4

DEGs between Nipponbare and *Osftl1* at stage R4 of inflorescence development.

Table S5

Primers used in this study.

Supplementary movies

Movie S1

SAM at Stage I for the *TCSv2:tdTomato DR5rev:NLS-3xVenus* double transgenic rice.

Movie S2

SAM at Stage II for the *TCSv2:tdTomato DR5rev:NLS-3xVenus* double transgenic rice line.

Movie S3

SAM at Stage III for the *TCSv2:tdTomato DR5rev:NLS-3xVenus* double transgenic rice line.

Movie S4

SAM at Stage IV for the *TCSv2:tdTomato DR5rev:NLS-3xVenus* double transgenic rice line.

Movie S5

SAM at Stages I–III for the *DII-Venus* transgenic rice line.

Movie S6

IM at Stage R1 for the *TCSv2:tdTomato* transgenic rice line.

Movie S7

IM at Stage R2 for the *TCSv2:tdTomato* transgenic rice line.

Movie S8

IM at Stages R2 and R3 for the *TCSv2:tdTomato DR5rev:NLS-3xVenus* double transgenic rice line.

Movie S9

IM at Stage R2 for the *DII-Venus* transgenic rice line.

Movie S10

IM at Stage R3 for the *DII-Venus* transgenic rice line.

Movie S11

IM at Stage R4 for the *DII-Venus* transgenic rice line.

Movie S12

IM at Stage R2 for the *Hd3apro:Hd3a-GFP* transgenic rice line, imaged by two-photon excitation microscopy.

Movie S13

IM at Stage R3 for the *OsFTL1pro:NLS-3xVenus* transgenic rice line.

Movie S14

IM and PBM at Stage R4 for the *OsFTL1pro:NLS-3xVenus* transgenic rice line.

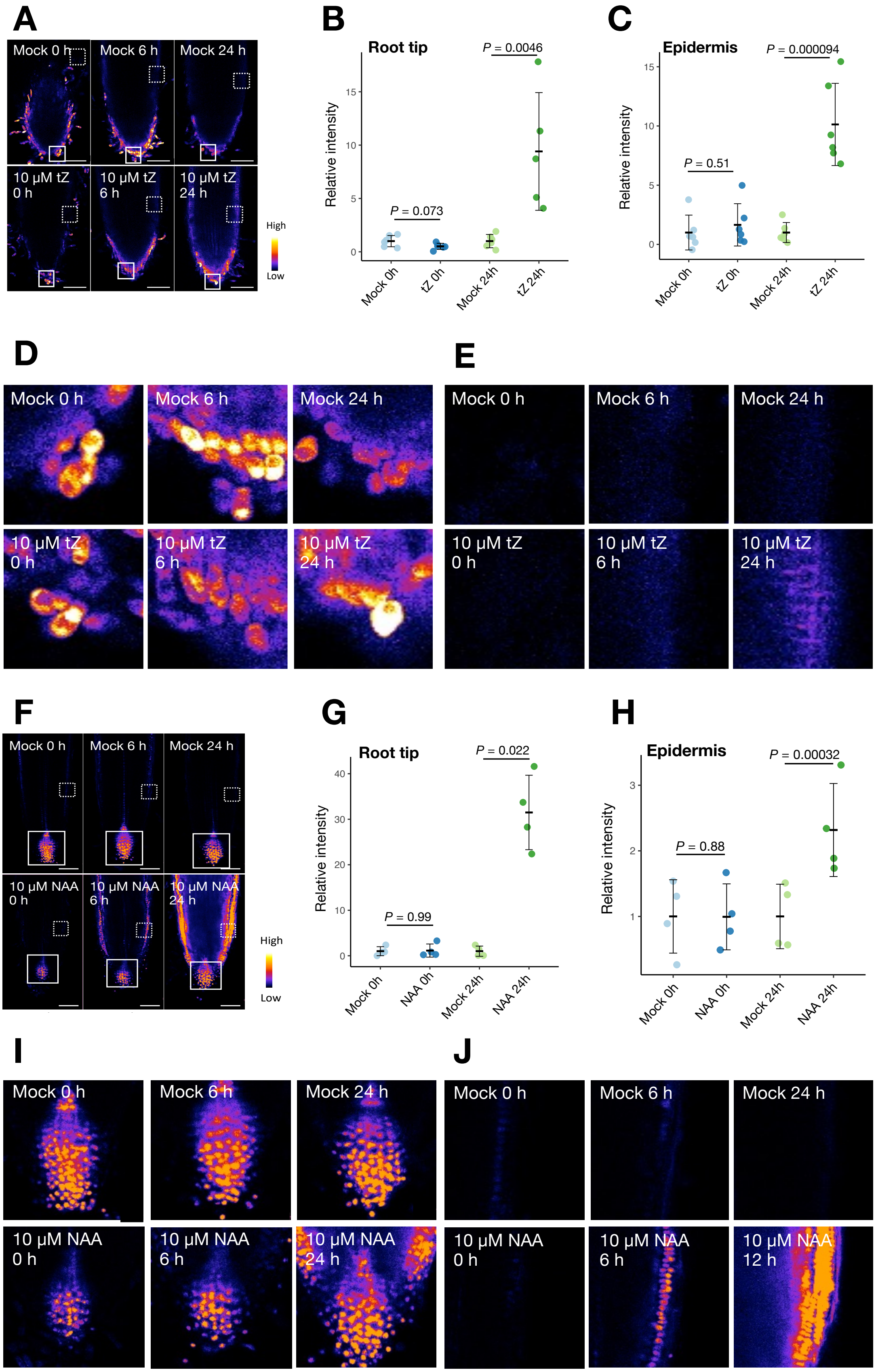
A
Mock 0 h
Mock 6 h
Mock 24 h
10 μM tZ 0 h
10 μM tZ 6 h
10 μM tZ 24 h
High
Low
B
Root tip
P = 0.0046
P = 0.073
Relative intensity
Mock 0h
tZ 0h
Mock 24h
tZ 24h
C
Epidermis
P = 0.000094
P = 0.51
Relative intensity
Mock 0h
tZ 0h
Mock 24h
tZ 24h
D
Mock 0 h
Mock 6 h
Mock 24 h
10 μM tZ 0 h
10 μM tZ 6 h
10 μM tZ 24 h
E
Mock 0 h
Mock 6 h
Mock 24 h
10 μM tZ 0 h
10 μM tZ 6 h
10 μM tZ 24 h
F
Mock 0 h
Mock 6 h
Mock 24 h
10 μM NAA 0 h
10 μM NAA 6 h
10 μM NAA 24 h
High
Low
G
Root tip
P = 0.022
P = 0.99
Relative intensity
Mock 0h
NAA 0h
Mock 24h
NAA 24h
H
Epidermis
P = 0.00032
P = 0.88
Relative intensity
Mock 0h
NAA 0h
Mock 24h
NAA 24h
I
Mock 0 h
Mock 6 h
Mock 24 h
10 μM NAA 0 h
10 μM NAA 6 h
10 μM NAA 24 h
J
Mock 0 h
Mock 6 h
Mock 24 h
10 μM NAA 0 h
10 μM NAA 6 h
10 μM NAA 12 h

Fig. S1

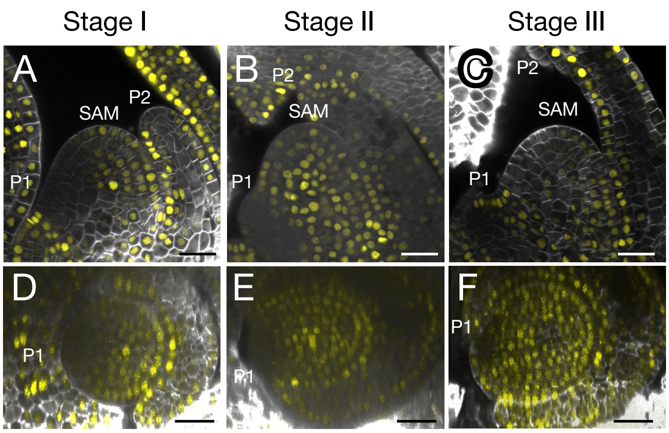
Stage I
Stage II
Stage III
A
SAM
P2
P1
B
P2
SAM
P1
C
P2
SAM
P1
D
P1
E
P1
F
P1

Fig. S2

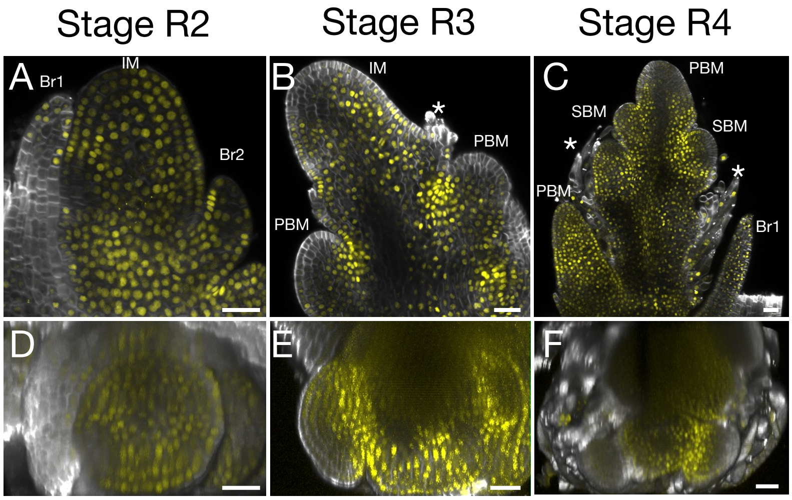
Stage R2
Stage R3
Stage R4
A
Br1
IM
Br2
B
IM
*
PBM
PBM
C
PBM
SBM
SBM
*
*
PBM
Br1
D
E
F

Fig. S3

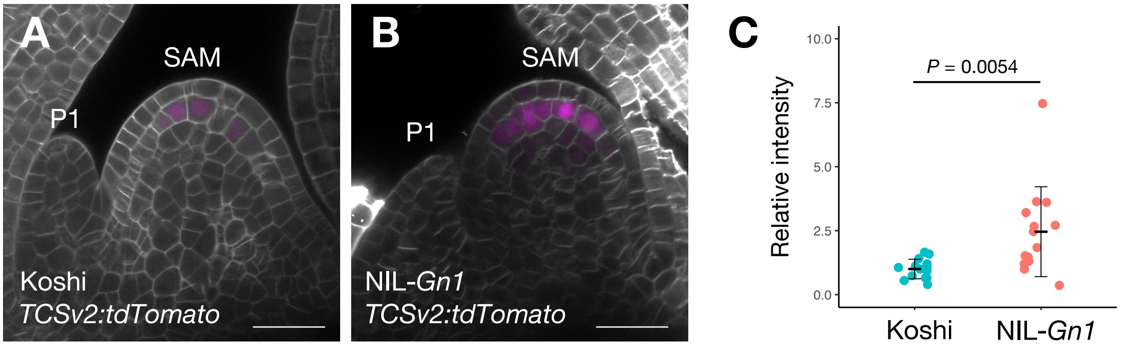
A
SAM
P1
Koshi
TCSv2:tdTomato
B
SAM
P1
NIL-Gn1
TCSv2:tdTomato
C
P = 0.0054
Relative intensity
10.0
7.5
5.0
2.5
0.0
Koshi
NIL-Gn1

Fig. S4

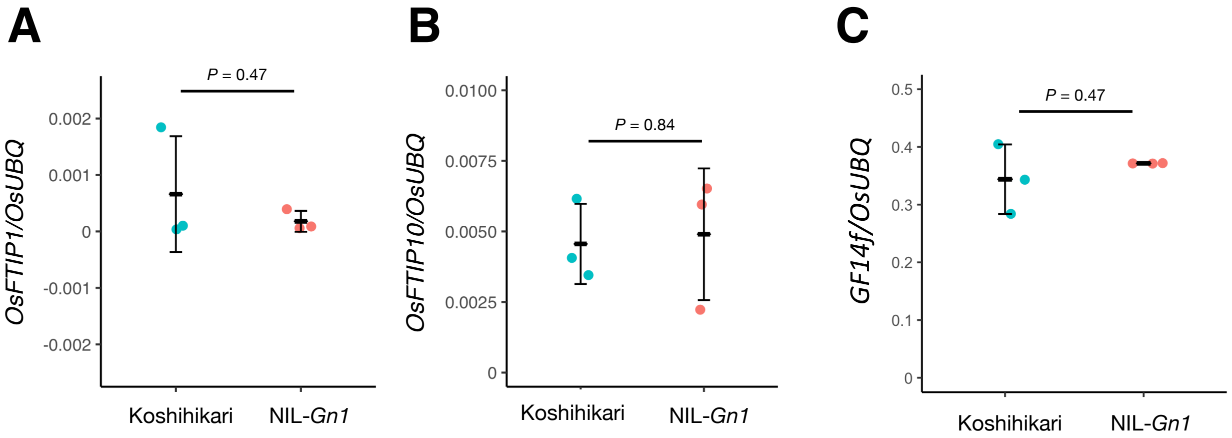

A
P = 0.47
0.002
0.001
0
-0.001
-0.002
OsFTIP1/OsUBQ
Koshihikari
NIL-Gn1
B
0.0100
0.0075
0.0050
0.0025
0
P = 0.84
OsFTIP10/OsUBQ
Koshihikari
NIL-Gn1
C
0.5
0.4
0.3
0.2
0.1
0
P = 0.47
GF14f/OsUBQ
Koshihikari
NIL-Gn1

Fig. S5

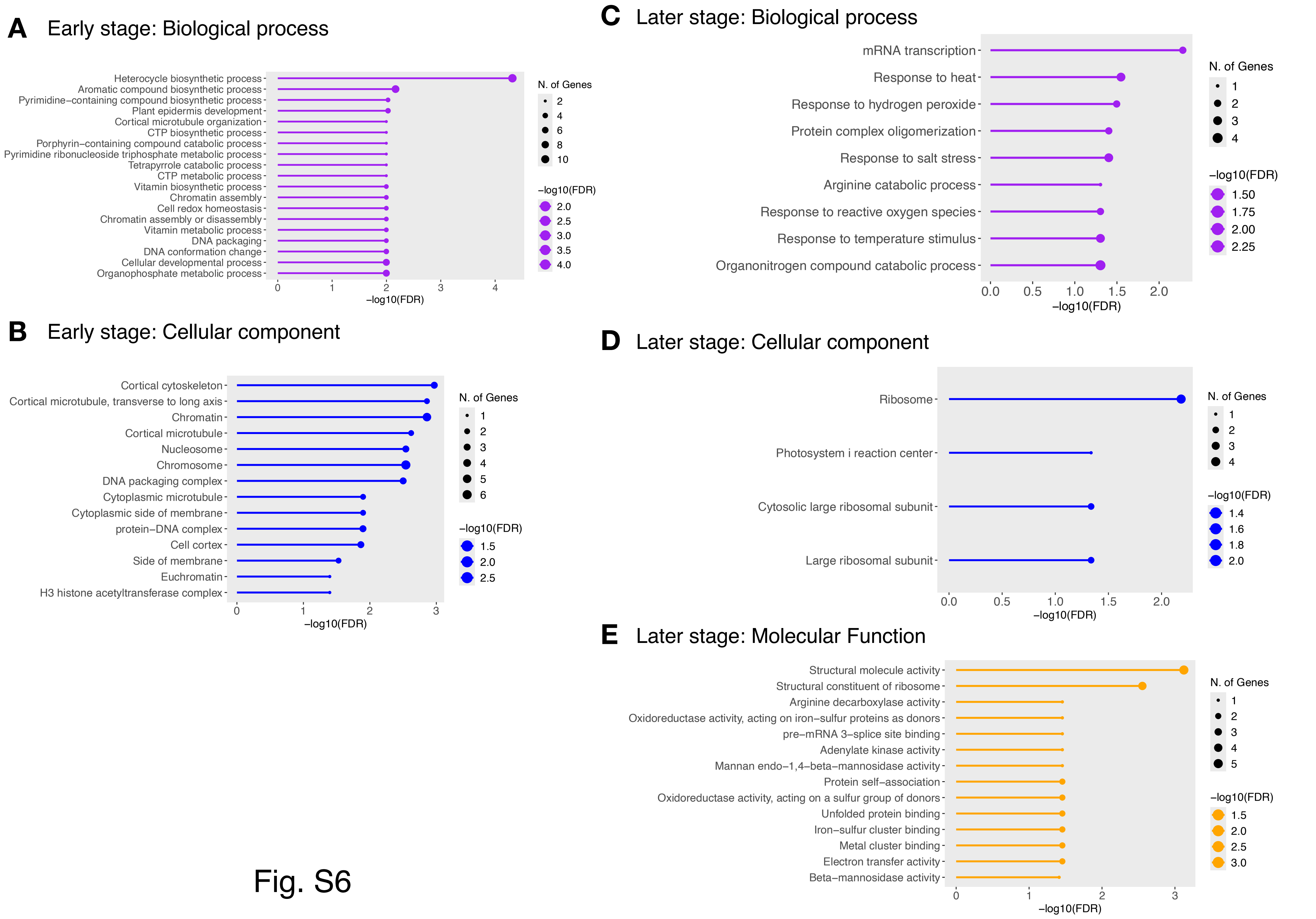
A
Early stage: Biological process
Heterocycle biosynthetic process
Aromatic compound biosynthetic process
Pyrimidine–containing compound biosynthetic process
Plant epidermis development
Cortical microtubule organization
CTP biosynthetic process
Porphyrin–containing compound catabolic process
Pyrimidine ribonucleoside triphosphate metabolic process
Tetrapyrrole catabolic process
CTP metabolic process
Vitamin biosynthetic process
Chromatin assembly
Cell redox homeostasis
Chromatin assembly or disassembly
Vitamin metabolic process
DNA packaging
DNA conformation change
Cellular developmental process
Organophosphate metabolic process
–log10(FDR)
N. of Genes
2
4
6
8
10
–log10(FDR)
2.0
2.5
3.0
3.5
4.0
B
Early stage: Cellular component
Cortical cytoskeleton
Cortical microtubule, transverse to long axis
Chromatin
Cortical microtubule
Nucleosome
Chromosome
DNA packaging complex
Cytoplasmic microtubule
Cytoplasmic side of membrane
protein–DNA complex
Cell cortex
Side of membrane
Euchromatin
H3 histone acetyltransferase complex
–log10(FDR)
N. of Genes
1
2
3
4
5
6
–log10(FDR)
1.5
2.0
2.5
C
Later stage: Biological process
mRNA transcription
Response to heat
Response to hydrogen peroxide
Protein complex oligomerization
Response to salt stress
Arginine catabolic process
Response to reactive oxygen species
Response to temperature stimulus
Organonitrogen compound catabolic process
–log10(FDR)
N. of Genes
1
2
3
4
–log10(FDR)
1.50
1.75
2.00
2.25
D
Later stage: Cellular component
Ribosome
Photosystem i reaction center
Cytosolic large ribosomal subunit
Large ribosomal subunit
–log10(FDR)
N. of Genes
1
2
3
4
–log10(FDR)
1.4
1.6
1.8
2.0
E
Later stage: Molecular Function
Structural molecule activity
Structural constituent of ribosome
Arginine decarboxylase activity
Oxidoreductase activity, acting on iron–sulfur proteins as donors
pre–mRNA 3–splice site binding
Adenylate kinase activity
Mannan endo–1,4–beta–mannosidase activity
Protein self–association
Oxidoreductase activity, acting on a sulfur group of donors
Unfolded protein binding
Iron–sulfur cluster binding
Metal cluster binding
Electron transfer activity
Beta–mannosidase activity
–log10(FDR)
N. of Genes
1
2
3
4
5
–log10(FDR)
1.5
2.0
2.5
3.0

Fig. S6

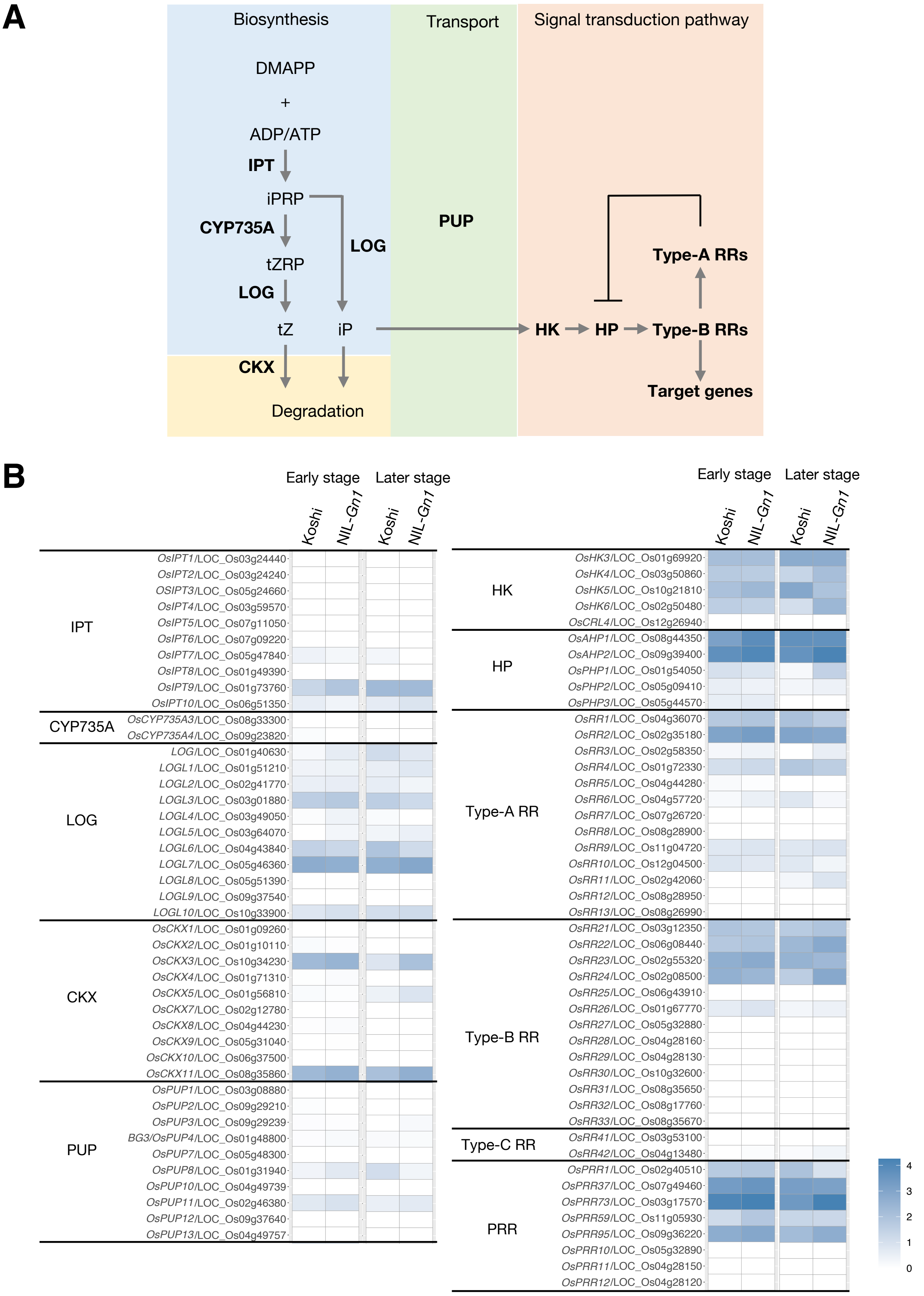
A
Biosynthesis
Transport
Signal transduction pathway
DMAPP
+
ADP/ATP
IPT
iPRP
CYP735A
LOG
tZRP
LOG
tZ
iP
CKX
Degradation
PUP
HK
HP
Type-A RRs
Type-B RRs
Target genes
B
Early stage
Later stage
Koshi
NIL-Gn1
IPT
OsIPT1/LOC_Os03g24440
OsIPT2/LOC_Os03g24240
OSIPT3/LOC_Os05g24660
OsIPT4/LOC_Os03g59570
OsIPT5/LOC_Os07g11050
OsIPT6/LOC_Os07g09220
OsIPT7/LOC_Os05g47840
OsIPT8/LOC_Os01g49390
OsIPT9/LOC_Os01g73760
OsIPT10/LOC_Os06g51350
CYP735A
OsCYP735A3/LOC_Os08g33300
OsCYP735A4/LOC_Os09g23820
LOG
LOG/LOC_Os01g40630
LOGL1/LOC_Os01g51210
LOGL2/LOC_Os02g41770
LOGL3/LOC_Os03g01880
LOGL4/LOC_Os03g49050
LOGL5/LOC_Os03g64070
LOGL6/LOC_Os04g43840
LOGL7/LOC_Os05g46360
LOGL8/LOC_Os05g51390
LOGL9/LOC_Os09g37540
LOGL10/LOC_Os10g33900
CKX
OsCKX1/LOC_Os01g09260
OsCKX2/LOC_Os01g10110
OsCKX3/LOC_Os10g34230
OsCKX4/LOC_Os01g71310
OsCKX5/LOC_Os01g56810
OsCKX7/LOC_Os02g12780
OsCKX8/LOC_Os04g44230
OsCKX9/LOC_Os05g31040
OsCKX10/LOC_Os06g37500
OsCKX11/LOC_Os08g35860
PUP
OsPUP1/LOC_Os03g08880
OsPUP2/LOC_Os09g29210
OsPUP3/LOC_Os09g29239
BG3/OsPUP4/LOC_Os01g48800
OsPUP7/LOC_Os05g48300
OsPUP8/LOC_Os01g31940
OsPUP10/LOC_Os04g49739
OsPUP11/LOC_Os02g46380
OsPUP12/LOC_Os09g37640
OsPUP13/LOC_Os04g49757
HK
OsHK3/LOC_Os01g69920
OsHK4/LOC_Os03g50860
OsHK5/LOC_Os10g21810
OsHK6/LOC_Os02g50480
OsCRL4/LOC_Os12g26940
HP
OsAHP1/LOC_Os08g44350
OsAHP2/LOC_Os09g39400
OsPHP1/LOC_Os01g54050
OsPHP2/LOC_Os05g09410
OsPHP3/LOC_Os05g44570
Type-A RR
OsRR1/LOC_Os04g36070
OsRR2/LOC_Os02g35180
OsRR3/LOC_Os02g58350
OsRR4/LOC_Os01g72330
OsRR5/LOC_Os04g44280
OsRR6/LOC_Os04g57720
OsRR7/LOC_Os07g26720
OsRR8/LOC_Os08g28900
OsRR9/LOC_Os11g04720
OsRR10/LOC_Os12g04500
OsRR11/LOC_Os02g42060
OsRR12/LOC_Os08g28950
OsRR13/LOC_Os08g26990
Type-B RR
OsRR21/LOC_Os03g12350
OsRR22/LOC_Os06g08440
OsRR23/LOC_Os02g55320
OsRR24/LOC_Os02g08500
OsRR25/LOC_Os06g43910
OsRR26/LOC_Os01g67770
OsRR27/LOC_Os05g32880
OsRR28/LOC_Os04g28160
OsRR29/LOC_Os04g28130
OsRR30/LOC_Os10g32600
OsRR31/LOC_Os08g35650
OsRR32/LOC_Os08g17760
OsRR33/LOC_Os08g35670
Type-C RR
OsRR41/LOC_Os03g53100
OsRR42/LOC_Os04g13480
PRR
OsPRR1/LOC_Os02g40510
OsPRR37/LOC_Os07g49460
OsPRR73/LOC_Os03g17570
OsPRR59/LOC_Os11g05930
OsPRR95/LOC_Os09g36220
OsPRR10/LOC_Os05g32890
OsPRR11/LOC_Os04g28150
OsPRR12/LOC_Os04g28120
4
3
2
1
0

Fig. S7

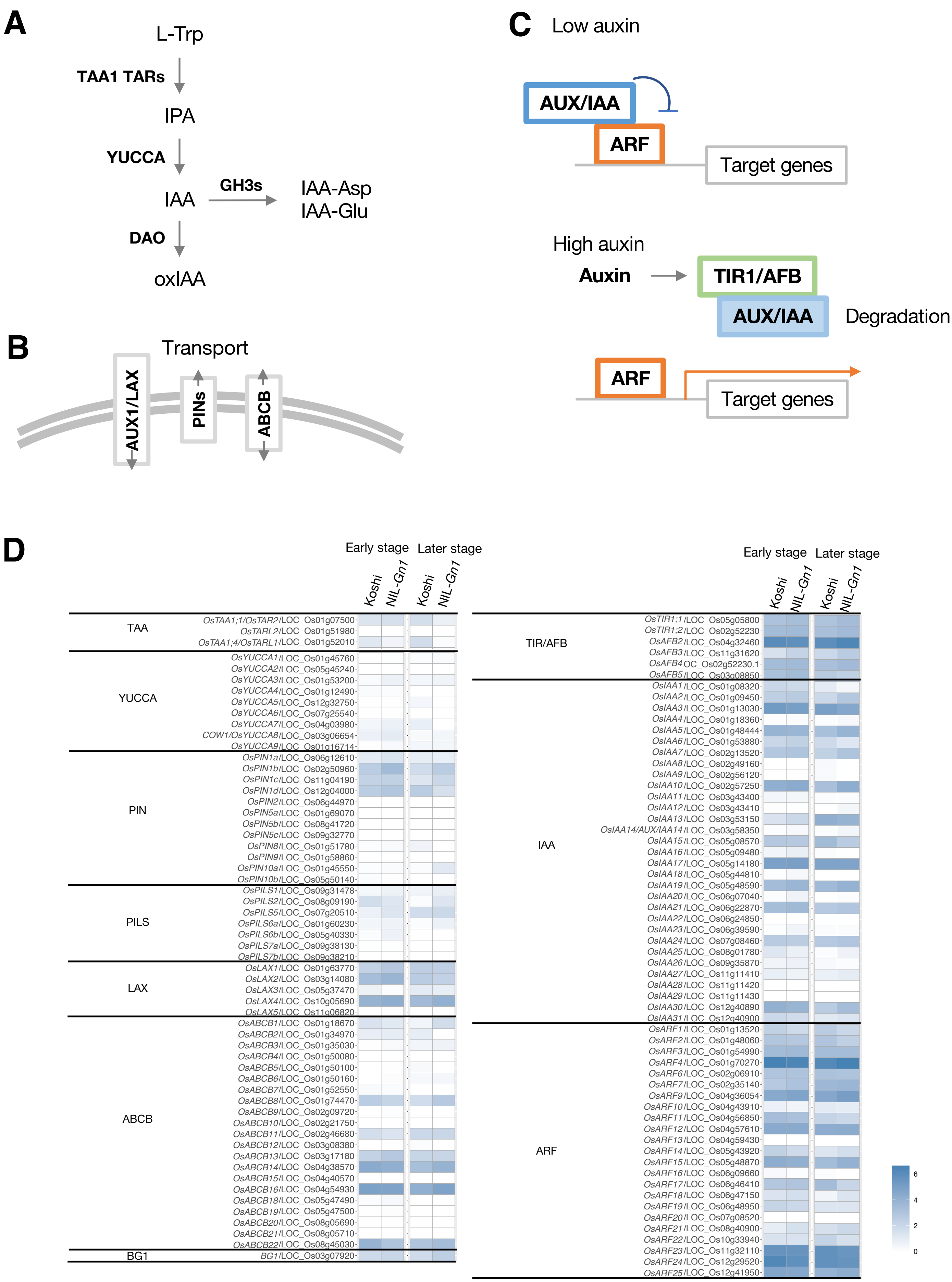
A
L-Trp
TAA1 TARs
IPA
YUCCA
IAA
GH3s
IAA-Asp
IAA-Glu
DAO
oxIAA
B
Transport
AUX1/LAX
PINs
ABCB
C
Low auxin
AUX/IAA
ARF
Target genes
High auxin
Auxin
TIR1/AFB
AUX/IAA
Degradation
ARF
Target genes
D
Early stage
Later stage
Koshi
NIL-Gn1
Koshi
NIL-Gn1
TAA
OsTAA1;1/OsTAR2/LOC_Os01g07500
OsTARL2/LOC_Os01g51980
OsTAA1;4/OsTARL1/LOC_Os01g52010
YUCCA
OsYUCCA1/LOC_Os01g45760
OsYUCCA2/LOC_Os05g45240
OsYUCCA3/LOC_Os01g53200
OsYUCCA4/LOC_Os01g12490
OsYUCCA5/LOC_Os12g32750
OsYUCCA6/LOC_Os07g25540
OsYUCCA7/LOC_Os04g03980
COW1/OsYUCCA8/LOC_Os03g06654
OsYUCCA9/LOC_Os01g16714
PIN
OsPIN1a/LOC_Os06g12610
OsPIN1b/LOC_Os02g50960
OsPIN1c/LOC_Os11g04190
OsPIN1d/LOC_Os12g04000
OsPIN2/LOC_Os06g44970
OsPIN5a/LOC_Os01g69070
OsPIN5b/LOC_Os08g41720
OsPIN5c/LOC_Os09g32770
OsPIN8/LOC_Os01g51780
OsPIN9/LOC_Os01g58860
OsPIN10a/LOC_Os01g45550
OsPIN10b/LOC_Os05g50140
PILS
OsPILS1/LOC_Os09g31478
OsPILS2/LOC_Os08g09190
OsPILS5/LOC_Os07g20510
OsPILS6a/LOC_Os01g60230
OsPILS6b/LOC_Os05g40330
OsPILS7a/LOC_Os09g38130
OsPILS7b/LOC_Os09g38210
LAX
OsLAX1/LOC_Os01g63770
OsLAX2/LOC_Os03g14080
OsLAX3/LOC_Os05g37470
OsLAX4/LOC_Os10g05690
OsLAX5/LOC_Os11g06820
ABCB
OsABCB1/LOC_Os01g18670
OsABCB2/LOC_Os01g34970
OsABCB3/LOC_Os01g35030
OsABCB4/LOC_Os01g50080
OsABCB5/LOC_Os01g50100
OsABCB6/LOC_Os01g50160
OsABCB7/LOC_Os01g52550
OsABCB8/LOC_Os01g74470
OsABCB9/LOC_Os02g09720
OsABCB10/LOC_Os02g21750
OsABCB11/LOC_Os02g46680
OsABCB12/LOC_Os03g08380
OsABCB13/LOC_Os03g17180
OsABCB14/LOC_Os04g38570
OsABCB15/LOC_Os04g40570
OsABCB16/LOC_Os04g54930
OsABCB18/LOC_Os05g47490
OsABCB19/LOC_Os05g47500
OsABCB20/LOC_Os08g05690
OsABCB21/LOC_Os08g05710
OsABCB22/LOC_Os08g45030
BG1
BG1/LOC_Os03g07920
Early stage
Later stage
Koshi
NIL-Gn1
Koshi
NIL-Gn1
TIR/AFB
OsTIR1;1/LOC_Os05g05800
OsTIR1;2/LOC_Os02g52230
OsAFB2/LOC_Os04g32460
OsAFB3/LOC_Os11g31620
OsAFB4 OC_Os02g52230.1
OsAFB5/LOC_Os03g08850
IAA
OsIAA1/LOC_Os01g08320
OsIAA2/LOC_Os01g09450
OsIAA3/LOC_Os01g13030
OsIAA4/LOC_Os01g18360
OsIAA5/LOC_Os01g48444
OsIAA6/LOC_Os01g53880
OsIAA7/LOC_Os02g13520
OsIAA8/LOC_Os02g49160
OsIAA9/LOC_Os02g56120
OsIAA10/LOC_Os02g57250
OsIAA11/LOC_Os03g43400
OsIAA12/LOC_Os03g43410
OsIAA13/LOC_Os03g53150
OsIAA14/AUX/IAA14/LOC_Os03g58350
OsIAA15/LOC_Os05g08570
OsIAA16/LOC_Os05g09480
OsIAA17/LOC_Os05g14180
OsIAA18/LOC_Os05g44810
OsIAA19/LOC_Os05g48590
OsIAA20/LOC_Os06g07040
OsIAA21/LOC_Os06g22870
OsIAA22/LOC_Os06g24850
OsIAA23/LOC_Os06g39590
OsIAA24/LOC_Os07g08460
OsIAA25/LOC_Os08g01780
OsIAA26/LOC_Os09g35870
OsIAA27/LOC_Os11g11410
OsIAA28/LOC_Os11g11420
OsIAA29/LOC_Os11g11430
OsIAA30/LOC_Os12g40890
OsIAA31/LOC_Os12g40900
ARF
OsARF1/LOC_Os01g13520
OsARF2/LOC_Os01g48060
OsARF3/LOC_Os01g54990
OsARF4/LOC_Os01g70270
OsARF6/LOC_Os02g06910
OsARF7/LOC_Os02g35140
OsARF9/LOC_Os04g36054
OsARF10/LOC_Os04g43910
OsARF11/LOC_Os04g56850
OsARF12/LOC_Os04g57610
OsARF13/LOC_Os04g59430
OsARF14/LOC_Os05g43920
OsARF15/LOC_Os05g48870
OsARF16/LOC_Os06g09660
OsARF17/LOC_Os06g46410
OsARF18/LOC_Os06g47150
OsARF19/LOC_Os06g48950
OsARF20/LOC_Os07g08520
OsARF21/LOC_Os08g40900
OsARF22/LOC_Os10g33940
OsARF23/LOC_Os11g32110
OsARF24/LOC_Os12g29520
OsARF25/LOC_Os12g41950
6
4
2
0

Fig. S8

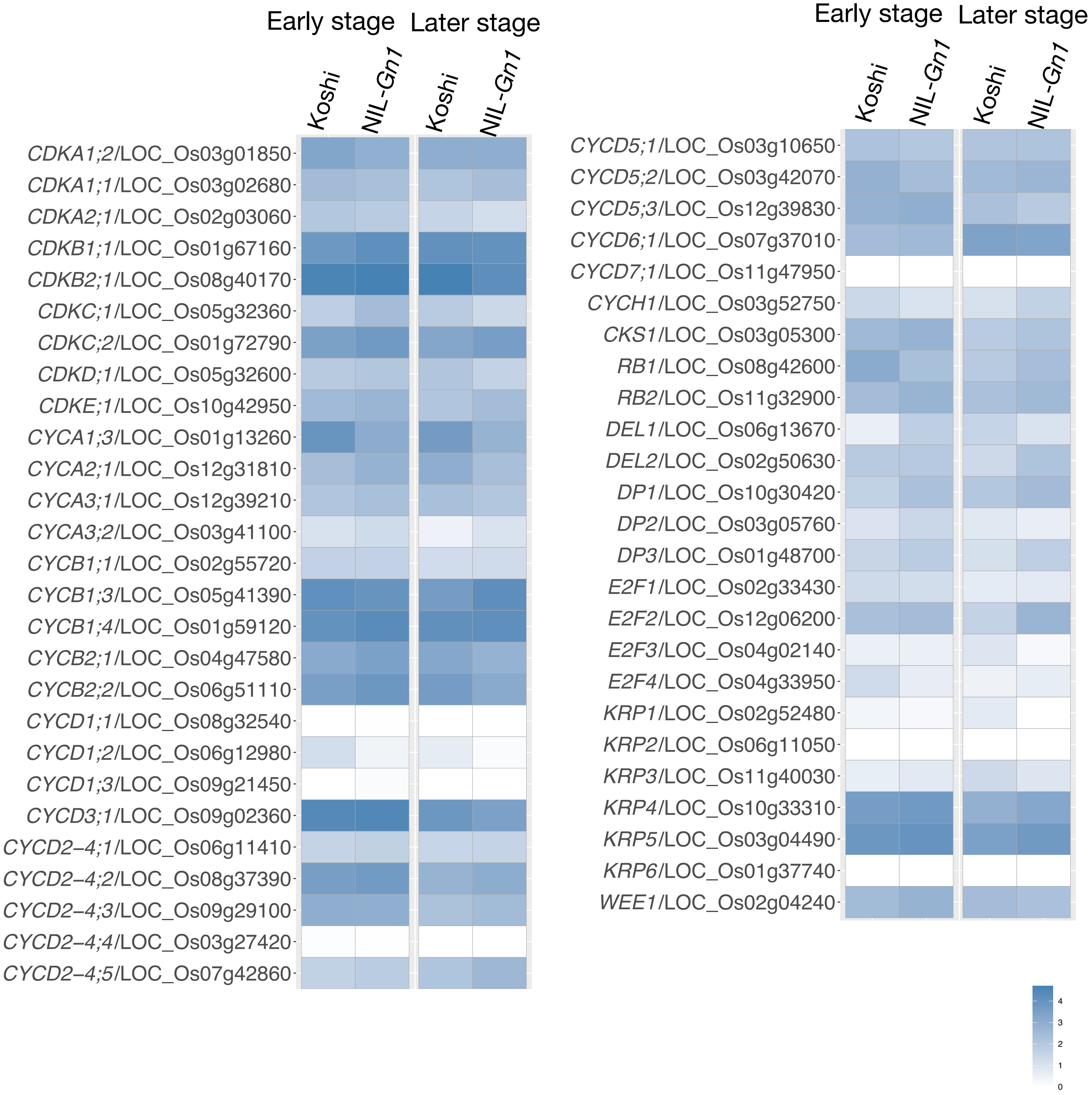
Early stage
Later stage
Koshi
NIL-Gn1
Koshi
NIL-Gn1
CDKA1;2/LOC_Os03g01850
CDKA1;1/LOC_Os03g02680
CDKA2;1/LOC_Os02g03060
CDKB1;1/LOC_Os01g67160
CDKB2;1/LOC_Os08g40170
CDKC;1/LOC_Os05g32360
CDKC;2/LOC_Os01g72790
CDKD;1/LOC_Os05g32600
CDKE;1/LOC_Os10g42950
CYCA1;3/LOC_Os01g13260
CYCA2;1/LOC_Os12g31810
CYCA3;1/LOC_Os12g39210
CYCA3;2/LOC_Os03g41100
CYCB1;1/LOC_Os02g55720
CYCB1;3/LOC_Os05g41390
CYCB1;4/LOC_Os01g59120
CYCB2;1/LOC_Os04g47580
CYCB2;2/LOC_Os06g51110
CYCD1;1/LOC_Os08g32540
CYCD1;2/LOC_Os06g12980
CYCD1;3/LOC_Os09g21450
CYCD3;1/LOC_Os09g02360
CYCD2–4;1/LOC_Os06g11410
CYCD2–4;2/LOC_Os08g37390
CYCD2–4;3/LOC_Os09g29100
CYCD2–4;4/LOC_Os03g27420
CYCD2–4;5/LOC_Os07g42860
Early stage
Later stage
Koshi
NIL-Gn1
Koshi
NIL-Gn1
CYCD5;1/LOC_Os03g10650
CYCD5;2/LOC_Os03g42070
CYCD5;3/LOC_Os12g39830
CYCD6;1/LOC_Os07g37010
CYCD7;1/LOC_Os11g47950
CYCH1/LOC_Os03g52750
CKS1/LOC_Os03g05300
RB1/LOC_Os08g42600
RB2/LOC_Os11g32900
DEL1/LOC_Os06g13670
DEL2/LOC_Os02g50630
DP1/LOC_Os10g30420
DP2/LOC_Os03g05760
DP3/LOC_Os01g48700
E2F1/LOC_Os02g33430
E2F2/LOC_Os12g06200
E2F3/LOC_Os04g02140
E2F4/LOC_Os04g33950
KRP1/LOC_Os02g52480
KRP2/LOC_Os06g11050
KRP3/LOC_Os11g40030
KRP4/LOC_Os10g33310
KRP5/LOC_Os03g04490
KRP6/LOC_Os01g37740
WEE1/LOC_Os02g04240
4
3
2
1
0

Fig. S9

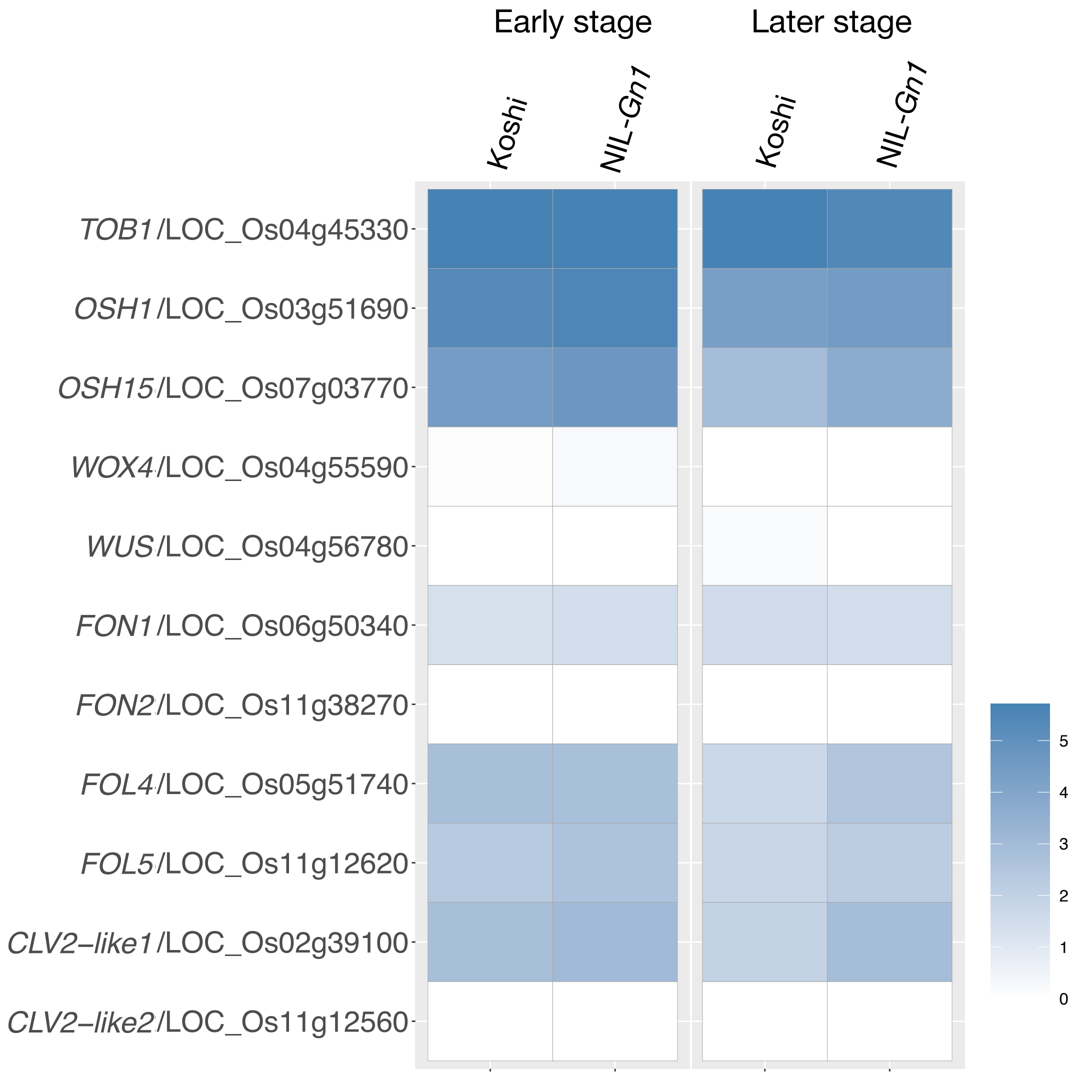

Early stage
Later stage
Koshi
NIL-Gn1
Koshi
NIL-Gn1
TOB1/LOC_Os04g45330
OSH1/LOC_Os03g51690
OSH15/LOC_Os07g03770
WOX4/LOC_Os04g55590
WUS/LOC_Os04g56780
FON1/LOC_Os06g50340
FON2/LOC_Os11g38270
FOL4/LOC_Os05g51740
FOL5/LOC_Os11g12620
CLV2–like1/LOC_Os02g39100
CLV2–like2/LOC_Os11g12560
5
4
3
2
1
0

Fig. S10

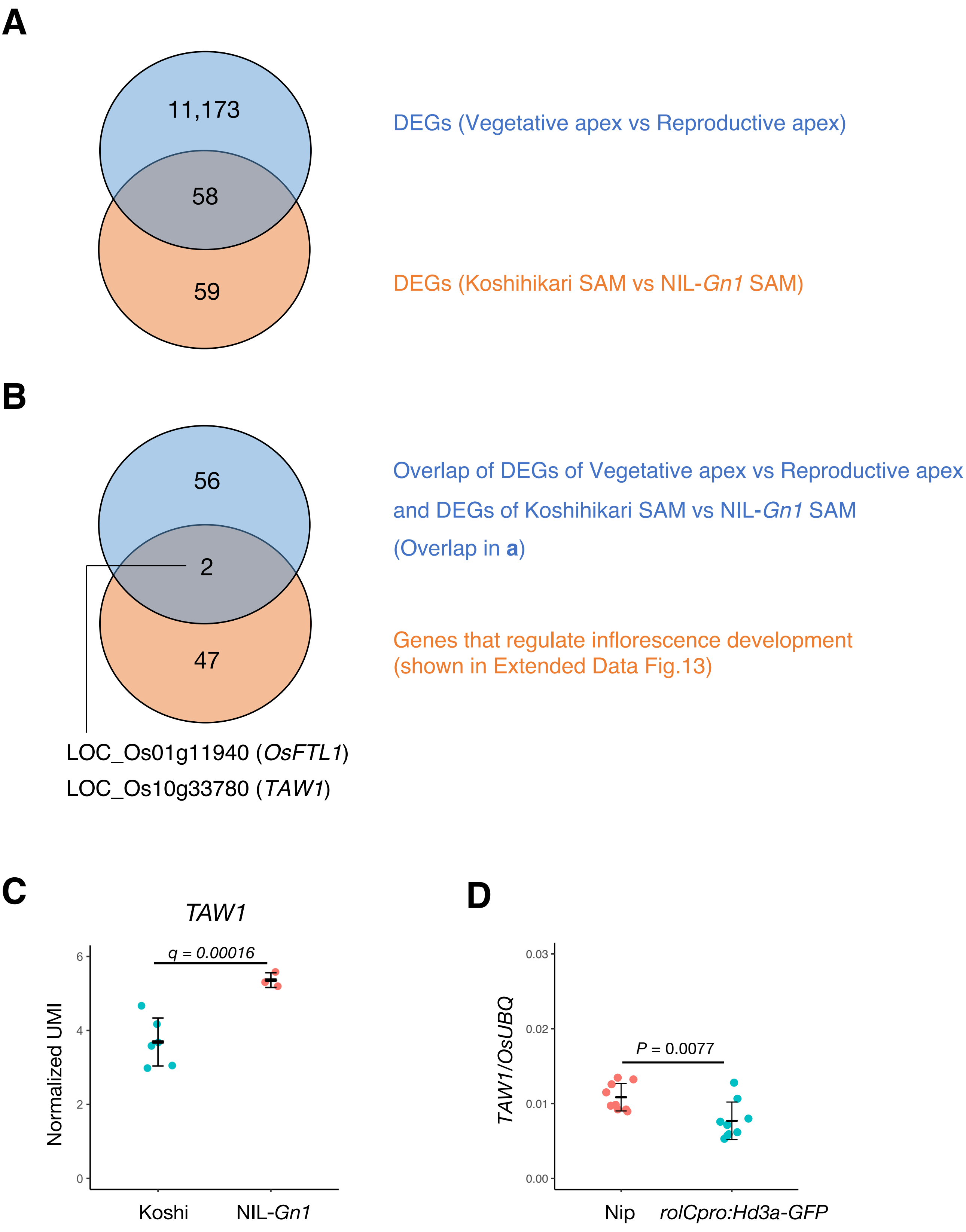
A
11,173
58
59
DEGs (Vegetative apex vs Reproductive apex)
DEGs (Koshihikari SAM vs NIL-*Gn1* SAM)
B
56
2
47
Overlap of DEGs of Vegetative apex vs Reproductive apex
and DEGs of Koshihikari SAM vs NIL-*Gn1* SAM
(Overlap in **a**)
Genes that regulate inflorescence development
(shown in Extended Data Fig.13)
LOC_Os01g11940 (*OsFTL1*)
LOC_Os10g33780 (*TAW1*)
C
TAW1
q = 0.00016
Normalized UMI
0
2
4
6
Koshi
NIL-*Gn1*
D
TAW1/OsUBQ
0.00
0.01
0.02
0.03
P = 0.0077
Nip
rolCpro:Hd3a-GFP

Fig. S11

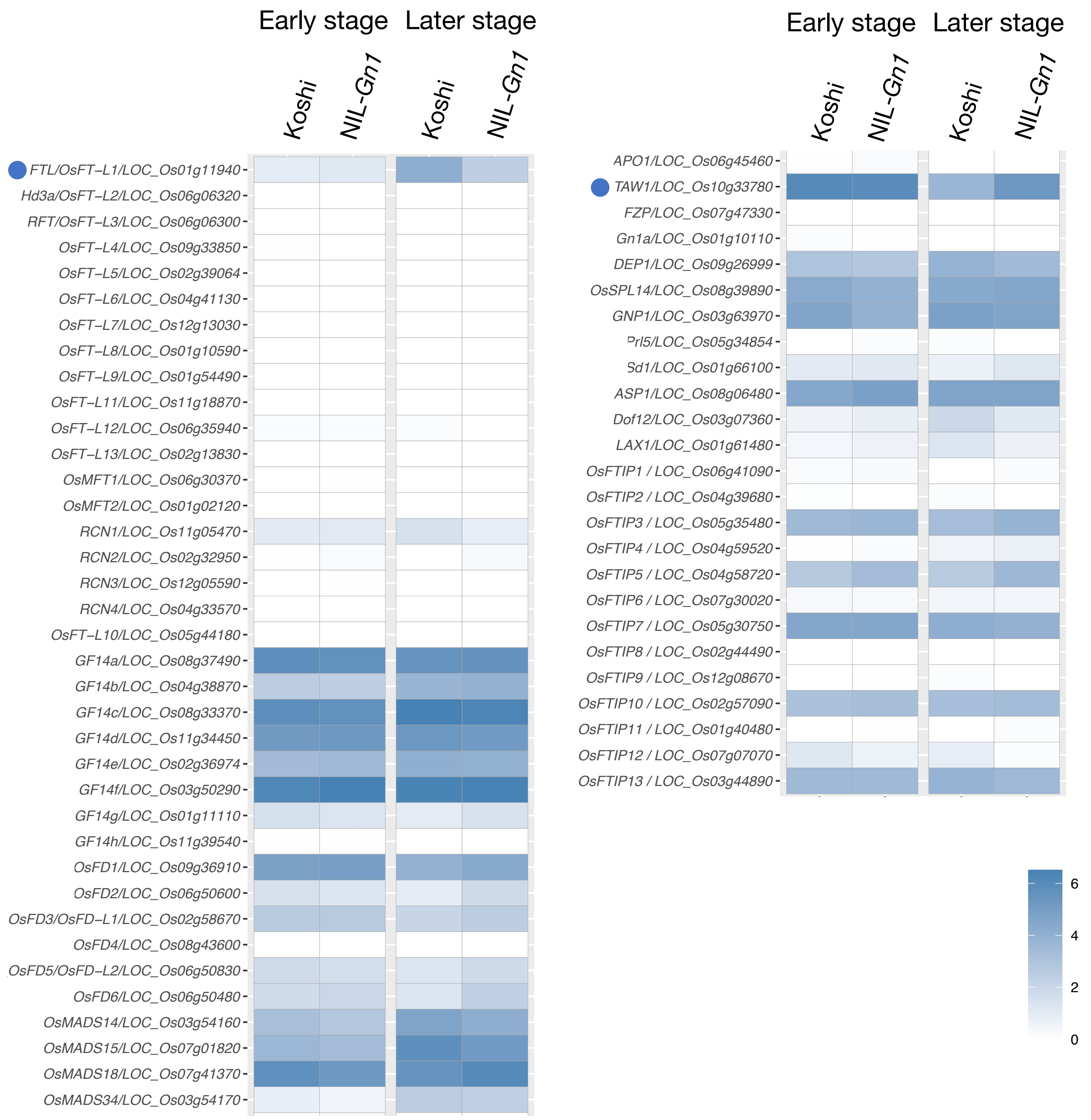

Early stage
Later stage
Koshi
NIL-Gn1
Koshi
NIL-Gn1
FTL/OsFT–L1/LOC_Os01g11940
Hd3a/OsFT–L2/LOC_Os06g06320
RFT/OsFT–L3/LOC_Os06g06300
OsFT–L4/LOC_Os09g33850
OsFT–L5/LOC_Os02g39064
OsFT–L6/LOC_Os04g41130
OsFT–L7/LOC_Os12g13030
OsFT–L8/LOC_Os01g10590
OsFT–L9/LOC_Os01g54490
OsFT–L11/LOC_Os11g18870
OsFT–L12/LOC_Os06g35940
OsFT–L13/LOC_Os02g13830
OsMFT1/LOC_Os06g30370
OsMFT2/LOC_Os01g02120
RCN1/LOC_Os11g05470
RCN2/LOC_Os02g32950
RCN3/LOC_Os12g05590
RCN4/LOC_Os04g33570
OsFT–L10/LOC_Os05g44180
GF14a/LOC_Os08g37490
GF14b/LOC_Os04g38870
GF14c/LOC_Os08g33370
GF14d/LOC_Os11g34450
GF14e/LOC_Os02g36974
GF14f/LOC_Os03g50290
GF14g/LOC_Os01g11110
GF14h/LOC_Os11g39540
OsFD1/LOC_Os09g36910
OsFD2/LOC_Os06g50600
OsFD3/OsFD–L1/LOC_Os02g58670
OsFD4/LOC_Os08g43600
OsFD5/OsFD–L2/LOC_Os06g50830
OsFD6/LOC_Os06g50480
OsMADS14/LOC_Os03g54160
OsMADS15/LOC_Os07g01820
OsMADS18/LOC_Os07g41370
OsMADS34/LOC_Os03g54170
Early stage
Later stage
Koshi
NIL-Gn1
Koshi
NIL-Gn1
APO1/LOC_Os06g45460
TAW1/LOC_Os10g33780
FZP/LOC_Os07g47330
Gn1a/LOC_Os01g10110
DEP1/LOC_Os09g26999
OsSPL14/LOC_Os08g39890
GNP1/LOC_Os03g63970
Prl5/LOC_Os05g34854
Sd1/LOC_Os01g66100
ASP1/LOC_Os08g06480
Dof12/LOC_Os03g07360
LAX1/LOC_Os01g61480
OsFTIP1 / LOC_Os06g41090
OsFTIP2 / LOC_Os04g39680
OsFTIP3 / LOC_Os05g35480
OsFTIP4 / LOC_Os04g59520
OsFTIP5 / LOC_Os04g58720
OsFTIP6 / LOC_Os07g30020
OsFTIP7 / LOC_Os05g30750
OsFTIP8 / LOC_Os02g44490
OsFTIP9 / LOC_Os12g08670
OsFTIP10 / LOC_Os02g57090
OsFTIP11 / LOC_Os01g40480
OsFTIP12 / LOC_Os07g07070
OsFTIP13 / LOC_Os03g44890
6
4
2
0

Fig. S12

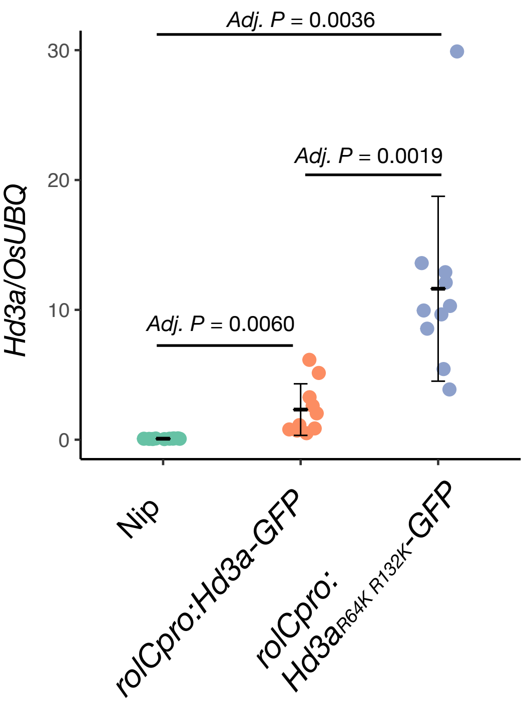

Adj. P = 0.0036
Adj. P = 0.0019
Adj. P = 0.0060
30
20
10
0
Hd3a/OsUBQ
Nip
rolCpro:Hd3a-GFP
rolCpro:
Hd3aR64K R132K-GFP

Fig. S13

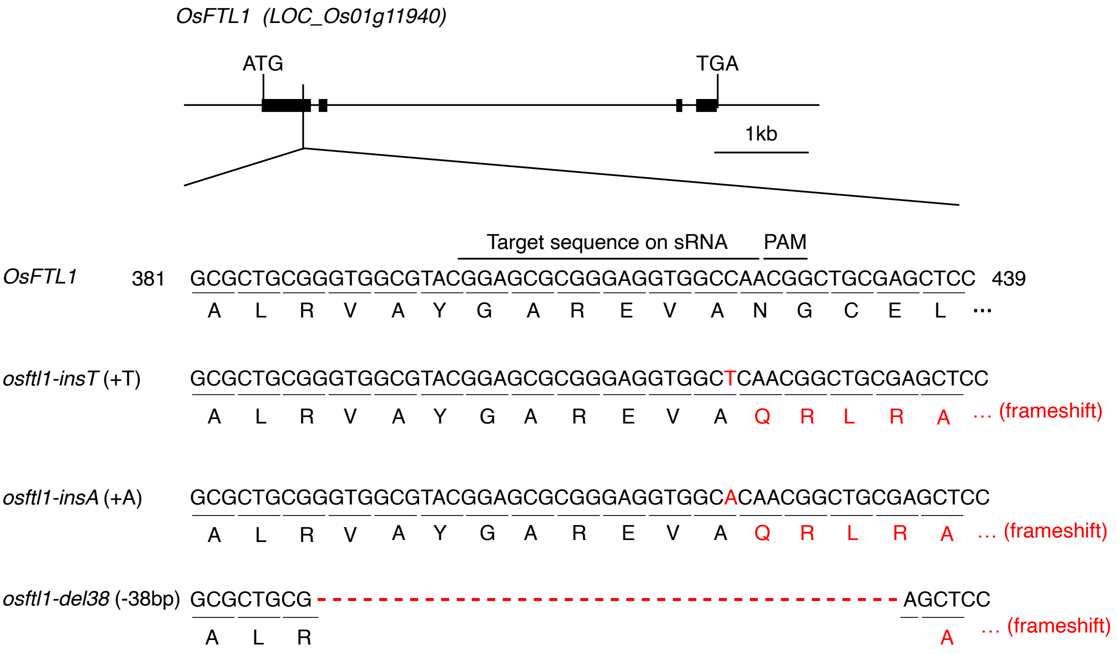

OsFTL1 (LOC_Os01g11940)
ATG
TGA
1kb
Target sequence on sRNA
PAM
OsFTL1 381 GCGCTGCGGGTGGCGTACGGAGCGCGGGAGGTGGCCAACGGCTGCGAGCTCC 439
A L R V A Y G A R E V A N G C E L …
osftl1-insT (+T) GCGCTGCGGGTGGCGTACGGAGCGCGGGAGGTGGCTCAACGGCTGCGAGCTCC
A L R V A Y G A R E V A Q R L R A … (frameshift)
osftl1-insA (+A) GCGCTGCGGGTGGCGTACGGAGCGCGGGAGGTGGCACAACGGCTGCGAGCTCC
A L R V A Y G A R E V A Q R L R A … (frameshift)
osftl1-del38 (-38bp) GCGCTGCG AGCTCC
A L R A … (frameshift)

Fig. S14

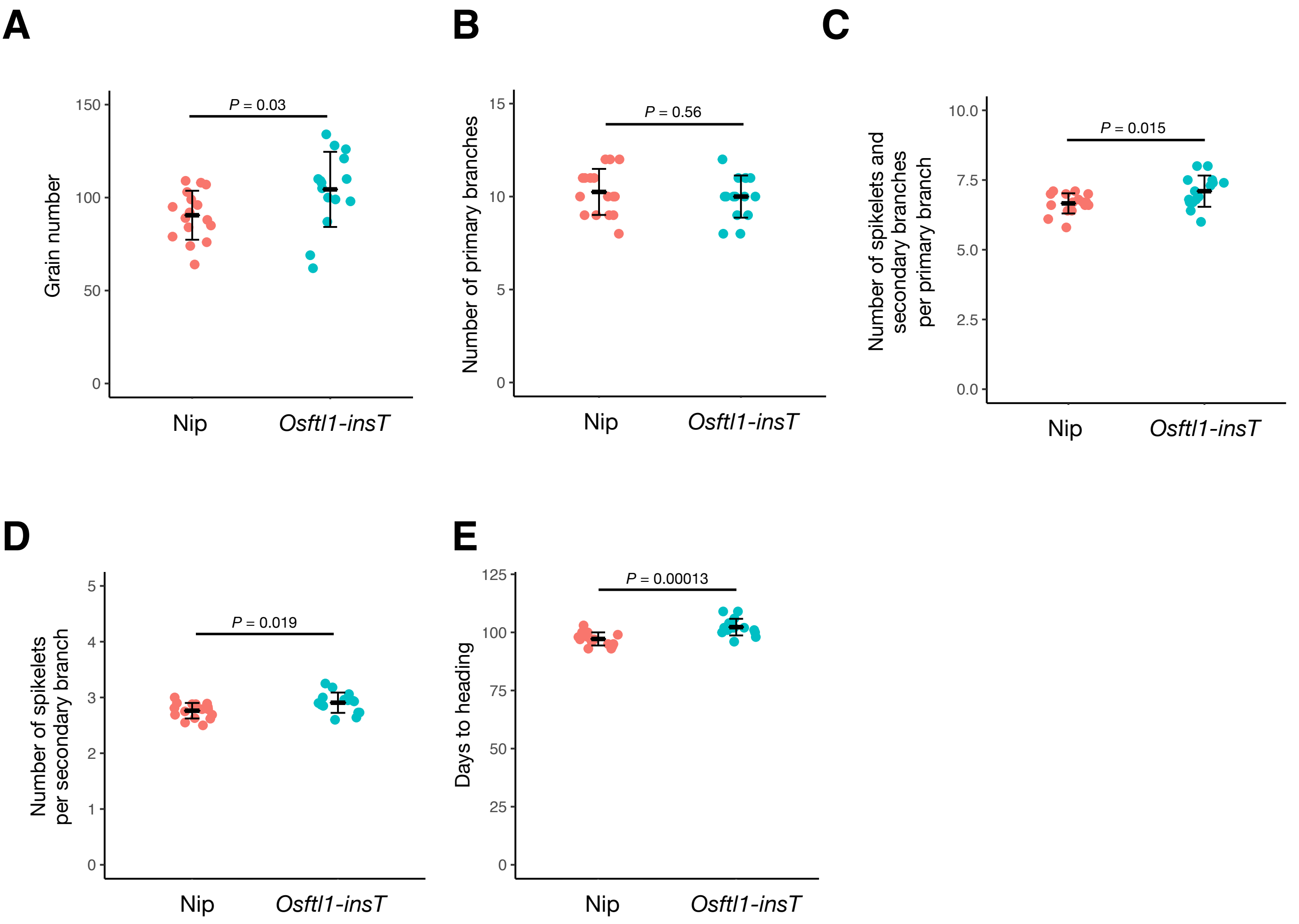
A
Grain number
P = 0.03
Nip
Osftl1-insT
B
Number of primary branches
P = 0.56
Nip
Osftl1-insT
C
Number of spikelets and secondary branches per primary branch
P = 0.015
Nip
Osftl1-insT
D
Number of spikelets per secondary branch
P = 0.019
Nip
Osftl1-insT
E
Days to heading
P = 0.00013
Nip
Osftl1-insT

Fig. S15

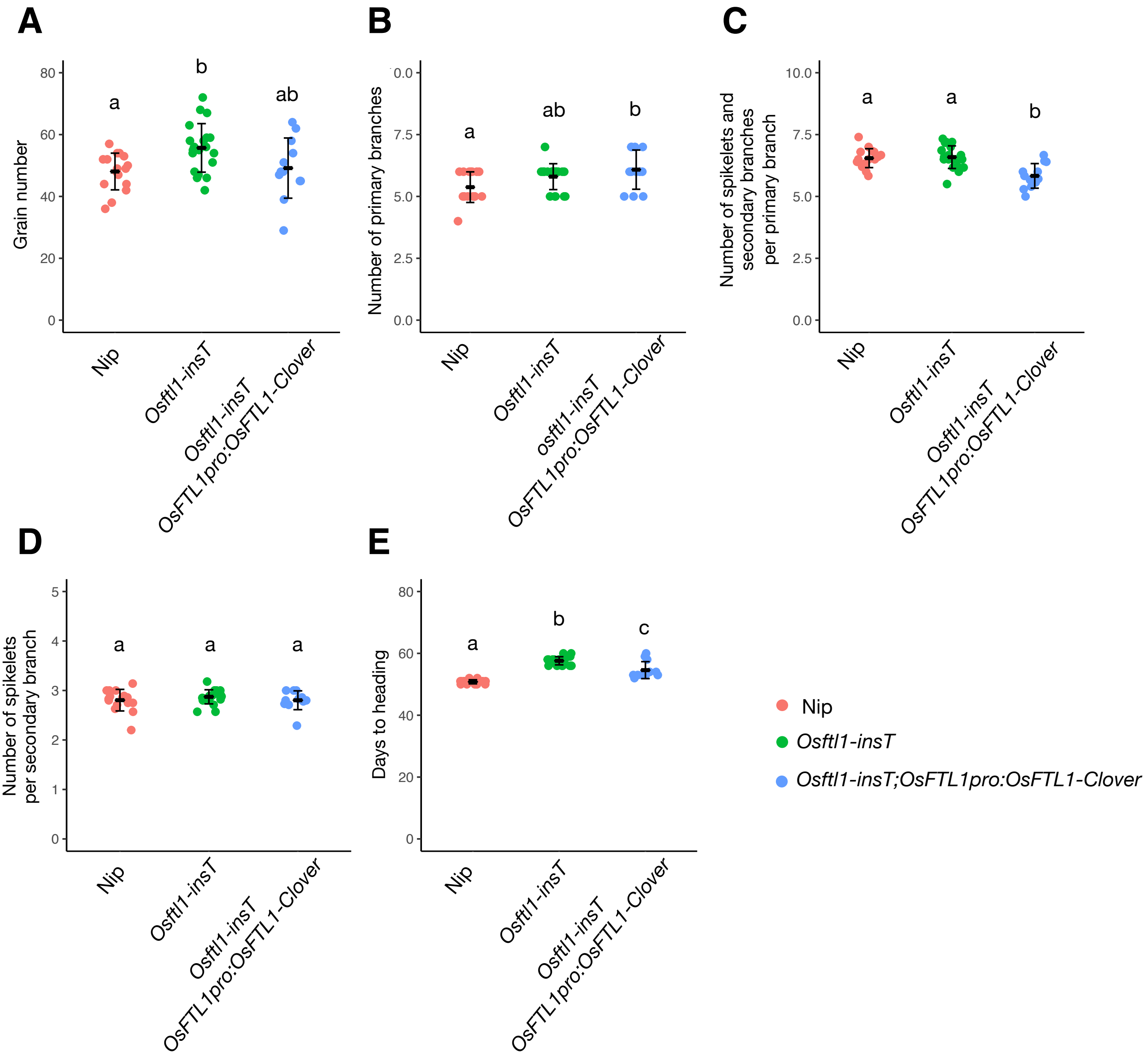
A
Grain number
Nip
Osftl1-insT
Osftl1-insT
OsFTL1pro:OsFTL1-Clover
B
Number of primary branches
Nip
Osftl1-insT
osftl1-insT
OsFTL1pro:OsFTL1-Clover
C
Number of spikelets and
secondary branches
per primary branch
Nip
Osftl1-insT
Osftl1-insT
OsFTL1pro:OsFTL1-Clover
D
Number of spikelets
per secondary branch
Nip
Osftl1-insT
Osftl1-insT
OsFTL1pro:OsFTL1-Clover
E
Days to heading
Nip
Osftl1-insT
Osftl1-insT
OsFTL1pro:OsFTL1-Clover
Nip
Osftl1-insT
Osftl1-insT;OsFTL1pro:OsFTL1-Clover

Fig. S16

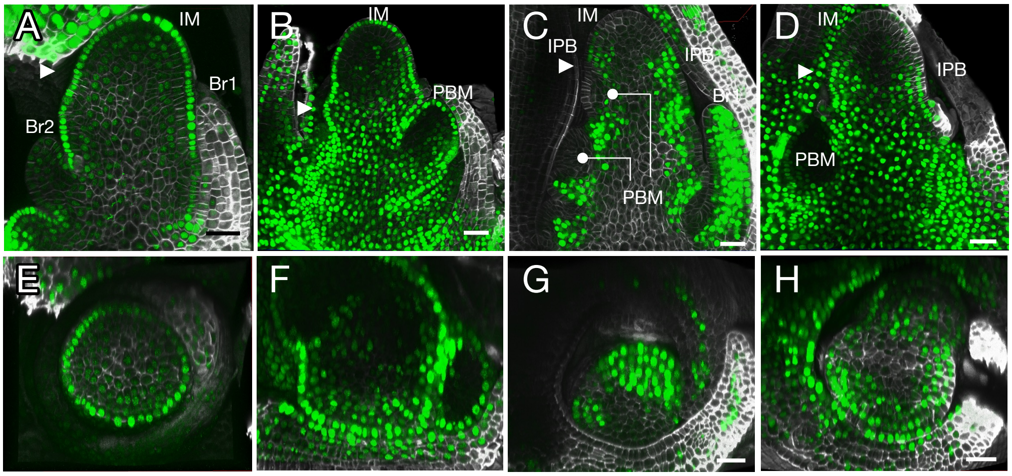

Fig. S17

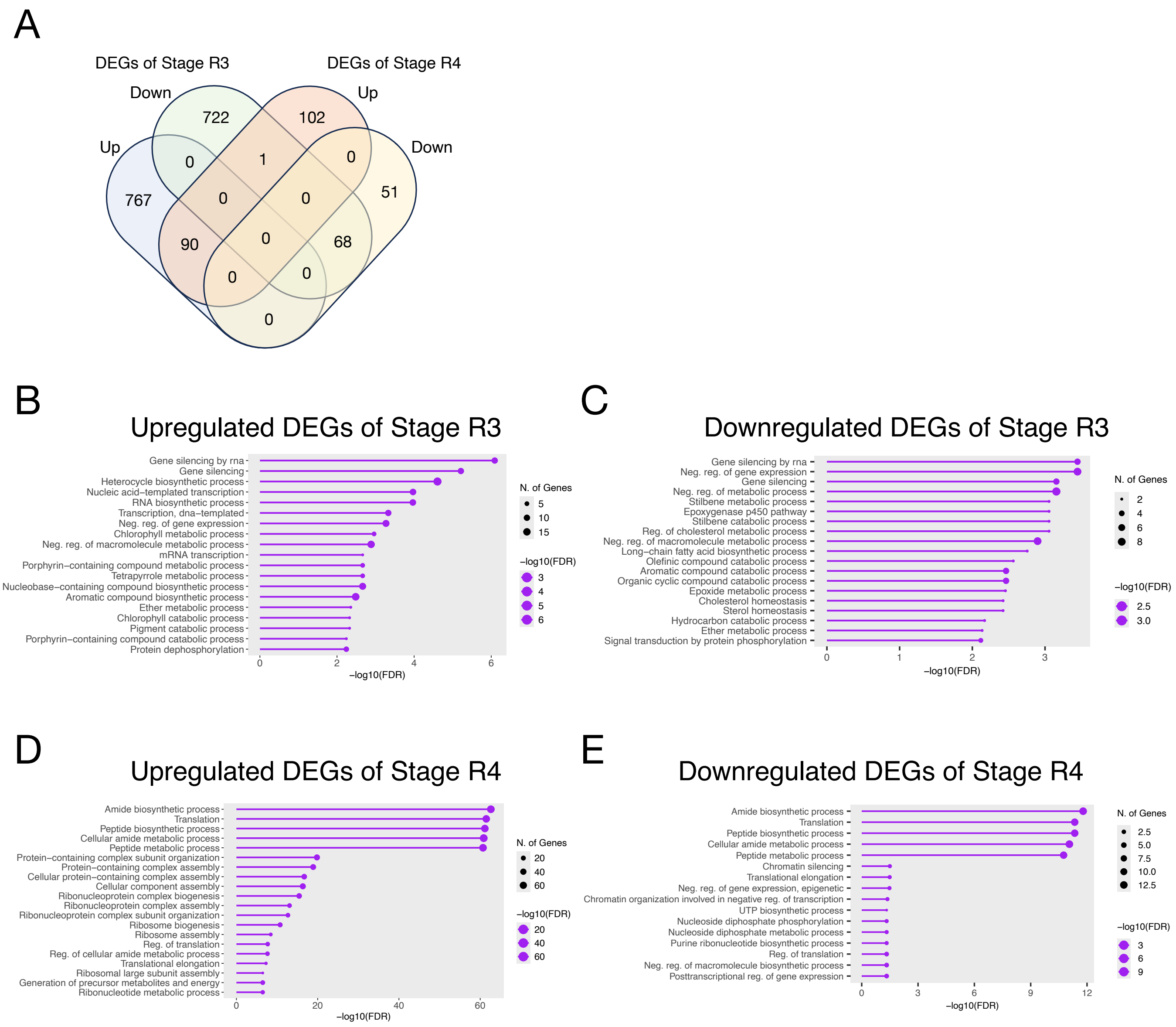
A
DEGs of Stage R3
DEGs of Stage R4
Down
Up
Up
Down
722
102
0
1
0
767
0
0
51
90
0
68
0
0
0
B
Upregulated DEGs of Stage R3
Gene silencing by rna
Gene silencing
Heterocycle biosynthetic process
Nucleic acid−templated transcription
RNA biosynthetic process
Transcription, dna−templated
Neg. reg. of gene expression
Chlorophyll metabolic process
Neg. reg. of macromolecule metabolic process
mRNA transcription
Porphyrin−containing compound metabolic process
Tetrapyrrole metabolic process
Nucleobase−containing compound biosynthetic process
Aromatic compound biosynthetic process
Ether metabolic process
Chlorophyll catabolic process
Pigment catabolic process
Porphyrin−containing compound catabolic process
Protein dephosphorylation
0
2
4
6
−log10(FDR)
N. of Genes
5
10
15
−log10(FDR)
3
4
5
6
C
Downregulated DEGs of Stage R3
Gene silencing by rna
Neg. reg. of gene expression
Gene silencing
Neg. reg. of metabolic process
Stilbene metabolic process
Epoxygenase p450 pathway
Stilbene catabolic process
Reg. of cholesterol metabolic process
Neg. reg. of macromolecule metabolic process
Long−chain fatty acid biosynthetic process
Olefinic compound catabolic process
Aromatic compound catabolic process
Organic cyclic compound catabolic process
Epoxide metabolic process
Cholesterol homeostasis
Sterol homeostasis
Hydrocarbon catabolic process
Ether metabolic process
Signal transduction by protein phosphorylation
0
1
2
3
−log10(FDR)
N. of Genes
2
4
6
8
−log10(FDR)
2.5
3.0
D
Upregulated DEGs of Stage R4
Amide biosynthetic process
Translation
Peptide biosynthetic process
Cellular amide metabolic process
Peptide metabolic process
Protein−containing complex subunit organization
Protein−containing complex assembly
Cellular protein−containing complex assembly
Cellular component assembly
Ribonucleoprotein complex biogenesis
Ribonucleoprotein complex assembly
Ribonucleoprotein complex subunit organization
Ribosome biogenesis
Ribosome assembly
Reg. of translation
Reg. of cellular amide metabolic process
Translational elongation
Ribosomal large subunit assembly
Generation of precursor metabolites and energy
Ribonucleotide metabolic process
0
20
40
60
−log10(FDR)
N. of Genes
20
40
60
−log10(FDR)
20
40
60
E
Downregulated DEGs of Stage R4
Amide biosynthetic process
Translation
Peptide biosynthetic process
Cellular amide metabolic process
Peptide metabolic process
Chromatin silencing
Translational elongation
Neg. reg. of gene expression, epigenetic
Chromatin organization involved in negative reg. of transcription
UTP biosynthetic process
Nucleoside diphosphate phosphorylation
Nucleoside diphosphate metabolic process
Purine ribonucleotide biosynthetic process
Reg. of translation
Neg. reg. of macromolecule biosynthetic process
Posttranscriptional reg. of gene expression
0
3
6
9
12
−log10(FDR)
N. of Genes
2.5
5.0
7.5
10.0
12.5
−log10(FDR)
3
6
9

Fig. S18

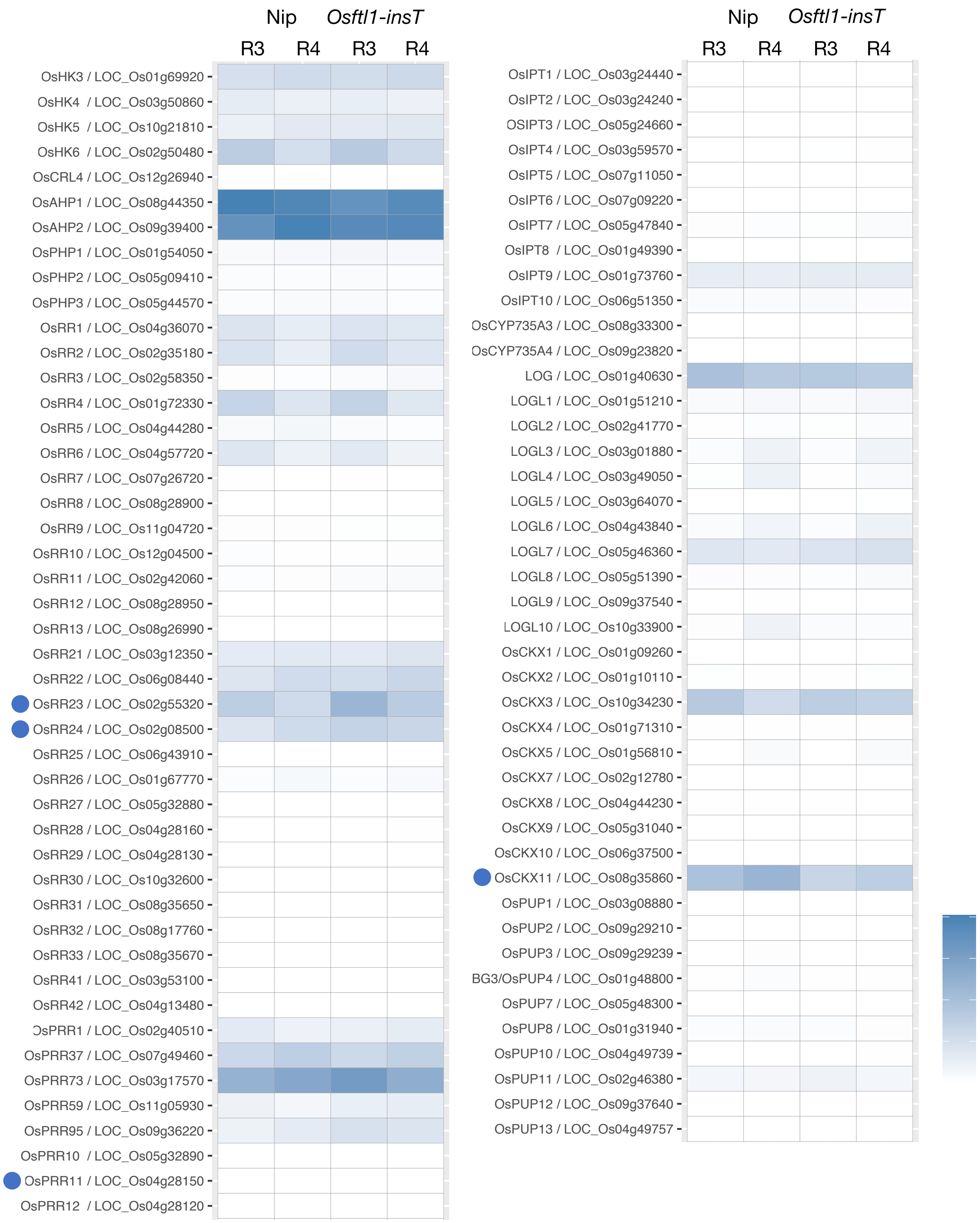

Nip
Osftl1-insT
R3
R4
R3
R4
OsHK3 / LOC_Os01g69920
OsHK4 / LOC_Os03g50860
OsHK5 / LOC_Os10g21810
OsHK6 / LOC_Os02g50480
OsCRL4 / LOC_Os12g26940
OsAHP1 / LOC_Os08g44350
OsAHP2 / LOC_Os09g39400
OsPHP1 / LOC_Os01g54050
OsPHP2 / LOC_Os05g09410
OsPHP3 / LOC_Os05g44570
OsRR1 / LOC_Os04g36070
OsRR2 / LOC_Os02g35180
OsRR3 / LOC_Os02g58350
OsRR4 / LOC_Os01g72330
OsRR5 / LOC_Os04g44280
OsRR6 / LOC_Os04g57720
OsRR7 / LOC_Os07g26720
OsRR8 / LOC_Os08g28900
OsRR9 / LOC_Os11g04720
OsRR10 / LOC_Os12g04500
OsRR11 / LOC_Os02g42060
OsRR12 / LOC_Os08g28950
OsRR13 / LOC_Os08g26990
OsRR21 / LOC_Os03g12350
OsRR22 / LOC_Os06g08440
OsRR23 / LOC_Os02g55320
OsRR24 / LOC_Os02g08500
OsRR25 / LOC_Os06g43910
OsRR26 / LOC_Os01g67770
OsRR27 / LOC_Os05g32880
OsRR28 / LOC_Os04g28160
OsRR29 / LOC_Os04g28130
OsRR30 / LOC_Os10g32600
OsRR31 / LOC_Os08g35650
OsRR32 / LOC_Os08g17760
OsRR33 / LOC_Os08g35670
OsRR41 / LOC_Os03g53100
OsRR42 / LOC_Os04g13480
OsPRR1 / LOC_Os02g40510
OsPRR37 / LOC_Os07g49460
OsPRR73 / LOC_Os03g17570
OsPRR59 / LOC_Os11g05930
OsPRR95 / LOC_Os09g36220
OsPRR10 / LOC_Os05g32890
OsPRR11 / LOC_Os04g28150
OsPRR12 / LOC_Os04g28120
Nip
Osftl1-insT
R3
R4
R3
R4
OsIPT1 / LOC_Os03g24440
OsIPT2 / LOC_Os03g24240
OSIPT3 / LOC_Os05g24660
OsIPT4 / LOC_Os03g59570
OsIPT5 / LOC_Os07g11050
OsIPT6 / LOC_Os07g09220
OsIPT7 / LOC_Os05g47840
OsIPT8 / LOC_Os01g49390
OsIPT9 / LOC_Os01g73760
OsIPT10 / LOC_Os06g51350
OsCYP735A3 / LOC_Os08g33300
OsCYP735A4 / LOC_Os09g23820
LOG / LOC_Os01g40630
LOGL1 / LOC_Os01g51210
LOGL2 / LOC_Os02g41770
LOGL3 / LOC_Os03g01880
LOGL4 / LOC_Os03g49050
LOGL5 / LOC_Os03g64070
LOGL6 / LOC_Os04g43840
LOGL7 / LOC_Os05g46360
LOGL8 / LOC_Os05g51390
LOGL9 / LOC_Os09g37540
LOGL10 / LOC_Os10g33900
OsCKX1 / LOC_Os01g09260
OsCKX2 / LOC_Os01g10110
OsCKX3 / LOC_Os10g34230
OsCKX4 / LOC_Os01g71310
OsCKX5 / LOC_Os01g56810
OsCKX7 / LOC_Os02g12780
OsCKX8 / LOC_Os04g44230
OsCKX9 / LOC_Os05g31040
OsCKX10 / LOC_Os06g37500
OsCKX11 / LOC_Os08g35860
OsPUP1 / LOC_Os03g08880
OsPUP2 / LOC_Os09g29210
OsPUP3 / LOC_Os09g29239
BG3/OsPUP4 / LOC_Os01g48800
OsPUP7 / LOC_Os05g48300
OsPUP8 / LOC_Os01g31940
OsPUP10 / LOC_Os04g49739
OsPUP11 / LOC_Os02g46380
OsPUP12 / LOC_Os09g37640
OsPUP13 / LOC_Os04g49757

Fig. S19

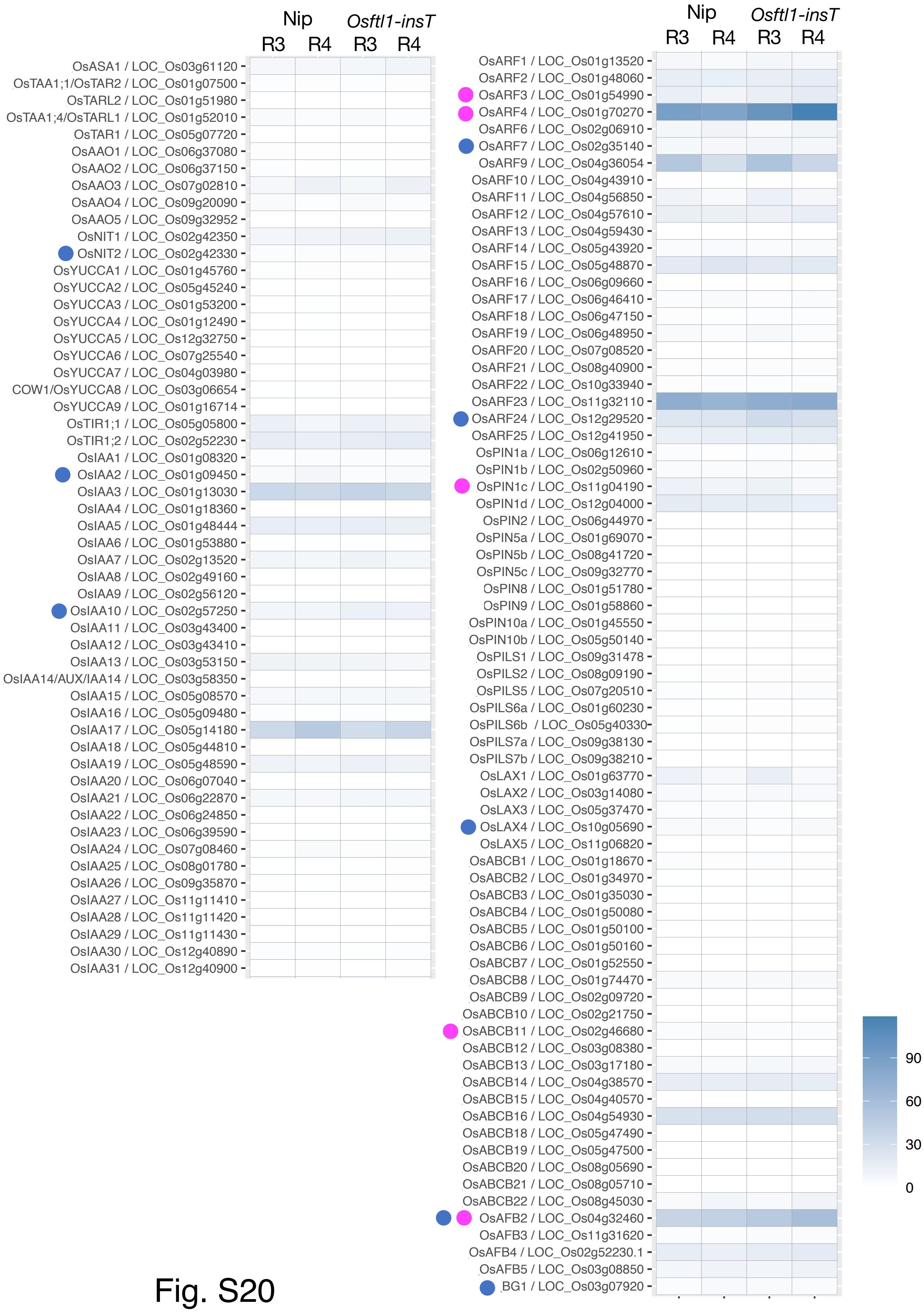

Nip
Osftl1-insT
R3
R4
R3
R4
OsASA1 / LOC_Os03g61120
OsTAA1;1/OsTAR2 / LOC_Os01g07500
OsTARL2 / LOC_Os01g51980
OsTAA1;4/OsTARL1 / LOC_Os01g52010
OsTAR1 / LOC_Os05g07720
OsAAO1 / LOC_Os06g37080
OsAAO2 / LOC_Os06g37150
OsAAO3 / LOC_Os07g02810
OsAAO4 / LOC_Os09g20090
OsAAO5 / LOC_Os09g32952
OsNIT1 / LOC_Os02g42350
OsNIT2 / LOC_Os02g42330
OsYUCCA1 / LOC_Os01g45760
OsYUCCA2 / LOC_Os05g45240
OsYUCCA3 / LOC_Os01g53200
OsYUCCA4 / LOC_Os01g12490
OsYUCCA5 / LOC_Os12g32750
OsYUCCA6 / LOC_Os07g25540
OsYUCCA7 / LOC_Os04g03980
COW1/OsYUCCA8 / LOC_Os03g06654
OsYUCCA9 / LOC_Os01g16714
OsTIR1;1 / LOC_Os05g05800
OsTIR1;2 / LOC_Os02g52230
OsIAA1 / LOC_Os01g08320
OsIAA2 / LOC_Os01g09450
OsIAA3 / LOC_Os01g13030
OsIAA4 / LOC_Os01g18360
OsIAA5 / LOC_Os01g48444
OsIAA6 / LOC_Os01g53880
OsIAA7 / LOC_Os02g13520
OsIAA8 / LOC_Os02g49160
OsIAA9 / LOC_Os02g56120
OsIAA10 / LOC_Os02g57250
OsIAA11 / LOC_Os03g43400
OsIAA12 / LOC_Os03g43410
OsIAA13 / LOC_Os03g53150
OsIAA14/AUX/IAA14 / LOC_Os03g58350
OsIAA15 / LOC_Os05g08570
OsIAA16 / LOC_Os05g09480
OsIAA17 / LOC_Os05g14180
OsIAA18 / LOC_Os05g44810
OsIAA19 / LOC_Os05g48590
OsIAA20 / LOC_Os06g07040
OsIAA21 / LOC_Os06g22870
OsIAA22 / LOC_Os06g24850
OsIAA23 / LOC_Os06g39590
OsIAA24 / LOC_Os07g08460
OsIAA25 / LOC_Os08g01780
OsIAA26 / LOC_Os09g35870
OsIAA27 / LOC_Os11g11410
OsIAA28 / LOC_Os11g11420
OsIAA29 / LOC_Os11g11430
OsIAA30 / LOC_Os12g40890
OsIAA31 / LOC_Os12g40900
Nip
Osftl1-insT
R3
R4
R3
R4
OsARF1 / LOC_Os01g13520
OsARF2 / LOC_Os01g48060
OsARF3 / LOC_Os01g54990
OsARF4 / LOC_Os01g70270
OsARF6 / LOC_Os02g06910
OsARF7 / LOC_Os02g35140
OsARF9 / LOC_Os04g36054
OsARF10 / LOC_Os04g43910
OsARF11 / LOC_Os04g56850
OsARF12 / LOC_Os04g57610
OsARF13 / LOC_Os04g59430
OsARF14 / LOC_Os05g43920
OsARF15 / LOC_Os05g48870
OsARF16 / LOC_Os06g09660
OsARF17 / LOC_Os06g46410
OsARF18 / LOC_Os06g47150
OsARF19 / LOC_Os06g48950
OsARF20 / LOC_Os07g08520
OsARF21 / LOC_Os08g40900
OsARF22 / LOC_Os10g33940
OsARF23 / LOC_Os11g32110
OsARF24 / LOC_Os12g29520
OsARF25 / LOC_Os12g41950
OsPIN1a / LOC_Os06g12610
OsPIN1b / LOC_Os02g50960
OsPIN1c / LOC_Os11g04190
OsPIN1d / LOC_Os12g04000
OsPIN2 / LOC_Os06g44970
OsPIN5a / LOC_Os01g69070
OsPIN5b / LOC_Os08g41720
OsPIN5c / LOC_Os09g32770
OsPIN8 / LOC_Os01g51780
OsPIN9 / LOC_Os01g58860
OsPIN10a / LOC_Os01g45550
OsPIN10b / LOC_Os05g50140
OsPILS1 / LOC_Os09g31478
OsPILS2 / LOC_Os08g09190
OsPILS5 / LOC_Os07g20510
OsPILS6a / LOC_Os01g60230
OsPILS6b / LOC_Os05g40330
OsPILS7a / LOC_Os09g38130
OsPILS7b / LOC_Os09g38210
OsLAX1 / LOC_Os01g63770
OsLAX2 / LOC_Os03g14080
OsLAX3 / LOC_Os05g37470
OsLAX4 / LOC_Os10g05690
OsLAX5 / LOC_Os11g06820
OsABCB1 / LOC_Os01g18670
OsABCB2 / LOC_Os01g34970
OsABCB3 / LOC_Os01g35030
OsABCB4 / LOC_Os01g50080
OsABCB5 / LOC_Os01g50100
OsABCB6 / LOC_Os01g50160
OsABCB7 / LOC_Os01g52550
OsABCB8 / LOC_Os01g74470
OsABCB9 / LOC_Os02g09720
OsABCB10 / LOC_Os02g21750
OsABCB11 / LOC_Os02g46680
OsABCB12 / LOC_Os03g08380
OsABCB13 / LOC_Os03g17180
OsABCB14 / LOC_Os04g38570
OsABCB15 / LOC_Os04g40570
OsABCB16 / LOC_Os04g54930
OsABCB18 / LOC_Os05g47490
OsABCB19 / LOC_Os05g47500
OsABCB20 / LOC_Os08g05690
OsABCB21 / LOC_Os08g05710
OsABCB22 / LOC_Os08g45030
OsAFB2 / LOC_Os04g32460
OsAFB3 / LOC_Os11g31620
OsAFB4 / LOC_Os02g52230.1
OsAFB5 / LOC_Os03g08850
BG1 / LOC_Os03g07920
90
60
30
0

Fig. S20

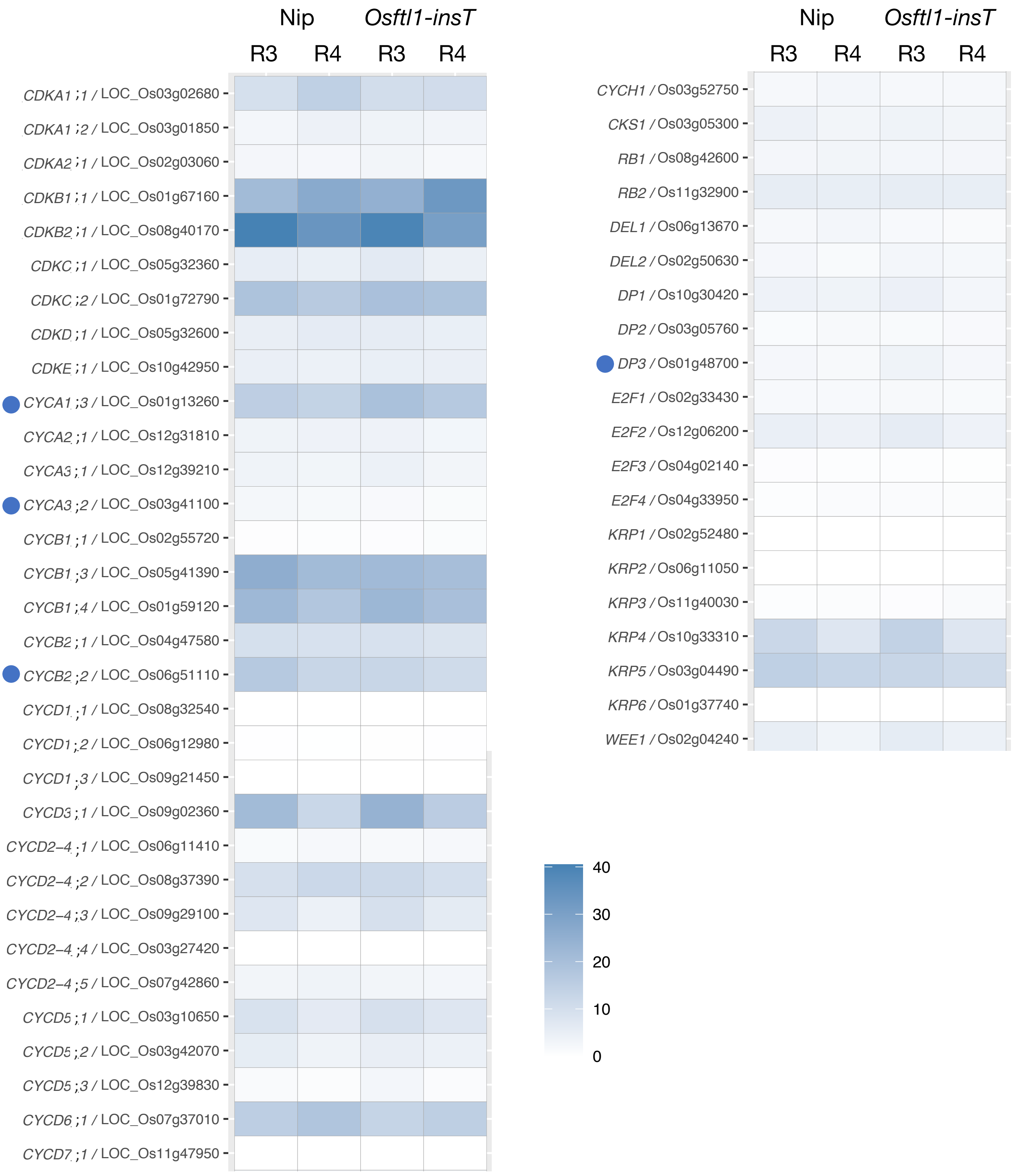
Nip
Osftl1-insT
R3
R4
R3
R4
CDKA1;1 / LOC_Os03g02680
CDKA1;2 / LOC_Os03g01850
CDKA2;1 / LOC_Os02g03060
CDKB1;1 / LOC_Os01g67160
CDKB2;1 / LOC_Os08g40170
CDKC;1 / LOC_Os05g32360
CDKC;2 / LOC_Os01g72790
CDKD;1 / LOC_Os05g32600
CDKE;1 / LOC_Os10g42950
CYCA1;3 / LOC_Os01g13260
CYCA2;1 / LOC_Os12g31810
CYCA3;1 / LOC_Os12g39210
CYCA3;2 / LOC_Os03g41100
CYCB1;1 / LOC_Os02g55720
CYCB1;3 / LOC_Os05g41390
CYCB1;4 / LOC_Os01g59120
CYCB2;1 / LOC_Os04g47580
CYCB2;2 / LOC_Os06g51110
CYCD1;1 / LOC_Os08g32540
CYCD1;2 / LOC_Os06g12980
CYCD1;3 / LOC_Os09g21450
CYCD3;1 / LOC_Os09g02360
CYCD2–4;1 / LOC_Os06g11410
CYCD2–4;2 / LOC_Os08g37390
CYCD2–4;3 / LOC_Os09g29100
CYCD2–4;4 / LOC_Os03g27420
CYCD2–4;5 / LOC_Os07g42860
CYCD5;1 / LOC_Os03g10650
CYCD5;2 / LOC_Os03g42070
CYCD5;3 / LOC_Os12g39830
CYCD6;1 / LOC_Os07g37010
CYCD7;1 / LOC_Os11g47950
CYCH1 / Os03g52750
CKS1 / Os03g05300
RB1 / Os08g42600
RB2 / Os11g32900
DEL1 / Os06g13670
DEL2 / Os02g50630
DP1 / Os10g30420
DP2 / Os03g05760
DP3 / Os01g48700
E2F1 / Os02g33430
E2F2 / Os12g06200
E2F3 / Os04g02140
E2F4 / Os04g33950
KRP1 / Os02g52480
KRP2 / Os06g11050
KRP3 / Os11g40030
KRP4 / Os10g33310
KRP5 / Os03g04490
KRP6 / Os01g37740
WEE1 / Os02g04240
40
30
20
10
0

Fig. S21

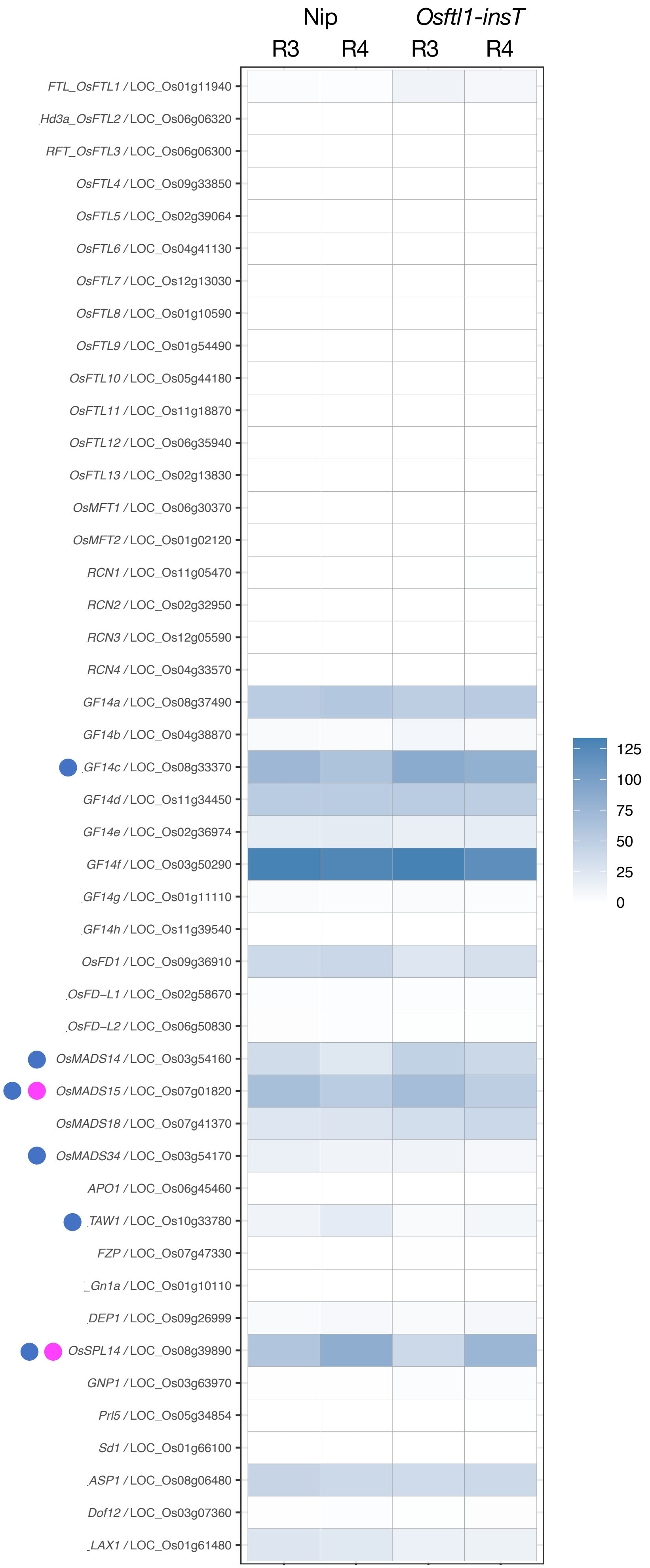
Nip
Osftl1-insT
R3
R4
R3
R4
FTL_OsFTL1 / LOC_Os01g11940
Hd3a_OsFTL2 / LOC_Os06g06320
RFT_OsFTL3 / LOC_Os06g06300
OsFTL4 / LOC_Os09g33850
OsFTL5 / LOC_Os02g39064
OsFTL6 / LOC_Os04g41130
OsFTL7 / LOC_Os12g13030
OsFTL8 / LOC_Os01g10590
OsFTL9 / LOC_Os01g54490
OsFTL10 / LOC_Os05g44180
OsFTL11 / LOC_Os11g18870
OsFTL12 / LOC_Os06g35940
OsFTL13 / LOC_Os02g13830
OsMFT1 / LOC_Os06g30370
OsMFT2 / LOC_Os01g02120
RCN1 / LOC_Os11g05470
RCN2 / LOC_Os02g32950
RCN3 / LOC_Os12g05590
RCN4 / LOC_Os04g33570
GF14a / LOC_Os08g37490
GF14b / LOC_Os04g38870
GF14c / LOC_Os08g33370
GF14d / LOC_Os11g34450
GF14e / LOC_Os02g36974
GF14f / LOC_Os03g50290
GF14g / LOC_Os01g11110
GF14h / LOC_Os11g39540
OsFD1 / LOC_Os09g36910
OsFD–L1 / LOC_Os02g58670
OsFD–L2 / LOC_Os06g50830
OsMADS14 / LOC_Os03g54160
OsMADS15 / LOC_Os07g01820
OsMADS18 / LOC_Os07g41370
OsMADS34 / LOC_Os03g54170
APO1 / LOC_Os06g45460
TAW1 / LOC_Os10g33780
FZP / LOC_Os07g47330
Gn1a / LOC_Os01g10110
DEP1 / LOC_Os09g26999
OsSPL14 / LOC_Os08g39890
GNP1 / LOC_Os03g63970
Prl5 / LOC_Os05g34854
Sd1 / LOC_Os01g66100
ASP1 / LOC_Os08g06480
Dof12 / LOC_Os03g07360
LAX1 / LOC_Os01g61480
125
100
75
50
25
0

Fig. S22

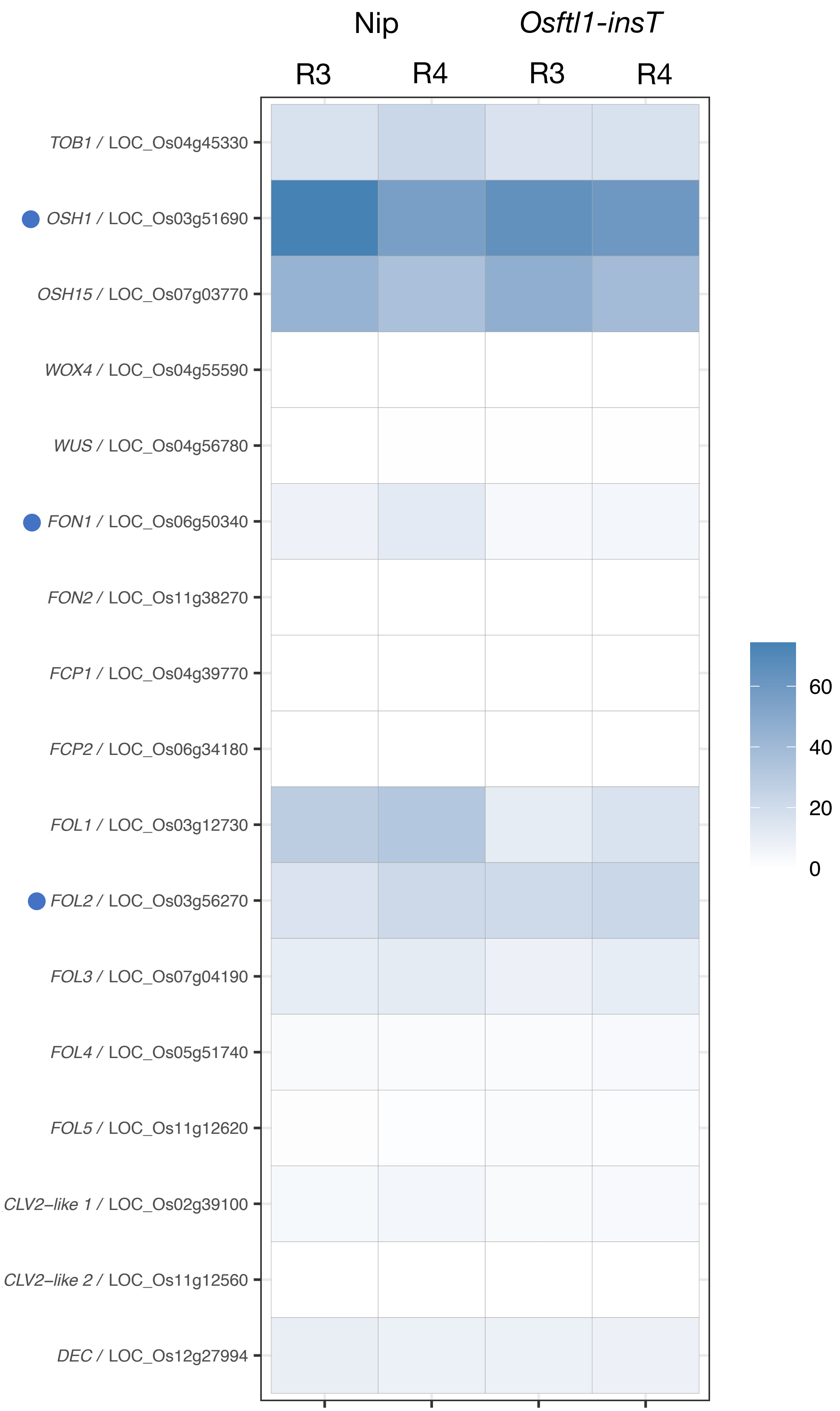

Nip
Osftl1-insT
R3
R4
R3
R4
TOB1 / LOC_Os04g45330
OSH1 / LOC_Os03g51690
OSH15 / LOC_Os07g03770
WOX4 / LOC_Os04g55590
WUS / LOC_Os04g56780
FON1 / LOC_Os06g50340
FON2 / LOC_Os11g38270
FCP1 / LOC_Os04g39770
FCP2 / LOC_Os06g34180
FOL1 / LOC_Os03g12730
FOL2 / LOC_Os03g56270
FOL3 / LOC_Os07g04190
FOL4 / LOC_Os05g51740
FOL5 / LOC_Os11g12620
CLV2–like 1 / LOC_Os02g39100
CLV2–like 2 / LOC_Os11g12560
DEC / LOC_Os12g27994
60
40
20
0

Fig. S23

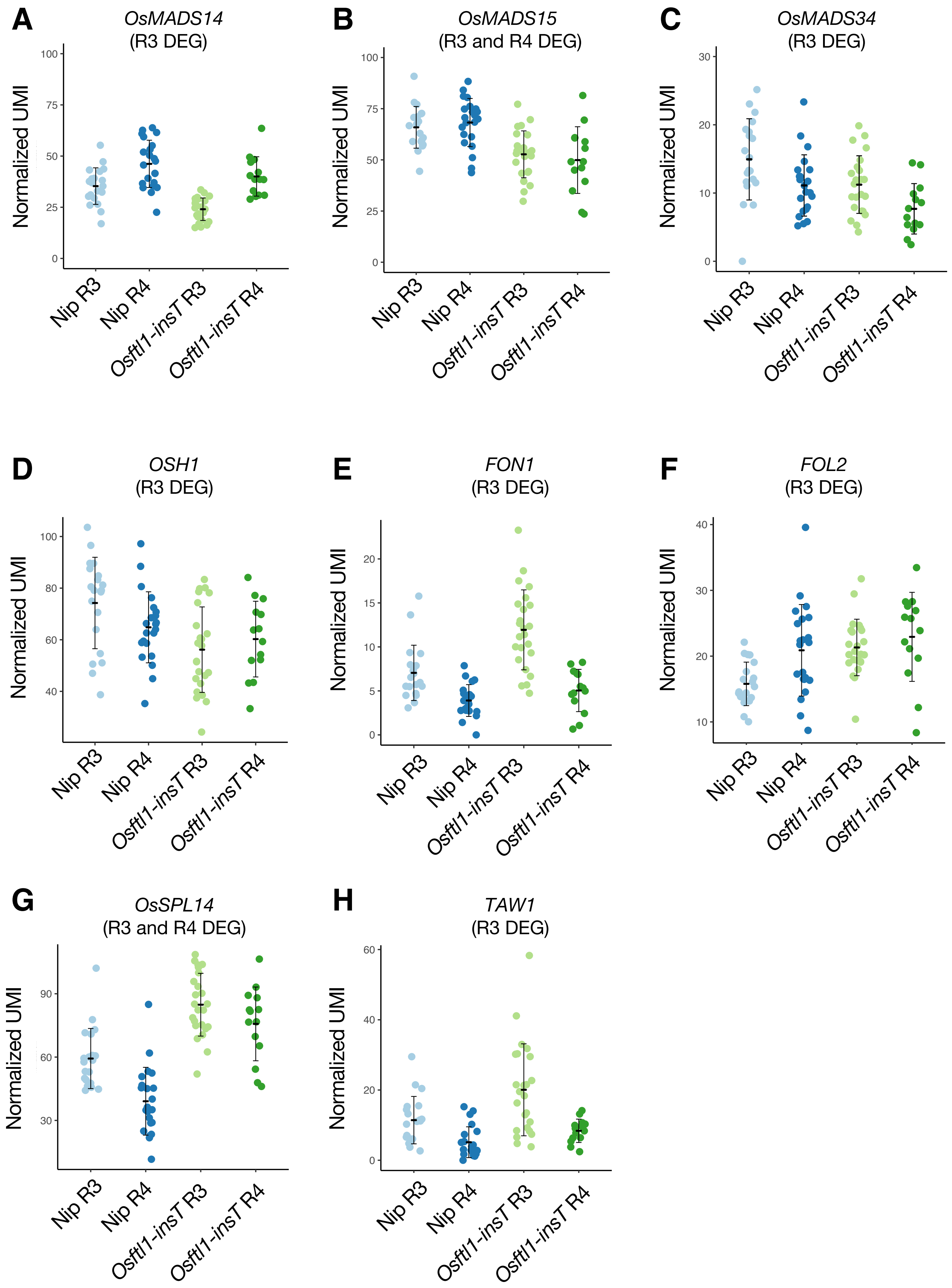
A
OsMADS14
(R3 DEG)
Normalized UMI
100
75
50
25
0
B
OsMADS15
(R3 and R4 DEG)
Normalized UMI
100
75
50
25
0
C
OsMADS34
(R3 DEG)
Normalized UMI
30
20
10
0
D
OSH1
(R3 DEG)
Normalized UMI
100
80
60
40
E
FON1
(R3 DEG)
Normalized UMI
20
15
10
5
0
F
FOL2
(R3 DEG)
Normalized UMI
40
30
20
10
G
OsSPL14
(R3 and R4 DEG)
Normalized UMI
90
60
30
H
TAW1
(R3 DEG)
Normalized UMI
60
40
20
0
Nip R3
Nip R4
Osftl1-insT R3
Osftl1-insT R4

Fig. S24

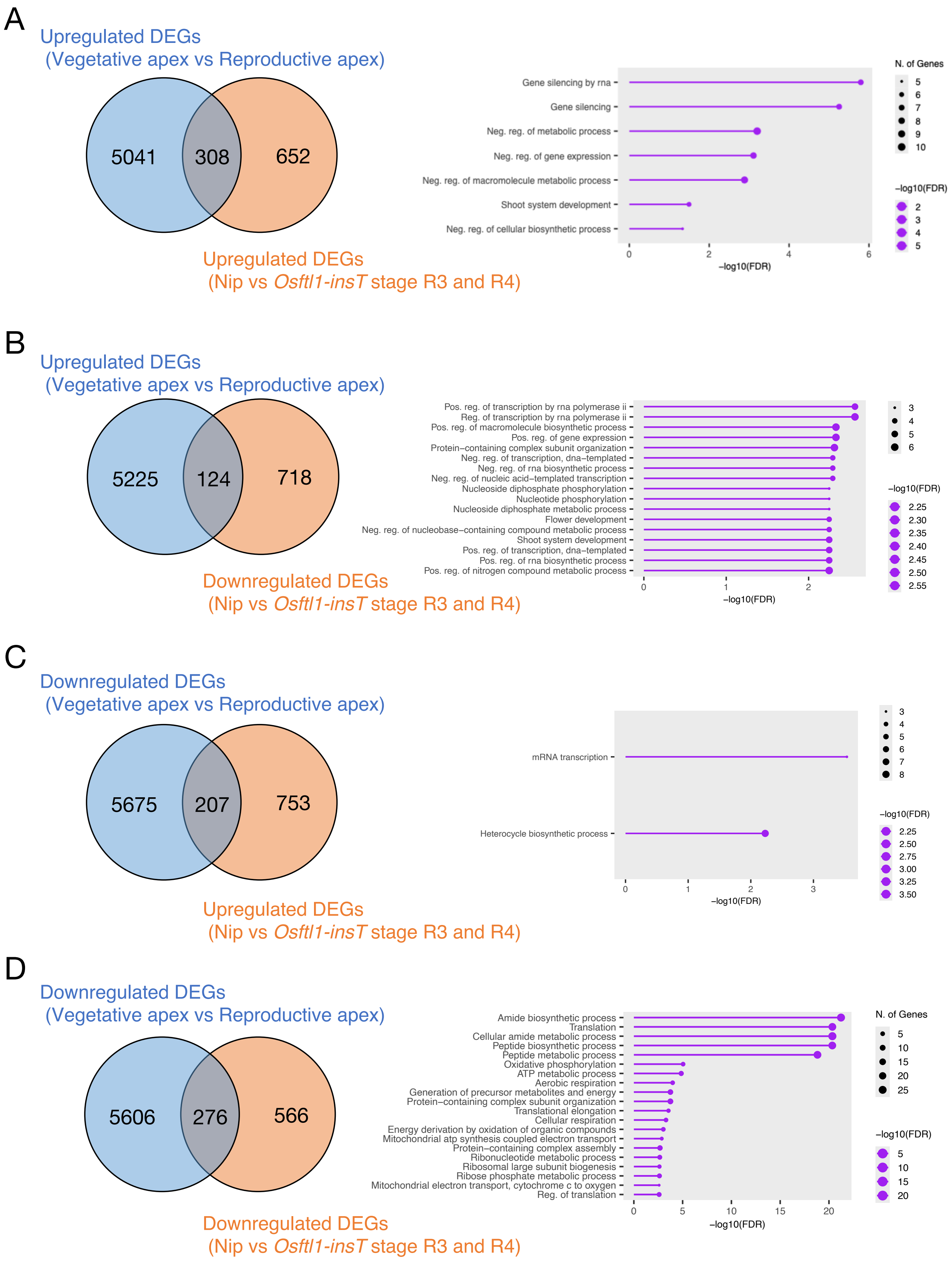
A
Upregulated DEGs
(Vegetative apex vs Reproductive apex)
5041
308
652
Upregulated DEGs
(Nip vs *Osftl1-insT* stage R3 and R4)
Gene silencing by rna
Gene silencing
Neg. reg. of metabolic process
Neg. reg. of gene expression
Neg. reg. of macromolecule metabolic process
Shoot system development
Neg. reg. of cellular biosynthetic process
0
2
4
6
−log10(FDR)
N. of Genes
5
6
7
8
9
10
−log10(FDR)
2
3
4
5
B
Upregulated DEGs
(Vegetative apex vs Reproductive apex)
5225
124
718
Downregulated DEGs
(Nip vs *Osftl1-insT* stage R3 and R4)
Pos. reg. of transcription by rna polymerase ii
Reg. of transcription by rna polymerase ii
Pos. reg. of macromolecule biosynthetic process
Pos. reg. of gene expression
Protein−containing complex subunit organization
Neg. reg. of transcription, dna−templated
Neg. reg. of rna biosynthetic process
Neg. reg. of nucleic acid−templated transcription
Nucleoside diphosphate phosphorylation
Nucleotide phosphorylation
Nucleoside diphosphate metabolic process
Flower development
Neg. reg. of nucleobase−containing compound metabolic process
Shoot system development
Pos. reg. of transcription, dna−templated
Pos. reg. of rna biosynthetic process
Pos. reg. of nitrogen compound metabolic process
0
1
2
−log10(FDR)
3
4
5
6
−log10(FDR)
2.25
2.30
2.35
2.40
2.45
2.50
2.55
C
Downregulated DEGs
(Vegetative apex vs Reproductive apex)
5675
207
753
Upregulated DEGs
(Nip vs *Osftl1-insT* stage R3 and R4)
mRNA transcription
Heterocycle biosynthetic process
0
1
2
3
−log10(FDR)
3
4
5
6
7
8
−log10(FDR)
2.25
2.50
2.75
3.00
3.25
3.50
D
Downregulated DEGs
(Vegetative apex vs Reproductive apex)
5606
276
566
Downregulated DEGs
(Nip vs *Osftl1-insT* stage R3 and R4)
Amide biosynthetic process
Translation
Cellular amide metabolic process
Peptide biosynthetic process
Peptide metabolic process
Oxidative phosphorylation
ATP metabolic process
Aerobic respiration
Generation of precursor metabolites and energy
Protein−containing complex subunit organization
Translational elongation
Cellular respiration
Energy derivation by oxidation of organic compounds
Mitochondrial atp synthesis coupled electron transport
Protein−containing complex assembly
Ribonucleotide metabolic process
Ribosomal large subunit biogenesis
Ribose phosphate metabolic process
Mitochondrial electron transport, cytochrome c to oxygen
Reg. of translation
0
5
10
15
20
−log10(FDR)
N. of Genes
5
10
15
20
25
−log10(FDR)
5
10
15
20

Fig. S25

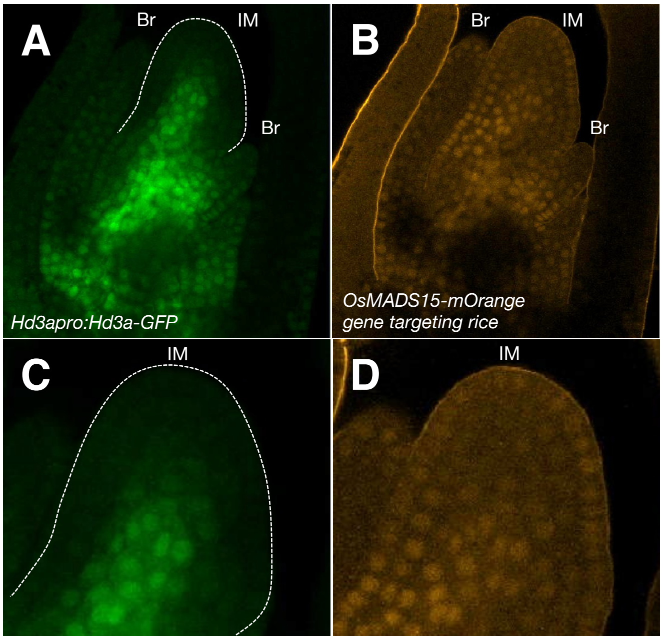
A
Br
IM
Br
Hd3apro:Hd3a-GFP
B
Br
IM
Br
OsMADS15-mOrange
gene targeting rice
C
IM
D
IM

Fig. S26

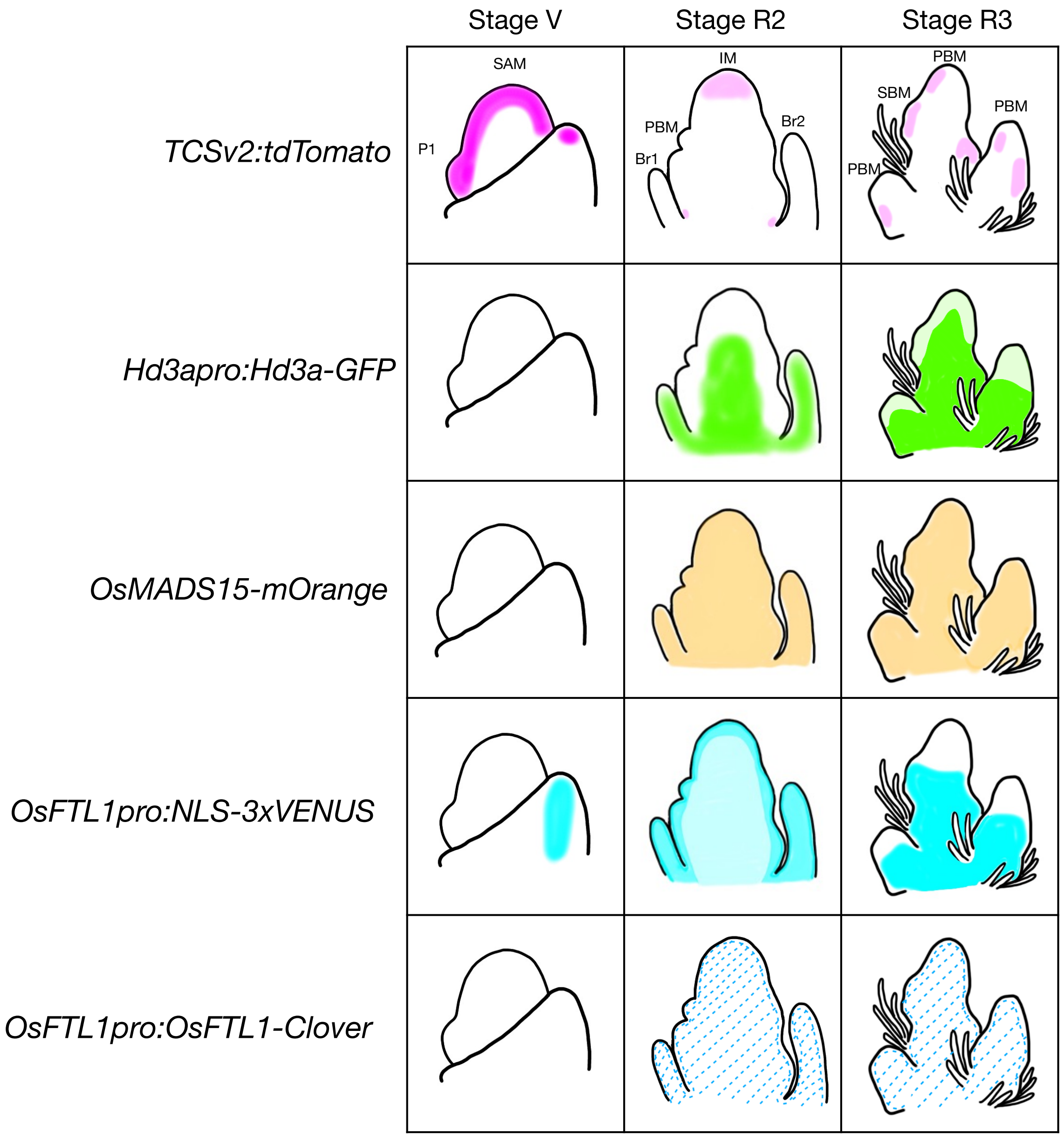
Stage V
Stage R2
Stage R3
TCSv2:tdTomato
Hd3apro:Hd3a-GFP
OsMADS15-mOrange
OsFTL1pro:NLS-3xVENUS
OsFTL1pro:OsFTL1-Clover
SAM
P1
IM
PBM
Br1
Br2
PBM
SBM
PBM
PBM

Fig. S27
